# Longitudinal Patterns of Within- and Cross-Domain Multimorbidity Across Physical, Psychological, and Cognitive Conditions in China and the United States: The Role of Socioeconomic and Healthcare Inequalities

**DOI:** 10.3390/healthcare14152326

**Published:** 2026-08-01

**Authors:** Meng Jia, Yingni Yu, Shu Su

**Affiliations:** 1 Department of Epidemiology and Biostatistics, The Second Affiliated Hospital of Chongqing Medical University, Chongqing 400010, China; 2 The Second Clinical College, Chongqing Medical University, Chongqing 400016, China; 3 Citrus Research Institute, Southwest University, Chongqing 400712, China; 4 School of Public Health, Chongqing Medical University, Chongqing 401331, China

**Keywords:** cross-domain multimorbidity, longitudinal study, psychological conditions, socioeconomic disparities, healthcare access

## Abstract

**Background**: Multimorbidity is a growing global health challenge in aging populations, yet its progression across physical, psychological, and cognitive domains and its contribution to health inequalities remain unclear. We compared longitudinal patterns of multimorbidity in China and the United States (US) and examined the socioeconomic and healthcare-related factors associated with these patterns and their functional consequences. **Methods**: This longitudinal cohort study included adults aged ≥45 years from China (2011–2020) and the US (2012–2020), matched 1:1 by baseline age and sex. Multimorbidity was classified into eight domains spanning physical, psychological, and cognitive conditions and their combinations. Longitudinal changes across five survey waves were assessed. Multinomial logistic mixed models examined socioeconomic and healthcare-related correlations, and Cox models estimated associations with subsequent limitations in activities of daily living (ADL) limitations and instrumental activities of daily living (IADL) limitations. **Results**: A total of 7064 participants from China (mean age 60.15 ± 7.93 years; 45.4% male) and 7064 matched participants from the US were included. Multimorbidity patterns were more complex in the US at baseline, but progression toward cross-domain multimorbidity occurred in both countries and was more pronounced in China. Psychological conditions occupied a central position in the development of more complex multimorbidity patterns. Higher educational and household wealth were consistently associated with lower odds of cognitive-related and cross-domain multimorbidity in both countries, whereas associations with healthcare-related factors varied across settings. Cross-domain multimorbidity was more strongly associated with functional limitations than within-domain multimorbidity. In particular, physical–psychological–cognitive multimorbidity was associated with substantially higher risks of ADL limitations (hazard ratio (HR)  =  5.70, 95% confidence interval (CI)  =  4.87–6.67 in China; HR  =  7.72, 95% CI  =  5.90–10.11 in the US) and IADL limitations (HR  =  4.14, 95% CI  =  3.64–4.71; HR  =  5.84, 95% CI  =  4.50–7.59, respectively). **Conclusions**: Multimorbidity increasingly spans physical, psychological, and cognitive domains in both China and the US. Psychological conditions appeared to bridge physical and cognitive conditions in more complex multimorbidity patterns. Integrated care models incorporating psychological health and strategies addressing socioeconomic inequalities may help reduce the burden of multimorbidity and related functional decline.

## 1. Background

The increasing prevalence of chronic conditions has placed a significant burden on healthcare systems worldwide, particularly in the context of population aging [1]. Multimorbidity, commonly defined as the co-occurrence of two or more chronic conditions [2], has become a major public health challenge across diverse settings [2,3]. The global prevalence of physical multimorbidity is estimated to be approximately 37.2%, increasing markedly with age, affecting around 65% of individuals aged 65 to 84 years and over 80% among those aged 85 years and older [4]. However, the burden of conventional physical multimorbidity varies across countries [5]. In China, the prevalence among adults aged 45 years and older increased from 37.87% in 2011 to 61.14% in 2018 [6], whereas in the United States (US), it has remained relatively stable at approximately 43% [4].

In recent years, the concept of multimorbidity has expanded beyond physical conditions to include psychological and cognitive disorders [7,8]. Those conditions are often underdiagnosed, particularly in low- and middle-income settings, yet they play an important role in shaping overall disease burden. Emerging evidence suggests that physical, psychological, and cognitive conditions frequently cooccur and interact [9], giving rise to cross-domain multimorbidity. For example, depression and anxiety are common among individuals with chronic physical conditions such as diabetes [10], while cognitive impairment is frequently observed in patients with cardiovascular diseases [11,12]. Such cross-domain combinations are strongly associated with adverse health outcomes, including functional limitations in activities of daily living (ADL) and instrumental activities of daily living (IADL). Understanding how these conditions cluster across domains is therefore critical for improving prevention and management strategies.

Despite growing recognition of the complexity of multimorbidity, most existing studies have focused either on physical conditions alone or on cross-sectional descriptions of disease combinations [9]. Longitudinal evidence examining how multimorbidity domains evolve over time, particularly across physical, psychological, and cognitive dimensions, remains limited [13]. In addition, cross-national comparisons of multimorbidity patterns are scarce, even though countries differ substantially in socioeconomic conditions, healthcare systems, and lifestyle factors. These contextual differences may influence not only the distribution of multimorbidity but also its associated functional consequences.

To address these gaps, this study investigates the distribution and longitudinal changes in multimorbidity domains and their associations with socioeconomic status and healthcare factors in China and the US. Using harmonized data from two large national surveys across five waves, this study aims to characterize multimorbidity across physical, psychological, and cognitive domains, and to examine their relationships with functional limitations. By comparing these patterns across two distinct healthcare and social contexts, this study provides new insights into the determinants and consequences of multimorbidity.

## 2. Methods

### 2.1. Study Design and Participants

This cohort study used longitudinal data from the China Health and Retirement Longitudinal Study (CHARLS) and the Health and Retirement Study (HRS), two nationally representative surveys with broadly comparable designs for cross-national comparison [14,15]. Five survey waves were included to construct comparable follow-up periods (CHARLS, 2011–2020; HRS, 2012–2020). Harmonized datasets were used: for CHARLS, wave 5 variables were aligned with the harmonized definitions used in earlier waves; and for HRS, the RAND HRS Longitudinal File 2020 (V1) was used together with variables from the Harmonized HRS dataset [16]. Participants were required to have health information at baseline (wave 1) and the final follow-up (wave 5). Those with all health-related variables missing at either of these two waves were excluded. Missing observations in intermediate waves were permitted, but individuals with more than 30% missing values across all study variables and survey waves combined were excluded. To improve comparability in basic demographic structure, CHARLS and HRS participants were matched 1:1 by baseline age and sex. The primary analytic cohort therefore consisted of matched participants with available health information at both baseline and final follow-up. Further details and the cohort flowchart are provided in Supplementary Text S1 and Supplementary Figure S1.

### 2.2. Procedures and Outcomes

A total of 35 variables were extracted across five survey waves, including 16 contextual variables, 17 health-related variables, and two functional outcomes. Contextual variables covered demographic, lifestyle, socioeconomic, and healthcare-related factors; health-related variables included eight physical conditions, six psychological conditions, and three cognitive conditions. Detailed variable definitions are provided in Supplementary Table S1. Large-scale epidemiological studies rarely provide a single data source that fully captures the breadth of conditions required to assess multimorbidity longitudinally [13]. To preserve longitudinal comparability and sample size, variables unavailable in a given wave were interpolated from adjacent waves where appropriate, and individual-level missing values were imputed using random forest models evaluated by out-of-bag performance [17,18]. Additional details, including variable- and wave-specific native observation proportions and out-of-bag errors, are provided in Supplementary Text S2 and Tables S3 and S4.

For comparability across surveys, categorical variables with more than three levels were simplified into binary or ternary groups. Continuous variables, including household income and household wealth, were divided into country-specific tertiles to represent relative socioeconomic position and reduce the influence of skewed distributions and extreme values, following validated harmonization procedures in previous global health studies [1]. Medical expenditure was categorized as none, low, and high, because more than one third of participants reported no expenditure. All 17 health-related variables were dichotomized. Obesity was defined as a body mass index (BMI) of 30 or higher, and other physical conditions were based on self-reported physician diagnoses. Psychological conditions were assessed using harmonized CESD from CHARLS and HRS. Six items common to the CESD-10 and CESD-8 were retained, and symptoms were defined as present if reported for more than three days during the previous week. Cognitive conditions covered episodic memory disorder, working memory disorder, and orientation, using harmonized thresholds from test scores [19,20,21]. Detailed definitions are provided in the Supplementary Table S5 and Text S3.

The primary outcome was multimorbidity domains membership, classified into eight mutually exclusive categories: physical, psychological, cognitive, physical–psychological, physical–cognitive, psychological–cognitive, physical–psychological–cognitive, and non-multimorbidity. In line with the conventional definition of multimorbidity, participants with zero or one condition were classified as non-multimorbid (Supplementary Table S6). Secondary outcomes were limitations in ADL and IADL limitations (Supplementary Table S5). Mortality was not analyzed because death information was limited and could not be reliably distinguished from other forms of attrition among participants without final-wave health information in a comparable way across cohorts.

### 2.3. Statistical Analysis

Multimorbidity networks were constructed to examine the co-occurrence among physical, psychological, and cognitive conditions. Nodes represented individual conditions and edges represented pairwise associations estimated using logistic regression. Networks were constructed separately by country, sex, and survey wave. Edge was retained according to prespecified criteria based on the odds ratio (OR), multiple-testing correction, and condition prevalence [5,22]. In the network analysis, core nodes were defined as degree ≥ 10 to represent highly connected conditions within the 17-condition network. Sensitivity of network structure was evaluated using alternative edge-retention criteria (e.g., varying OR thresholds), which yielded consistent core-node patterns and network topology (Supplementary Text S4). Changes in multimorbidity domain membership over time were visualized using Sankey diagrams.

Multinomial logistic mixed models were fitted to examine the associations of socioeconomic status and healthcare factors with repeated multimorbidity domain membership over time, with non-multimorbidity as the reference category. Individual-level random intercepts accounted for repeated observations, and linear and quadratic time terms captured nonlinear temporal trends. Subgroup analyses were conducted by selected sociodemographic and lifestyle characteristics. Cox proportional hazard models were used to estimate hazard ratios (HRs) and 95% confidence intervals (CIs) for associations between time-varying multimorbidity domains and ADL and IADL limitations during follow-up. For each outcome-specific model, participants with the corresponding limitation at baseline were excluded. Person-time was calculated from baseline to the first reported ADL or IADL difficulty limitation or the end of observation. Proportional hazards assumptions were evaluated using Schoenfeld residuals and showed no major violations. Survey weights were not incorporated in the primary matched-cohort analyses to preserve the matched longitudinal design, as the study focused on association estimates within the matched sample rather than population-level prevalence. Sensitivity analyses were conducted using the natural cohort before 1:1 age- and sex-matching and before construction of the final matched analytic cohort. These analyses included baseline comparisons, multinomial logistic mixed models, and Cox models for subsequent ADL and IADL limitations, to assess whether the main findings were sensitive to sample restriction, matching, and imputation-related analytic decisions. All statistical analyses were conducted using R software (version 4.2.1).

### 2.4. Role of the Funding Source

The funders had no role in the study design, data collection, analysis, interpretation, manuscript preparation, or the decision to submit the manuscript.

## 3. Results

A total of 7064 participants from China and 7064 age- and sex-matched participants from the US were included in the final analytical sample (Supplementary Figure S1). Before matching, participants in both the natural and complete US cohorts were older than those in China (Supplementary Table S7). After matching, the two cohorts had identical age and sex distributions (mean age 60.15 ± 7.93 years, 45.4% male). Differences between countries remained in most socioeconomic, behavioral, and healthcare-related characteristics (Table 1).

**Figure 1:**
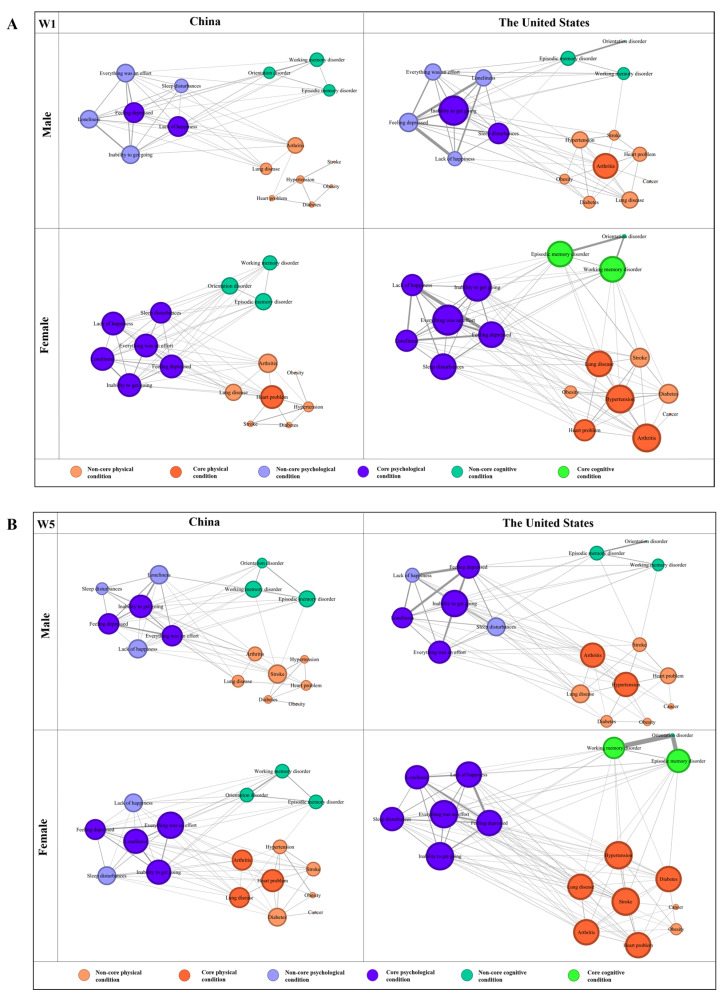
Multimorbidity network diagrams visualizing the various condition pairs that existed in the (**A**) first wave, and (**B**) last wave. W1: wave1, W5: wave5.

The multimorbidity network illustrated co-occurrence patterns among physical, psychological, and cognitive conditions (Figure 1 and Supplementary Figures S2–S4). At baseline, under the prespecified edge-retention criteria, networks were more complex in the US than in China, with more retained edges in both males (61 vs. 50) and females (85 vs. 64), while the number of nodes was similar. Across subsequent waves, connectivity generally decreased (Supplementary Table S8). The strongest condition pairs at baseline were mainly within psychological conditions in both countries. In China, the strongest associations were between loneliness and inability to get going (OR = 9.19 in males; OR = 8.57 in females) (Figure 1A). These patterns remained largely consistent across waves. In US females, several strong late-wave connections were observed among cognitive conditions, including working memory disorder with orientation disorder and episodic memory disorder with orientation disorder (Figure 1B). These large estimates should be interpreted cautiously because of sparse cells. Psychological conditions occupied central positions in the networks. Most psychological nodes had a degree ≥ 7, and 33.3% to 100.0% were core nodes (degree ≥ 10) (Figure 1 and Supplementary Figures S2–S4), indicating a high level of connectivity with other conditions. When psychological conditions were removed, physical and cognitive networks in China became disconnected during the early waves, whereas limited links remained in the US, with stroke serving as a bridging node among males (Supplementary Figures S5–S7, Supplementary Tables S9–S11).

The distribution and progression of multimorbidity domains differed between the two countries (Supplementary Figure S8). In China, physical–psychological–cognitive multimorbidity was the dominant domain at baseline and increased steadily over time (from 2089 to 3249). This increase was mainly driven by transitions from physical–psychological (transition probability: 32.0–35.7%) and physical–cognitive (transition probability: 36.7–44.9%) multimorbidity (Supplementary Table S12). Non-multimorbidity and psychological–cognitive multimorbidity were common at baseline but declined over time, with individuals frequently transitioning from non-multimorbidity to psychological–cognitive multimorbidity (transition probability: 10.9–15.5%), and subsequently to physical–psychological–cognitive domain multimorbidity (transition probability: 8.9–28.2%) (Supplementary Figure S8a). In the US, physical–psychological multimorbidity was the most prevalent domain at baseline and continued to increase over time (from 1773 to 2076), largely driven by transitions from physical multimorbidity (transition probability: 20.3–28.0%) (Supplementary Table S13). Similar to China, non-multimorbidity decreased across waves (from 1612 to 1048), with individuals transitioning to multimorbidity domains, particularly physical–psychological multimorbidity (transition probability: 10.1–13.9%). Physical multimorbidity also increased over time (from 1316 to 1676), while physical–psychological–cognitive multimorbidity remained relatively stable (from 1297 to 1336). Other multimorbidity domains were less common and generally declined in both countries (Supplementary Figure S8b). In general, psychological conditions were frequently involved in transitions toward more complex cross-domain multimorbidity, with China showing more marked movement toward three-domain multimorbidity and the US showing a more stable predominance of physical–psychological multimorbidity.

**Figure 2:**
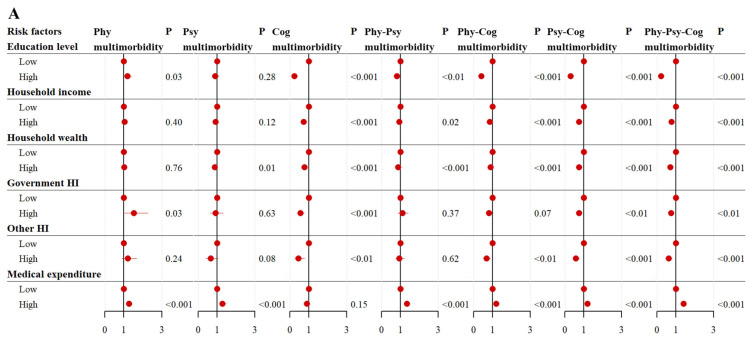

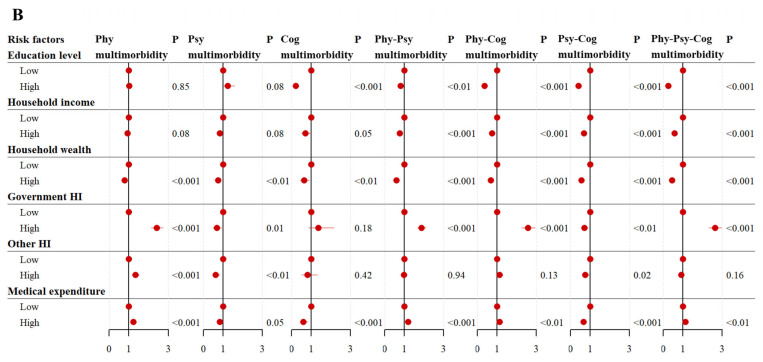
Levels of risk factors in relation to seven multimorbidity domains in (**A**) China, and (**B**) the United States. Red circles represent odds ratios (ORs), and horizontal lines represent 95% confidence intervals (CIs). Phy: physical; Psy: psychological; Cog: cognitive; HI: healthcare insurance.

Associations of socioeconomic status and healthcare factors with multimorbidity domains are presented in Figure 2. Across both countries, higher socioeconomic status was consistently associated with lower odds of multimorbidity, particularly for cross-domain conditions. Higher education was associated with lower odds of physical–psychological–cognitive multimorbidity (China: OR = 0.22, 95% CI [0.19–0.26]; US: OR = 0.26, 95% CI [0.23–0.30]) and cognitive multimorbidity (China: OR = 0.24 [0.17–0.34]; US: OR = 0.22 [0.15–0.33]), although in China it was modestly associated with higher odds of physical multimorbidity (OR = 1.19 [1.02–1.39]). Household wealth showed a more consistent protective association across most multimorbidity domains. The inverse association was most evident for physical–psychological–cognitive multimorbidity (China: OR = 0.70 [95% CI: 0.66–0.74]; US: OR = 0.46 [95% CI: 0.42–0.50]), as well as for psychological–cognitive multimorbidity (China: OR = 0.74 [95% CI: 0.70–0.79]; US: OR = 0.55 [95% CI: 0.47–0.65]) and cognitive multimorbidity (China: OR = 0.77 [95% CI: 0.69–0.85]; US: OR = 0.64 [95% CI: 0.46–0.89]). Compared with household wealth, household income showed less consistent associations across domains.

Associations related to healthcare factors differed between the two countries (Figure 2). In China, government health insurance was associated with lower odds of cognitive multimorbidity (OR = 0.56 [95% CI: 0.41–0.75]), psychological–cognitive multimorbidity (OR = 0.74 [95% CI: 0.60–0.90]), and physical–psychological–cognitive multimorbidity (OR = 0.74 [95% CI: 0.62–0.90]), but higher odds of physical multimorbidity (OR = 1.53 [95% CI: 1.03–2.26]). In the US, government health insurance was associated with higher risks of physical-related multimorbidity, including physical multimorbidity (OR = 2.41 [95% CI: 2.14–2.72]), physical–psychological multimorbidity (OR = 1.88 [95% CI: 1.67–2.11]), physical–cognitive multimorbidity (OR = 2.57 [95% CI: 2.25–2.93]), and physical–psychological–cognitive multimorbidity (OR = 2.63 [95% CI: 2.31–2.98]), while lower risks were observed for psychological multimorbidity (OR = 0.68 [95% CI: 0.50–0.92]) and psychological–cognitive multimorbidity (OR = 0.70 [95% CI: 0.55–0.90]). For other types of health insurance, lower risk of psychological (OR = 0.63 [95% CI: 0.47–0.84]) and psychological-cognitive multimorbidity (OR = 0.75 [95% CI: 0.59–0.96]) were observed in the US, whereas in China, protective associations were mainly observed for cognitive-related multimorbidity domains.

Higher medical expenditure was associated with increased risks of multimorbidity in both countries, although the pattern differed. In China, higher expenditure was linked to increased risks across nearly all domains. In the US, the association was primarily observed in physical-related multimorbidity, including physical multimorbidity (OR = 1.22 [95% CI: 1.14–1.30]), physical–psychological multimorbidity (OR = 1.20 [95% CI: 1.12–1.28]), and physical–cognitive multimorbidity (OR = 1.12 [95% CI: 1.04–1.20]).

Subgroup analyses showed that the protective association of higher education with cognitive and cross-domain multimorbidity was generally consistent across demographic and behavioral groups (Supplementary Text S5 and Tables S14–S21). Some heterogeneity was observed in specific subgroups, particularly for physical multimorbidity in China and psychological multimorbidity in the US.

**Figure 3:**
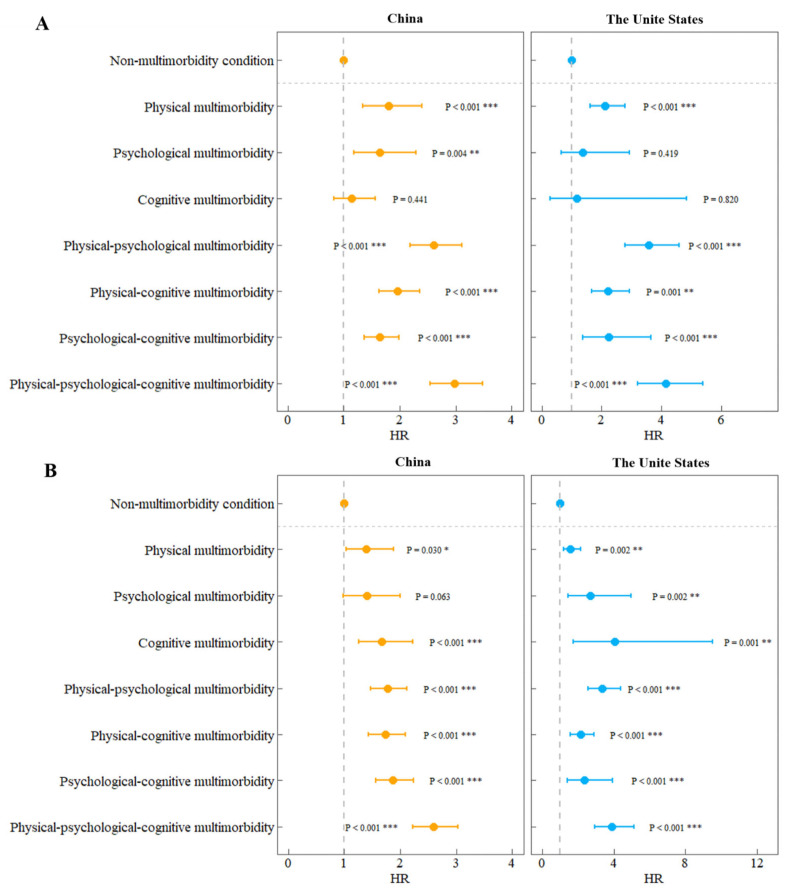
Forest diagrams of the levels of multimorbidity categories in relation to ADL (**A**) and IADL (**B**). * *p* ≤ 0.05; ** *p* ≤ 0.01; *** *p* ≤ 0.001.

Multimorbidity domains remain strongly associated with functional limitations during follow-up after adjustment for sociodemographic, behavioral, socioeconomic, and healthcare-related factors in both countries (Figure 3). Cross-domain multimorbidity, especially combinations involving physical, psychological, and cognitive conditions, was associated with higher hazards of ADL and IADL limitations than within-domain multimorbidity. For ADL limitations, cross-domain multimorbidity showed stronger associations than within-domain multimorbidity. The strongest associations were observed for physical–psychological–cognitive multimorbidity, with adjusted hazard ratios of 2.97 (95% CI: 2.54–3.48) in China and 4.51 (95% CI: 3.21–5.37) in the US. Physical–psychological multimorbidity also showed strong associations in both countries (China: HR = 2.60 [95% CI: 2.18–3.11]; US: HR = 3.57 [95% CI: 2.78–4.58]). Overall, adjusted HRs for ADL limitations tended to be higher in the US than in China across most multimorbidity domains (Supplementary Table S22). Similar patterns were observed for IADL limitations. Physical–psychological–cognitive multimorbidity was again associated with the highest hazard, with adjusted hazard ratios of 2.60 (95% CI: 2.23–3.03) in China and 3.89 (95% CI: 2.93–5.19) in the US. Cross-domain multimorbidity domains were consistently associated with higher hazards of IADL limitations than most within-domain conditions, although the magnitude of associations was generally smaller than that observed for ADL limitations (Supplementary Table S23).

In sensitivity analyses using the natural cohort before 1:1 age- and sex-matching and before construction of the final matched analytic cohort, baseline comparisons showed that matching mainly aligned age and sex distributions, while cross-country differences in other variables remained evident (Supplementary Table S7). The associations of socioeconomic and healthcare-related factors with multimorbidity domains, as well as the associations between multimorbidity domains and subsequent ADL and IADL limitations, were broadly consistent with those observed in the primary analytic cohort (Supplementary Tables S24–S27).

## 4. Discussion

This study provides longitudinal evidence on the development and consequences of multimorbidity across physical, psychological, and cognitive domains in China and the US. Three key findings emerged. First, psychological conditions occupied a central position in multimorbidity networks and were closely linked to transitions toward more complex cross-domain patterns over time. Second, although the overall direction of these associations was comparable between China and the US, the dominant multimorbidity patterns and their trajectories differed between the two settings, suggesting that multimorbidity trajectories may vary across social and healthcare settings. Third, cross-domain multimorbidity, particularly the co-occurrence of physical, psychological, and cognitive conditions, was consistently associated with the highest risk of functional limitations in both countries.

A notable finding of this study is the central and bridging role of psychological conditions in the multimorbidity network. While previous studies have often treated psychological disorders as a consequence of physical or cognitive decline [23,24], our longitudinal results suggest psychological conditions were persistently connected with both physical and cognitive conditions, and removal of psychological nodes disconnected physical and cognitive networks during the early waves, particularly in China. These findings suggest that psychological conditions may occupy a key connecting position in the formation of cross-domain multimorbidity patterns. This is consistent with evidence indicating bidirectional relationships between mental and physical health, including shared inflammatory pathways, vascular mechanisms, and behavioral factors that jointly contribute to disease progression [25,26,27]. Psychological disorders may therefore be more than downstream manifestations of illness burden; they may mark an important connecting point through which physical, psychological, and cognitive conditions become increasingly interconnected.

Although similar overall patterns were observed in China and the US, their multimorbidity trajectories differed. At baseline, multimorbidity networks were more densely connected in the US, whereas China showed more marked transitions toward complex cross-domain multimorbidity over time. These differences are unlikely to reflect biology alone. They may also capture variation in disease detection, healthcare access, continuity of care, and population health profiles [28,29]. Socioeconomic factors, particularly education and household wealth, were consistently associated with lower odds of cognitive-related and cross-domain multimorbidity in both countries, reflecting differences in accumulated social and material resources, health literacy, living conditions, and access to supportive care [27,30]. Healthcare-related variables showed mixed associations across domains and settings. Insurance type and medical expenditure may reflect healthcare access and utilization, as well as underlying healthcare needs and disease severity [31,32]. In particular, government health insurance has different coverage levels and eligibility structures in China and the US, so the corresponding coefficients should be interpreted within each country’s healthcare context. The subgroup analyses add some detail to this picture, but they do not change the overall pattern; additional interpretation is provided in Supplementary Text S6. Taken together, our findings are better understood as indicating that the social patterning of multimorbidity remains uneven, and that this unevenness becomes more visible as multimorbidity extends across cognitive, psychological, and physical domains.

Our findings also indicate that the consequences of multimorbidity may depend not only on the number of conditions but also on how they cluster across domains. Compared with within-domain multimorbidity, cross-domain multimorbidity was more strongly associated with ADL and IADL difficulties during follow-up, even after adjustment for sociodemographic, behavioral, socioeconomic, and healthcare-related factors. The stronger associations observed for ADL than for IADL limitations also deserve note; additional interpretation is provided in Supplementary Text S7. This supports the view that multimorbidity across physical, psychological, and cognitive domains may generate synergistic effects that accelerate functional deterioration beyond a simple additive burden of diseases [33,34]. Physical limitations may reduce mobility and social engagement, psychological symptoms may impair motivation and treatment adherence, and cognitive impairment may compromise self-management. When these deficits co-occur, their combined effects are likely to intensify disability risk. In this sense, cross-domain multimorbidity may be understood as a marker of systemic vulnerability rather than simply a higher disease count.

These findings have several practical implications for multimorbidity prevention and management. Risk assessment should move beyond simple disease counts, because individuals with cross-domain multimorbidity appear to face substantially greater risks of functional decline than those whose conditions remain within a single domain [35]. Greater attention may therefore be needed for people with combined physical, psychological, and cognitive problems, especially in primary care and community settings. Screening for psychological symptoms and cognitive vulnerability may be particularly useful among socioeconomically disadvantaged groups, who were consistently more likely to develop complex cross-domain multimorbidity across both cohorts. The central position of psychological conditions also suggests that earlier identification of psychological symptoms, cognitive vulnerability, and related social disadvantage may help delay progression toward more complex multimorbidity [23,36,37]. At the same time, the cross-national differences observed here indicate that priorities may differ by setting: in China, reducing socioeconomic and healthcare inequalities may be particularly important, whereas in the US greater emphasis may be needed on managing the functional burden of already established complex multimorbidity [38,39,40].

Several limitations should be noted. First, some health conditions and healthcare-related variables in both CHARLS and HRS were self-reported, and only those that could be harmonized across cohorts were analyzed. This may have introduced cross-country reporting differences and underestimated the true burden of multimorbidity. Second, the analytical sample was restricted to participants with baseline and final-wave data, and mortality was not consistently captured across waves. As a result, the study may represent a healthier surviving cohort, potentially underestimating progression toward severe or complex multimorbidity [41]. However, sensitivity analyses using a broader natural cohort yielded consistent findings. Third, missing data in intermediate waves were handled using adjacent-wave interpolation and random-forest imputation to preserve longitudinal comparability and sample size. However, this single-imputation approach did not fully propagate imputation uncertainty into the regression estimates. Although sensitivity analyses using the natural cohort yielded broadly consistent findings, domain-specific and subgroup results should still be interpreted cautiously. Fourth, our models did not fully assess the indirect pathways among socioeconomic factors, healthcare access, psychological conditions, multimorbidity progression, and functional decline. Future studies using structural equation modeling, mediation analysis, or intervention designs may help clarify these pathways. Fifth, the matching between the two cohorts was conducted primarily based on baseline age and sex. Although this effectively balanced key demographic structures, it may not completely eliminate unmeasured residual confounding or differences in other baseline healthcare characteristics between the cohorts. Despite these limitations, this study provides a rare longitudinal comparison of multimorbidity trajectories across physical, psychological, and cognitive domains in large aging cohorts from China and the US.

## 5. Conclusions

In both China and the US, multimorbidity in middle-aged and older adults increasingly extended across physical, psychological, and cognitive domains over time. Psychological conditions occupied a central position in these trajectories and appeared to bridge physical and cognitive domains in complex multimorbidity patterns, particularly in China. Cross-domain multimorbidity, especially physical–psychological–cognitive multimorbidity, was associated with the highest risk of subsequent functional limitations in both countries. Although multimorbidity patterns were generally more complex in the US, transitions towards complex multimorbidity were more marked in China. Socioeconomic disadvantages were consistently associated with more complex multimorbidity patterns, while healthcare-related factors showed setting-specific associations. Therefore, future multimorbidity assessment should move beyond disease counts and pay greater attention to cross-domain combinations, especially those involving psychological and cognitive conditions. Earlier identification of individuals with complex cross-domain multimorbidity may help target prevention and management strategies for functional decline in aging populations.

## Figures and Tables

**Table 1:** Baseline demographic characteristics and behavioral lifestyle of CHARLS and HRS participants.

Characteristic	China	The United States	*p*-Value
*N*	7064	7064	
Demographics			
Sex			1.000
Male	3208 (45.4%)	3208 (45.4%)	
Female	3856 (54.6%)	3856 (54.6%)	
Age (Mean ± SD *, years)	60.15 ± 7.93	60.15 ± 7.93	1.000
Area			<0.001
Urban	2752 (39.0%)	5331 (75.5%)	
Rural	4312 (61.0%)	1733 (24.5%)	
Household members (Mean ± SD *)	3.55 ± 1.86	2.57 ± 1.40	<0.001
Marital status			<0.001
Unmarried	1227 (17.4%)	2473 (35.0%)	
Married	5837 (82.6%)	4591 (65.0%)	
Lifestyle			
BMI (Mean ± SD *, Kg/m^2^)	23.64 ± 3.69	29.34 ± 6.08	<0.001
Drinking			<0.001
No	6059 (85.8%)	5796 (82.0%)	
Yes	1005 (14.2%)	1268 (18.0%)	
Smoking			<0.001
No	5033 (71.3%)	5981 (84.7%)	
Yes	2031 (28.7%)	1083 (15.3%)	
Physical Activity			<0.001
No	2168 (30.7%)	1672 (23.7%)	
Yes	4896 (69.3%)	5392 (76.3%)	
Socioeconomic status			
Education			<0.001
Less than lower secondary	6287 (89.0%)	1034 (14.6%)	
Upper secondary	675 (9.6%)	4113 (58.2%)	
Tertiary	102 (1.4%)	1917 (27.2%)	
Household income (country-specific tertiles) *			1.000
Low	2355 (33.3%)	2355 (33.3%)	
Middle	2354 (33.3%)	2354 (33.3%)	
High	2355 (33.3%)	2355 (33.3%)	
Household wealth (country-specific tertiles) *			
Low	2355 (33.3%)	2365 (33.5%)	0.981
Middle	2354 (33.3%)	2345 (33.2%)	
High	2355 (33.3%)	2354 (33.3%)	
Healthcare			
Government health insurance			<0.001
No	494 (7.0%)	4416 (62.5%)	
Yes	6570 (93.0%)	2648 (37.5%)	
Other health insurance			<0.001
No	6856 (97.0%)	1963 (27.8%)	
Yes	208 (3.0%)	5101 (72.2%)	
Medical expenditure			<0.001
None	6461 (91.4%)	948 (13.4%)	
Low	310 (4.4%)	3039 (43.0%)	
High	293 (4.1%)	3077 (43.6%)	

* SD: standard deviation. *p*-values for continuous variables were calculated using *t*-test; *p*-values for categorical variables were calculated using χ^2^ test. Household income and household wealth were categorized into country-specific tertiles (Low, Middle, and High, each representing approximately 33.3% of the cohort) to account for cross-national economic disparities.

## Data Availability

The original data for this study are available on their respective websites: The China Health and Retirement Longitudinal Study (CHARLS; http://charls.pku.edu.cn (accessed on 12 July 2023)) and The Health and Retirement Study (HRS; https://hrs.isr.umich.edu (accessed on 6 September 2023)).

## Supplementary Materials

The following supporting information can be downloaded at: https://www.mdpi.com/article/10.3390/healthcare14152326/s1, Text S1. Harmonization of data sources and cohort construction. Text S2. Rationale and implementation of the two-stage missing-data strategy. Text S3. Operational definitions of psychological and cognitive conditions. Text S4. Additional details of network construction. Text S5. Subgroup analysis for factors associated with multimorbidity domains and functional outcomes. Text S6. Additional interpretation of subgroup findings. Text S7. Additional interpretation of functional findings. Table S1. Variable collection. Table S2. Imputation of missing variables based on adjacent wave. Table S3. Evaluation of out-of-bag error for imputation of missing values using random forest model based on non-missing values in CHARLS database. Table S4. Evaluation of out-of-bag error for imputation of missing values using random forest model based on non-missing values in HRS database. Table S5. Harmonized strategies for key variables included in present study. Table S6. Definition of eight multimorbidity categories. Table S7. Comparison of the baseline demographic characteristics and behavioral lifestyle among natural cohort, complete cohort before matching, and complete cohort after matching. Table S8. Number of nodes, edges, and frequency of conditions pairs in the multimorbidity networks. Table S9. Number of nodes, edges, and frequency of conditions pairs in the psychological-cognitive multimorbidity networks. Table S10. Number of nodes, edges, and frequency of conditions pairs in the physical-cognitive multimorbidity networks. Table S11. Number of nodes, edges, and frequency of conditions pairs in the physical -psychological multimorbidity networks. Table S12. Transition probability of multimorbidity for China participants. Table S13. Transition probability of multimorbidity for US participants. Table S14. Sex-stratified analysis of socioeconomic and health factors associated with multimorbidity outcomes. Table S15. Age-stratified analysis of socioeconomic and health factors associated with multimorbidity outcomes. Table S16. Whether lived in rural—stratified analysis of socioeconomic and health factors associated with multimorbidity outcomes. Table S17. Household members level—stratified analysis of socioeconomic and health factors associated with multimorbidity outcomes. Table S18. Marital status—stratified analysis of socioeconomic and health factors associated with multimorbidity outcomes. Table S19. Whether Drinking—stratified analysis of socioeconomic and health factors associated with multimorbidity outcomes. Table S20. Whether smoking—stratified analysis of socioeconomic and health factors associated with multimorbidity outcomes. Table S21. Frequency level of activity—stratified analysis of socioeconomic and health factors associated with multimorbidity outcomes. Table S22. Adjusted associations of multimorbidity patterns with ADL limitations in China and the US. Table S23. Adjusted associations of multimorbidity patterns with IADL limitations in China and the US. Table S24. Associations of socioeconomic factors and health factors with multimorbidity patterns in China based on natural cohort. Table S25. Associations of socioeconomic factors and health factors with multimorbidity patterns in the US based on natural cohort. Table S26. Adjusted associations of multimorbidity patterns with ADL limitations in China and the US based on natural cohort. Table S27. Adjusted associations of multimorbidity patterns with IADL limitations in China and the US based on natural cohort. Figure S1. Flowchart showing the selection of participants. Figure S2. Multimorbidity network diagrams visualizing the various condition pairs that existed in wave 2. Figure S3. Multimorbidity network diagrams visualizing the various condition pairs that existed in wave 3. Figure S4. Multimorbidity network diagrams visualizing the various condition pairs that existed in wave 4. Figure S5. Multimorbidity network diagrams visualizing the various psychological and cognitive condition pairs that existed in each wave. Figure S6. Multimorbidity network diagrams visualizing the various physical and cognitive condition pairs that existed in each wave. Figure S7. Multimorbidity network diagrams visualizing the various psychological and cognitive condition pairs that existed in each wave. Figure S8. Sankey diagrams visualizing the evolution of the multimorbidity categories across five waves. References [42,43] are included in the Supplementary Materials.

## Abbreviations

US	United States
HR	hazard ratio
CES-D	Center for Epidemiological Depression
ADL	activities of daily living
IADL	instrumental activities of daily living
CHARLS	China Health and Retirement Longitudinal Study
HRS	Health and Retirement Study
OR	odds ratio
CI	confidence interval
