# Supplementary material for: Longitudinal Patterns of Within- and Cross-Domain Multimorbidity Across Physical, Psychological, and Cognitive Conditions in China and the United States: The Role of Socioeconomic and Healthcare Inequalities
Source: Healthcare (Basel). 2026 Aug 1;14(15):2326. doi: 10.3390/healthcare14152326 (PMC13464807; doi:10.3390/healthcare14152326)
Supplement: Supplementary file 1 [file healthcare-14-02326-s001.zip › healthcare-4388925-supplementary.pdf]

Supplementary Materials

Supplementary Texts

Text S1. Harmonization of data sources and cohort construction
Text S2. Rationale and implementation of the two-stage missing-data strategy
Text S3. Operational definitions of psychological and cognitive conditions
Text S4. Additional details of network construction
Text S5. Subgroup analysis for factors associated with multimorbidity domains and functional outcomes
Text S6. Additional interpretation of subgroup findings
Text S7. Additional interpretation of functional findings

Supplementary Tables

Table S1. Variable collection
Table S2. Imputation of missing variables based on adjacent wave
Table S3. Evaluation of out-of-bag error for imputation of missing values using random forest model based on non-missing values in CHARLS database
Table S4. Evaluation of out-of-bag error for imputation of missing values using random forest model based on non-missing values in HRS database
Table S5. Harmonized strategies for key variables included in present study
Table S6. Definition of eight multimorbidity categories
Table S7. Comparison of the baseline demographic characteristics and behavioral lifestyle among natural cohort, complete cohort before matching, and complete cohort after matching
Table S8. Number of nodes, edges, and frequency of conditions pairs in the multimorbidity networks
Table S9. Number of nodes, edges, and frequency of conditions pairs in the psychological-cognitive multimorbidity networks
Table S10. Number of nodes, edges, and frequency of conditions pairs in the physical-cognitive multimorbidity networks
Table S11. Number of nodes, edges, and frequency of conditions pairs in the physical -psychological multimorbidity networks
Table S12. Transition probability of multimorbidity for China participants
Table S13. Transition probability of multimorbidity for US participants
Table S14. Sex-stratified analysis of socioeconomic and health factors associated with multimorbidity outcomes
Table S15. Age-stratified analysis of socioeconomic and health factors associated with multimorbidity outcomes
Table S16. Whether lived in rural—stratified analysis of socioeconomic and health factors associated with multimorbidity outcomes
Table S17. Household members level—stratified analysis of socioeconomic and health

factors associated with multimorbidity outcomes
Table S18. Marital status—stratified analysis of socioeconomic and health factors associated with multimorbidity outcomes
Table S19. Whether Drinking—stratified analysis of socioeconomic and health factors associated with multimorbidity outcomes
Table S20. Whether smoking—stratified analysis of socioeconomic and health factors associated with multimorbidity outcomes
Table S21. Frequency level of activity—stratified analysis of socioeconomic and health factors associated with multimorbidity outcomes
Table S22. Adjusted associations of multimorbidity patterns with ADL limitations in China and the US
Table S23. Adjusted associations of multimorbidity patterns with IADL limitations in China and the US
Table S24. Associations of socioeconomic factors and health factors with multimorbidity patterns in China based on natural cohort
Table S25. Associations of socioeconomic factors and health factors with multimorbidity patterns in the US based on natural cohort
Table S26. Adjusted associations of multimorbidity patterns with ADL limitations in China and the US based on natural cohort
Table S27. Adjusted associations of multimorbidity patterns with IADL limitations in China and the US based on natural cohort

Supplementary Figures

Figure S1. Flowchart showing the selection of participants
Figure S2. Multimorbidity network diagrams visualizing the various condition pairs that existed in wave 2
Figure S3. Multimorbidity network diagrams visualizing the various condition pairs that existed in wave 3
Figure S4. Multimorbidity network diagrams visualizing the various condition pairs that existed in wave 4
Figure S5. Multimorbidity network diagrams visualizing the various psychological and cognitive condition pairs that existed in each wave
Figure S6. Multimorbidity network diagrams visualizing the various physical and cognitive condition pairs that existed in each wave
Figure S7. Multimorbidity network diagrams visualizing the various psychological and cognitive condition pairs that existed in each wave
Figure S8. Sankey diagrams visualizing the evolution of the multimorbidity categories across five waves

Supplementary Texts

Text S1. Harmonization of data sources and cohort construction

The primary data sources were the Harmonized CHARLS dataset and the RAND HRS Longitudinal File 2020, used together with variables from the Harmonized HRS dataset [1, 2]. Because CHARLS wave 5 had not yet been incorporated into the harmonized release at the time of analysis, wave 5 variables were manually aligned with the harmonized definitions used in earlier waves. This alignment was performed variable by variable to preserve consistency in coding and interpretation across the five-wave follow-up. The primary analytic cohort required health information at both the baseline and final waves. This design ensured that longitudinal multimorbidity trajectories could be reconstructed over a comparable observation window in both countries. Intermediate-wave non-response was not treated as an exclusion criterion because attrition at a single wave may reflect survey participation patterns rather than a complete absence of health information. To assess the robustness of this restriction, sensitivity analyses were additionally conducted in a natural cohort that included all baseline participants regardless of wave 5 participation.

Text S2. Rationale and implementation of the two-stage missing-data strategy

In repeated population surveys, two common challenges arise: some variables may be entirely unavailable in new cross-sectional surveys because of changes in data collection, and missing or implausible values may occur at the individual level during data collection. Restricting analyses to complete cases or dropping variables would reduce both the number of available indicators and the analytic sample, limiting the ability to characterize the progression of multimorbidity across domains. The two-stage missing-data strategy was adopted to address two distinct forms of incompleteness that arise in repeated population surveys [3]. The first occurs at the wave level, when a variable is entirely unavailable in one survey cycle because of changes in questionnaire content or delays in harmonization. The second occurs at the individual level, when responses are missing or implausible within an otherwise observed wave. These two forms of missingness were handled separately because they reflect different problems. When an entire variable was absent in a given wave, adjacent-wave interpolation was used, where appropriate, to preserve longitudinal comparability rather than treating the entire wave as structurally missing (Supplementary Table S2). When values were missing at the participant level, random forest imputation was then applied using the observed information from other variables and waves [4, 5]. This approach allowed retention of both the breadth of health indicators and the sample size, whereas dropping variables or restricting the analysis to complete cases would have substantially reduced the capacity to characterize multimorbidity progression. Variable- and wave-specific native observation proportions and out-of-bag errors are shown in Tables S3–S4.

Text S3. Operational definitions of psychological and cognitive conditions

Psychological conditions were harmonized between CHARLS and HRS by restricting the analysis to six items common to the CESD-10 and CESD-8. A symptom was coded as present when it was reported for more than three days in the previous week. This approach prioritized cross-national comparability over the use of instrument-specific summary scores. Cognitive conditions were assessed in three domains: episodic memory, working memory, and orientation [6-8]. Episodic memory was measured using immediate and delayed recall of 10 words; working memory was assessed using the Serial Sevens task; and orientation was based on calendar-date items. For each domain, impairment was defined as a score no higher than one third of the

maximum possible score. This threshold corresponds to a global cognitive score of 10 or below across the three domains. Detailed coding rules are shown in Table S5.

Text S4. Additional details of network construction

For network construction, pairwise logistic regression models were fitted for each condition pair within each country-, sex-, and wave-specific stratum. Edges were retained when all three prespecified criteria were met: odds ratio >1.2, Bonferroni-corrected P-value <0.05/N, and prevalence >1 per 10,000 participants [9, 10]. Degree was used to identify highly connected nodes, with degree ≥10 considered indicative of a core node. Network processing was performed in R version 4.2.1; visualization was undertaken in Gephi, and clustering analyses of selected node subsets were performed in Cystoscope. In the figures, edge thickness represents the magnitude of the odds ratio, node color indicates condition category, and node size reflects node degree.

Text S5. Subgroup analysis for factors associated with multimorbidity domains and functional outcomes

Within each subgroup defined by demographic characteristics and behavioral habits, higher education levels consistently demonstrated a protective effect against cognitive and cross-domain multimorbidities (P ≤ 0.05) (Supplementary Tables S14-S21). However, the association between higher education and increased risk of physical multimorbidity was observed exclusively in certain subgroups in China, such as males (OR = 1.22 [95% CI: 1.00–1.48]) (Supplementary Table S14), individuals aged over 60 years (OR = 1.35 [95% CI: 1.13–1.62]) (Supplementary Table S15), those with more household members (OR = 1.27 [95% CI: 1.04–1.55]) (Supplementary Table S17), married individuals (OR = 1.20 [95% CI: 1.02–1.41]) (Supplementary Table S18), non-drinkers (OR = 1.37 [95% CI: 1.12–1.68]) (Supplementary Table S19), and individuals with low physical activity levels (OR = 1.31 [95% CI: 1.06–1.63]) (Supplementary Table S21). In contrast, the increased risk of psychological multimorbidity associated with higher education levels was noted only among married individuals (OR = 1.39 [95% CI: 1.04–1.88]) (Supplementary Table S18), and non-drinkers (OR = 1.35 [95% CI: 1.03–1.78]) in the US (Supplementary Table S19). In comparison to higher household income, the protective effect of higher household wealth on physical multimorbidity (observed only in the US) and cross-domain multimorbidities was more consistently observed (P ≤ 0.05). Furthermore, higher household wealth was associated with a protective effect against psychological multimorbidity in both countries, particularly among married individuals, non-drinkers, and smokers (P ≤ 0.05) (Supplementary Tables S18-S20).

In the US, individuals with government health insurance and other health insurance demonstrated consistently higher risks of physical-related multimorbidities across various subgroups (P ≤ 0.05). However, several subgroups exhibited heterogeneity in relation to psychological and psychological-cognitive multimorbidity. For instance, individuals with government health insurance residing in non-rural areas (OR = 0.71 [95% CI: 0.51–0.99]) (Supplementary Table S16), living in households with more members (OR = 0.67 [95% CI: 0.50–0.89]), being married (OR = 0.57 [95% CI: 0.38–0.84]) (Supplementary Table S17), consuming alcohol (OR = 0.45 [95% CI: 0.21–0.97]) (Supplementary Table S19), being non-

smokers (OR = 0.65 [95% CI: 0.46–0.90]) (Supplementary Table S20), and engaging in higher levels of physical activity (OR = 0.56 [95% CI: 0.39–0.79]) (Supplementary Table S21) showed a lower risk of psychological multimorbidity, which was also observed in psychological–cognitive multimorbidity. In China, the association between government medical insurance and a reduced risk of cognitive-related diseases exhibited subgroup-specific variations. The protective effect was predominantly observed among females, individuals aged ≥60 years, those residing in non-rural areas, individuals with fewer household members, married individuals, non-drinkers, non-smokers, and those engaging in higher levels of physical activity ($P \leq 0.05$) (Supplementary Tables S14-S21). Regarding medical expenditure, a consistent pattern was observed across all multimorbidity domains in different subgroups in China. In the US, consistency was found in physical, physical–psychological, and psychological–cognitive multimorbidities, whereas heterogeneity was observed in other multimorbidity domains. For instance, the higher risk of physical-psychological–cognitive multimorbidity was consistently observed in subgroups defined by alcohol consumption and smoking status (Supplementary Tables S19-S20). However, in other subgroups, this elevated risk was only significant among males (OR = 1.21 [95% CI: 1.09–1.35]) (Supplementary Table S14), individuals aged <60 years (OR = 1.15 [95% CI: 1.05–1.27]) (Supplementary Table S15), non-rural residents (OR = 1.12 [95% CI: 1.03–1.22]) (Supplementary Table S16), those with more household members (OR = 1.11 [95% CI: 1.03–1.20]) (Supplementary Table S17), married individuals (OR = 1.15 [95% CI: 1.05–1.26]) (Supplementary Table S18), and those with higher physical activity frequency (OR = 1.13 [95% CI: 1.04–1.23]) (Supplementary Table S19).

Text S6. Additional interpretation of subgroup findings

The main findings were replicated in subgroup analyses by demographic characteristics and lifestyle behaviors, such as gender, family structure, urban–rural distribution, and lifestyle habits. Socioeconomic and healthcare gradients in cognitive-related multimorbidities were more pronounced in females than males in China. Evidence from multiple countries indicates that gender inequality plays a significant role in the higher prevalence of psychological issues among women, a trend that was more severe in China than in the US [11]. Our findings further reveal heightened risks of cognitive-related multimorbidity among Chinese women, likely due to the greater inequalities of socioeconomic status and healthcare access experienced by females in China. In addition, the inequalities of government health insurance in cognitive-related multimorbidities were more pronounced among older individuals than middle-aged individuals in China, while inequalities in other health insurance were more pronounced among middle-aged individuals. This can be attributed to the relatively high overall coverage of government health insurance, particularly among the younger population, while the elderly tend to have less access to or are less likely to purchase other health insurance. In the US, notable dose–response relationships between medical expenditure and physical–cognitive and physical–psychological–cognitive multimorbidities were observed in middle-aged individuals but not older individuals. In addition, the associations of household wealth and government health insurance with non-physical multimorbidities were more pronounced among individuals residing in rural areas compared with non-rural areas in China, whereas in the US, these associations were stronger among those living in non-rural areas than in rural areas. This result reflects significant differences in urban-rural structure between the two countries. In addition, these dose–response

relationships were more pronounced among married individuals, non-drinkers, non-smokers, and those engaging in frequent physical activity compared with other subgroups in both countries. Our study added to the evidence that socioeconomic inequalities and healthcare access in multimorbidity outcomes exist within different subgroups.

Text S7. Additional interpretation of functional findings

The associations with functional limitations further indicate that the clinical consequences of multimorbidity depend on how conditions cluster across domains. In both China and the US, cross-domain multimorbidity was more strongly associated with ADL and IADL limitations than within-domain multimorbidity, and the highest hazards were consistently observed for physical–psychological–cognitive multimorbidity[12]. This pattern is plausible because limitations in one domain may directly intensify problems in another. Physical conditions can reduce strength, mobility, and endurance; psychological symptoms may diminish motivation, social participation, and adherence to treatment; and cognitive impairment may interfere with symptom recognition, medication use, and other aspects of self-management. When these deficits occur together, their combined effect on everyday functioning is likely to be greater than that of any single domain alone. The stronger associations observed for ADL limitations than for IADL limitations should be interpreted in relation to the different clinical meanings of these outcomes. IADL limitations often appear earlier and may be shaped by environmental support, family assistance, and social context. ADL limitations usually reflect more advanced impairment in basic physical and functional capacity[13]. Therefore, complex cross-domain multimorbidity may show stronger associations with ADL limitations because it captures an accumulation of deficits severe enough to affect basic daily functioning. The generally higher hazard ratios observed in the US may likewise reflect the greater baseline complexity of multimorbidity patterns in that cohort, although differences in disease detection, reporting, and survival selection should also be considered when interpreting cross-national contrasts.

Supplementary Tables

Table S1. Variable collection

Class	Category	Variable
Basic variables	Demographic characteristics	ID
		Sex
		Age
		Areas
		HH members
		Marital status
		BMI
	Behavioral lifestyles	Drinking
		Smoking
		Activity
	Socioeconomic status	Education
		Household income
		Household wealth
	Healthcare utilization and insurance	Government health insurance
		Other health insurance
		Medical expenditure
Health variables	Physical conditions	Hypertension
		Diabetes
		Cancer
		Lung disease
		Heart problem
		Stroke
		Arthritis
		Obesity
	Psychological conditions	Feeling depressed

		Everything was an effort
		Sleep disturbances
		Lack of happiness
		Loneliness
		Inability to get going
	Cognitive conditions	Episodic memory disorder
		Working memory disorder
		Orientation disorder
Functional variables	Functional limitations	Activities of daily living (ADL) limitations
		Instrumental activities of daily living (IADL) limitations

Table S2. Imputation of missing variables based on adjacent wave

**Variable**	**CHARLS**					**HRS**				
	**Wave 1**	**Wave 2**	**Wave 3**	**Wave 4**	**Wave 5**	**Wave 1**	**Wave 2**	**Wave 3**	**Wave 4**	**Wave 5**
BMI				Wave 3	Wave 3					
Drinking										Wave 4
Smoking										Wave 4
Household wealth					Wave 4					
Other health insurance					Wave 4					
Obesity				Wave 3	Wave 3					
Episodic memory disorder									Wave 3	Wave 3
Working memory disorder									Wave 3	Wave 3
Orientation disorder									Wave 3	Wave 3
IADL limitations	Wave 2									

Table S3. Evaluation of out-of-bag error for imputation of missing values using random forest model based on non-missing values in CHARLS database

CHARLS	Wave 1		Wave 2		Wave 3		Wave 4		Wave 5		Out-of-bag error
	Non-missing values (N)	Proportion (%)	Non-missing values (N)	Proportion (%)	Non missing values (N)	Proportion (%)	Non missing values (N)	Proportion (%)	Non missing values (N)	Proportion (%)	
ID	7064	100.00	7064	100.00	7064	100.00	7064	100.00	7064	100.00	/
Sex	7064	100.00	7064	100.00	7064	100.00	7064	100.00	7064	100.00	/
Age	7064	100.00	7064	100.00	7064	100.00	7064	100.00	7064	100.00	/
Areas	7064	100.00	6561	92.88	7064	100.00	7064	100.00	7064	100.00	/
HH members	7064	100.00	6561	92.88	6591	93.30	6659	94.27	7064	100.00	0.287
Marital status	7064	100.00	6561	92.88	6591	93.30	6659	94.27	7064	100.00	0.059
BMI	5720	80.97	4954	70.13	5542	78.45	5542	78.45	5542	78.45	0.298
Drinking	6594	93.35	6507	92.11	6557	92.82	6647	94.10	7059	99.93	0.104
Smoking	6314	89.38	4910	69.51	6516	92.24	6290	89.04	7059	99.93	0.050
Activity	2858	40.46	2261	32.01	3242	45.89	6647	94.10	7059	99.93	0.233
Education	7064	100.00	7064	100.00	7064	100.00	7064	100.00	7064	100.00	/
Household income	4262	60.33	3844	54.42	2870	40.63	4805	68.02	7058	99.92	0.566
Household wealth	5084	71.97	3235	45.80	4044	57.25	5449	77.14	5449	77.14	0.256
Government health insurance	7032	99.55	6473	91.63	6575	93.08	6652	94.17	6645	94.07	0.136
Other health insurance	7032	99.55	6473	91.63	6439	91.15	6652	94.17	6652	94.17	0.045
Medical expenditure	7001	99.11	6467	91.55	6482	91.76	6541	92.60	6816	96.49	0.983
Hypertension	7021	99.39	6477	91.69	6450	91.31	6657	94.24	7064	100.00	0.049
Diabetes	6998	99.07	6442	91.19	6440	91.17	6658	94.25	7062	99.97	0.049
Cancer	7027	99.48	6486	91.82	6508	92.13	6659	94.27	7062	99.97	0.011

Lung disease	7035	99.59	6488	91.85	6514	92.21	6655	94.21	7062	99.97	0.061
Heart problem	7022	99.41	6481	91.75	6470	91.59	6655	94.21	7061	99.96	0.062
Stroke	7038	99.63	6504	92.07	6517	92.26	6658	94.25	7061	99.96	0.038
Arthritis	7045	99.73	6510	92.16	6501	92.03	6653	94.18	7061	99.96	0.071
Obesity	5729	81.10	4962	70.24	5564	78.77	5564	78.77	5564	78.77	0.013
Feeling depressed	6524	92.36	5987	84.75	6234	88.25	5978	84.63	6150	87.06	0.320
Everything was an effort	6540	92.58	5996	84.88	6240	88.34	5957	84.33	6221	88.07	0.285
Sleep disturbances	6569	92.99	6043	85.55	6309	89.31	6075	86.00	6318	89.44	0.261
Lack of happiness	6556	92.81	6024	85.28	6265	88.69	6036	85.45	6261	88.63	0.307
Loneliness	6548	92.70	6556	92.81	6276	88.84	6011	85.09	6222	88.08	0.290
Inability to get going	6529	92.43	6013	85.12	6236	88.28	5953	84.27	6197	87.73	0.259
Episodic memory disorder	5930	83.95	5769	81.67	6074	85.99	5241	74.19	6358	90.01	0.265
Working memory disorder	5876	83.18	5346	75.68	5580	78.99	4528	64.10	7064	100.00	0.168
Orientation disorder	6238	88.31	5479	77.56	5883	83.28	6114	86.55	6360	90.03	0.227
ADL limitations	6973	98.71	6503	92.06	6557	92.82	6634	93.91	7059	99.93	0.251
IADL limitations	6522	92.33	6522	92.33	6574	93.06	6645	94.07	7057	99.90	0.174

Table S4. Evaluation of out-of-bag error for imputation of missing values using random forest model based on non-missing values in HRS database

HRS	Wave 1		Wave 2		Wave 3		Wave 4		Wave 5		Out-of-bag error
	Non missing values (N)	Proportion (%)	Non missing values (N)	Proportion (%)	Non missing values (N)	Proportion (%)	Non missing values (N)	Proportion (%)	Non missing values (N)	Proportion (%)	
ID	7064	100.00	7064	100.00	7064	100.00	7064	100.00	7064	100.00	/
Sex	7064	100.00	7064	100.00	7064	100.00	7064	100.00	7064	100.00	/
Age	7064	100.00	7064	100.00	7064	100.00	7064	100.00	7064	100.00	/
Areas	7040	99.66	6971	98.68	6930	98.10	6890	97.54	6890	97.54	0.016
HH members	7064	100.00	6909	97.81	6827	96.64	6429	91.01	7064	100.00	0.170
Marital status	7063	99.99	6903	97.72	6816	96.49	6420	90.88	7048	99.77	0.043
BMI	6949	98.37	6812	96.43	6758	95.67	6356	89.98	6969	98.66	0.363
Drinking	7016	99.32	6849	96.96	6768	95.81	6386	90.40	6386	90.40	0.071
Smoking	7042	99.69	6885	97.47	6803	96.31	6400	90.60	6400	90.60	0.024
Activity	7049	99.79	6891	97.55	6806	96.35	6424	90.94	7034	99.58	0.243
Education	7064	100.00	7064	100.00	7064	100.00	7064	100.00	7064	100.00	/
Household income	7064	100.00	6909	97.81	6827	96.64	6429	91.01	7064	100.00	0.536
Household wealth	7064	100.00	6909	97.81	6827	96.64	6429	91.01	7064	100.00	0.269
Government health insurance	7023	99.42	6873	97.30	6792	96.15	6380	90.32	6975	98.74	0.118
Other health insurance	6973	98.71	6828	96.66	6725	95.20	6297	89.14	6769	95.82	0.148
Medical expenditure	7064	100.00	6909	97.81	6827	96.64	6429	91.01	7064	100.00	0.748
Hypertension	7064	100.00	6909	97.81	6827	96.64	6429	91.01	7064	100.00	0.022
Diabetes	7064	100.00	6909	97.81	6827	96.64	6429	91.01	7064	100.00	0.020
Cancer	7064	100.00	6909	97.81	6827	96.64	6429	91.01	7064	100.00	0.012

Lung disease	7064	100.00	6909	97.81	6827	96.64	6429	91.01	7064	100.00	0.008
Heart problem	7064	100.00	6909	97.81	6827	96.64	6429	91.01	7064	100.00	0.017
Stroke	7064	100.00	6909	97.81	6827	96.64	6429	91.01	7064	100.00	0.007
Arthritis	7064	100.00	6909	97.81	6827	96.64	6429	91.01	7064	100.00	0.028
Obesity	6949	98.37	6812	96.43	6758	95.67	6356	89.98	6969	98.66	0.091
Feeling depressed	6909	97.81	6775	95.91	6684	94.62	6281	88.92	6762	95.72	0.206
Everything was an effort	6903	97.72	6769	95.82	6683	94.61	6275	88.83	6756	95.64	0.197
Sleep disturbances	6904	97.73	6770	95.84	6683	94.61	6277	88.86	6756	95.64	0.239
Lack of happiness	6903	97.72	6769	95.82	6671	94.44	6261	88.63	6756	95.64	0.220
Loneliness	6908	97.79	6771	95.85	6683	94.61	6275	88.83	6764	95.75	0.205
Inability to get going	6889	97.52	6761	95.71	6668	94.39	6258	88.59	6744	95.47	0.272
Episodic memory disorder	6913	97.86	6776	95.92	6687	94.66	6687	94.66	6687	94.66	0.094
Working memory disorder	6913	97.86	6776	95.92	6687	94.66	6687	94.66	6687	94.66	0.074
Orientation disorder	1983	28.07	2257	31.95	2906	41.14	2906	41.14	2906	41.14	0.005
ADL limitations	7060	99.94	6908	97.79	6826	96.63	6424	90.94	7038	99.63	0.152
IADL limitations	7060	99.94	6908	97.79	6826	96.63	6423	90.93	7037	99.62	0.138

Table S5. Harmonized strategies for key variables included in present study

Variable	Level	CHARLS	HRS
Drinking	No	Frequency of drinking last year:<br>0. None<br>1. Less than once a month	Number of days/week that drinks =0
	Yes	2. Once a month<br>3. 2 to 3 days a month<br>4. Once a week<br>5. 2 to 3 days a week<br>6. 4 to 6 days a week<br>7. Daily<br>8. Twice a day<br>9.More than twice a day	Number of days/week that drinks >=1
Smoking	No	Does not smoke at the present time	
	Yes	Smokes at the present time	
Activity	No	0. No	4. One to three times a month.<br>5. Hardly ever or never
	Yes	The number of days of vigorous/ moderate physical activity for at least 10 minutes every week:<br>1. 1-7 days	Frequency of taking part in vigorous/moderate physical activity:<br>1. Everyday<br>2. More than once a week.<br>3. Once a week.
Education	Low	Less than upper secondary education	
	Middle	Upper secondary & vocational training	
	High	Tertiary education	
Household income	Low	The income in the first tertiles at wave level	
	Middle	The income in the second tertiles at wave level	
	High	The income in the third tertiles at wave level	
Household wealth	Low	The wealth in the first tertiles at wave level	
	Middle	The wealth in the second tertiles at wave level	
	High	The wealth in the third tertiles at wave level	

Medical expenditure	None	None medical expenditure	
	Low	Existed medical expenses and its value lower than median of the medical expenditure	
	High	Higher than median of the medical expenditure	
Hypertension, diabetes, cancer, lung disease, heart problem, stroke, or arthritis	No	None given health problem	
	Yes	Diagnosed with given health problem	
Obesity	No	BMI < 30	
	Yes	BMI ≥ 30	
Feeling depressed, everything was an effort, sleep disturbances, lack of happiness, loneliness, or inability to get going	No	Frequency of given psychology problems last week:<br>1. Rarely or none of the time < 1 day<br>2. Some or a little of the time 1-2 days	
	Yes	Frequency of given psychology problems last week:<br>3. Occasionally or a moderate amount of 3 day<br>4. Most or all of the time 5-7 days	
Episodic memory disorder	No	The score > 7	
	Yes	The score ≤ 7	
Working memory disorder	No	The score > 2	
	Yes	The score ≤ 2	
Orientation disorder	No	The score > 1	
	Yes	The score ≤ 1	
ADL limitations	No	None of ADL limitation	
	Yes	Any of following ADL limitations: bathing and showering, dressing, eating, getting in and out of bed, using the toilet, or controlling urination and defecation	Any of following ADL limitations: walking across a room, bathing, dressing, eating, getting in and out of bed, or using the toilet
IADL limitations	No	None of IADL limitation	
	Yes	Any of following IADL limitations: using the phone, managing money, taking medications, shopping for groceries, or preparing hot meals	

Table S6. Definition of eight multimorbidity categories

Multimorbidity categories	Definition	No. of physical conditions	No. of psychological conditions	No. of cognitive conditions
None multimorbidity	None condition or existence of only one condition	0	0	0
		0	0	1
		0	1	0
		1	0	0
Physical multimorbidity	The presence of at least two physical conditions without any psychological or cognitive condition	⩾2	0	0
Psychological multimorbidity	The presence of at least two psychological conditions without any physical or cognitive condition	0	⩾2	0
Cognitive multimorbidity	The presence of at least two cognitive conditions without any physical or psychological condition	0	0	⩾2
Physical–psychological multimorbidity	The presence of at least one physical and psychological condition without any cognitive condition	⩾1	⩾1	0
Physical–cognitive multimorbidity	The presence of at least one physical and cognitive condition without any psychological condition	⩾1	0	⩾1
Psychological–cognitive multimorbidity	The presence of at least one psychological and cognitive condition without any physical condition	0	⩾1	⩾1
Physical–psychological–cognitive multimorbidity	The presence of at least one physical, psychological, and cognitive condition	⩾1	⩾1	⩾1

Note: Multimorbidity was defined as the presence of at least two conditions.

Table S7. Comparison of the differences in baseline demographic characteristics and behavioral lifestyle among natural cohort, complete cohort before matching, and complete cohort after matching

	Natural cohort			Complete cohort before match			Complete cohort after match		
**Characteristic**	**China**	**The US**	***P***	**China**	**The US**	***P***	**China**	**The US**	***P***
N	17580	20554		8501	11351		7064	7064	
**Sex**			<0.001			<0.001			1.000

Male	8424	8540		3905	4446		3208	3208	
Female	9154	12014		4596	6905		3856	3856	
Unknown	2	0		0	0		0	0	
**Age (mean ± sd, years)**	58.9 ± 10.2	66.9 ± 11.6	<0.001	57.2 ± 8.8	63.6 ± 9.7	<0.001	60.1 ± 7.9	60.1 ± 7.9	1.000
**Area**			<0.001			<0.001			<0.001
Urban	7086	14779		3126	8370		2752	5307	
Rural	10494	5666		5375	2942		4312	1733	
Unknown	0	109		0	39		0	24	
**Household members (mean ± sd)**	3.6 ± 1.8	2.3 ± 1.3	<0.001	3.6 ± 1.8	2.4 ± 1.3	<0.001	3.6 ± 1.9	2.6 ± 1.4	<0.001
**Marital status**			<0.001			<0.001			<0.001
Unmarried	3470	8786		1315	4313		1227	2472	
Married	14107	11752		7186	7033		5837	4591	
Unknown	3	16		0	5		0	1	
**BMI (mean ± sd)**	23.4 ± 3.8	28.5 ± 6.2	<0.001	23.8 ± 3.8	29.0 ± 6.1	<0.001	23.6 ± 3.8	29.3 ± 6.1	<0.001
**Drinking**			<0.001			<0.001			<0.001
No	11796	17875		5694	9649		4828	5782	
Yes	4557	2540		2249	1636		1766	1234	
Unknown	1227	139		558	66		470	48	
**Smoking**			<0.001			<0.001			<0.001
No	12042	17497		5886	9789		4902	5960	
Yes	3631	2929		1805	1514		1412	1082	
Unknown	1907	128		810	48		750	22	
**Activity**			<0.001			<0.001			<0.001
No	2475	6571		1099	2750		960	1663	
Yes	4393	13901		2363	8559		1892	5371	
Unknown	10712	82		5039	42		4212	30	
**Education**			<0.001			<0.001			<0.001
Less than lower secondary	15450	3936		7466	1741		6287	1034	
Upper secondary	1774	12052		915	6666		675	4113	

Tertiary	347	4562		120	2942		102	1917	
Unknown	9	4		0	2		0	0	
**Household income**			0.994			0.996			0.994
Low	3520	6853		1599	3784		1425	2355	
Middle	3508	6850		1593	3784		1417	2354	
High	3510	6851		1595	3783		1420	2355	
Unknown	7042	0		3714	0		2802	0	
**Household wealth**			0.993			0.999			0.980
Low	4161	6862		2063	3787		1695	2365	
Middle	4162	6841		2059	3780		1696	2345	
High	4160	6851		2059	3784		1693	2354	
Unknown	5097	0		2320	0		1980	0	
**Government health insurance**			<0.001			<0.001			<0.001
No	1385	8112		565	5547		489	4398	
Yes	16070	12303		7905	5756		6543	2625	
Unknown	125	139		31	48		32	41	
**Other health insurance**			<0.001			<0.001			<0.001
No	16890	5781		8180	3006		6827	1915	
Yes	565	14369		290	8204		205	5058	
Unknown	125	404		31	141		32	91	
**Medical expenditure**			<0.001			<0.001			<0.001
None	15927	2733		7851	1347		6461	948	
Low	723	8916		284	4899		247	3058	
High	721	8905		309	5105		293	3058	
Unknown	209	0		57	0		63	0	

Table S8. Number of nodes, edges, and frequency of conditions pairs in the multimorbidity networks

Multimorbidity	China	The US

networks	Male			Female			Male			Female		
	Nodes	Edges	Frequency	Nodes	Edges	Frequency	Nodes	Edges	Frequency	Nodes	Edges	Frequency
Wave 1	16	50	10508	16	64	25395	17	61	13419	17	85	26331
Wave 2	16	53	10839	16	60	25018	16	69	15822	17	93	30318
Wave 3	16	57	13045	16	64	29324	17	63	15550	17	91	30127
Wave 4	16	62	16115	17	73	37339	17	66	18827	17	92	33701
Wave 5	16	56	17901	17	72	40317	17	68	19691	17	96	35901

Table S9. Number of nodes, edges, and frequency of conditions pairs in the psychological-cognitive multimorbidity networks

Multimorbidity networks	China						The US					
	Male			Female			Male			Female		
	Nodes	Edges	Frequency	Nodes	Edges	Frequency	Nodes	Edges	Frequency	Nodes	Edges	Frequency
Wave 1	9	31	7871	9	35	18785	9	26	5108	9	30	9319
Wave 2	9	33	8041	9	34	18833	8	21	5168	9	30	9373
Wave 3	9	32	8138	9	34	19805	9	25	4562	9	29	8587
Wave 4	9	33	10052	9	33	21896	9	24	4857	9	26	8318
Wave 5	9	30	11717	9	32	24354	9	25	4869	9	29	8804

Table S10. Number of nodes, edges, and frequency of condition pairs in the physical–cognitive multimorbidity networks

Multimorbidity networks	China						The US					
	Male			Female			Male			Female		
	Nodes	Edges	Frequency	Nodes	Edges	Frequency	Nodes	Edges	Frequency	Nodes	Edges	Frequency
Wave 1	10	11	1480	10	14	3815	11	20	5398	11	29	8469
Wave 2	10	13	1831	10	15	4558	10	20	6110	11	29	10009
Wave 3	10	13	2331	10	13	4821	11	22	7620	11	32	11878
Wave 4	10	14	3275	11	19	8717	11	21	8194	11	33	13110
Wave 5	10	15	4371	11	22	10393	11	22	9160	11	33	14332

Table S11. Number of nodes, edges, and frequency of condition pairs in the physical–psychological multimorbidity networks

Multimorbidity networks	China						The US					
	Male			Female			Male			Female		
	Nodes	Edges	Frequency	Nodes	Edges	Frequency	Nodes	Edges	Frequency	Nodes	Edges	Frequency
Wave 1	13	34	6688	13	44	14499	14	49	11670	14	62	21378
Wave 2	13	35	6604	13	41	14180	14	56	13869	14	71	21020
Wave 3	13	40	8797	13	45	18037	14	51	13874	14	68	24762
Wave 4	13	44	10614	14	54	24024	14	56	17352	14	73	29289
Wave 5	13	38	10767	14	54	25707	14	56	18010	14	74	30803

Table S12. Transition probability of multimorbidity for China participants

From	Start	Non multimorbidity condition	Physical multimorbidity	Psychological multimorbidity	Cognitive multimorbidity	Physical–psychological multimorbidity	Physical–cognitive multimorbidity	Psychological–cognitive multimorbidity	Physical–psychological–cognitive multimorbidity
Non multimorbidity condition	2011	0.544	0.011	0.052	0.035	0.087	0.055	0.155	0.062
	2013	0.546	0.028	0.031	0.028	0.084	0.103	0.109	0.071
	2015	0.467	0.036	0.036	0.032	0.109	0.099	0.127	0.094
	2018	0.461	0.024	0.035	0.050	0.101	0.092	0.144	0.093
Physical multimorbidity	2011	0.000	0.253	0.000	0.000	0.371	0.155	0.000	0.222
	2013	0.005	0.373	0.000	0.000	0.308	0.135	0.000	0.178
	2015	0.065	0.346	0.000	0.000	0.292	0.165	0.004	0.127
	2018	0.059	0.235	0.000	0.000	0.297	0.209	0.000	0.199
Psychological multimorbidit	2011	0.276	0.000	0.258	0.027	0.072	0.000	0.308	0.059
	2013	0.269	0.000	0.224	0.000	0.154	0.015	0.234	0.104

y	2015	0.308	0.000	0.183	0.008	0.158	0.017	0.183	0.142
	2018	0.279	0.000	0.183	0.019	0.115	0.038	0.250	0.115
Cognitive multimorbidity	2011	0.239	0.000	0.016	0.267	0.004	0.016	0.370	0.086
	2013	0.180	0.000	0.000	0.261	0.000	0.095	0.333	0.131
	2015	0.091	0.000	0.000	0.285	0.006	0.194	0.248	0.176
	2018	0.137	0.000	0.006	0.230	0.006	0.106	0.422	0.093
Physical–psychological multimorbidity	2011	0.074	0.071	0.001	0.000	0.430	0.098	0.000	0.325
	2013	0.087	0.086	0.002	0.000	0.390	0.094	0.002	0.339
	2015	0.063	0.088	0.014	0.000	0.422	0.079	0.014	0.319
	2018	0.061	0.051	0.014	0.001	0.422	0.078	0.016	0.357
Physical–cognitive multimorbidity	2011	0.072	0.036	0.000	0.000	0.122	0.321	0.000	0.449
	2013	0.046	0.056	0.000	0.001	0.093	0.434	0.003	0.367
	2015	0.079	0.051	0.001	0.012	0.061	0.386	0.032	0.378
	2018	0.063	0.055	0.000	0.017	0.072	0.358	0.030	0.404
Psychological–cognitive multimorbidity	2011	0.181	0.002	0.048	0.080	0.015	0.021	0.565	0.088
	2013	0.169	0.001	0.028	0.063	0.025	0.052	0.412	0.250
	2015	0.112	0.008	0.022	0.072	0.036	0.061	0.396	0.293
	2018	0.142	0.000	0.039	0.063	0.018	0.036	0.521	0.180
Physical–psychological–cognitive multimorbidity	2011	0.021	0.016	0.000	0.000	0.132	0.128	0.004	0.700
	2013	0.020	0.012	0.000	0.000	0.102	0.128	0.002	0.737
	2015	0.018	0.014	0.004	0.004	0.097	0.109	0.043	0.710
	2018	0.015	0.015	0.005	0.003	0.092	0.088	0.035	0.747

Table S13. Transition probability of multimorbidity for the US participants

From	Start	Non-multimorbidity condition	Physical multimorbidity	Psychological multimorbidity	Cognitive multimorbidity	Physical–psychological multimorbidity	Physical–cognitive multimorbidity	Psychological–cognitive multimorbidity
Non-multimorbidity condition	2012	0.709	0.066	0.025	0.003	0.104	0.051	0.021
	2014	0.667	0.088	0.022	0.010	0.101	0.065	0.020
	2016	0.737	0.088	0.010	0.000	0.139	0.008	0.012
	2018	0.754	0.087	0.018	0.000	0.125	0.006	0.009
Physical multimorbidity	2012	0.014	0.595	0.000	0.000	0.240	0.104	0.000
	2014	0.027	0.611	0.000	0.000	0.204	0.114	0.000
	2016	0.023	0.697	0.000	0.000	0.281	0.000	0.000
	2018	0.014	0.732	0.000	0.000	0.254	0.000	0.000
Psychological multimorbidity	2012	0.471	0.000	0.250	0.000	0.135	0.019	0.077
	2014	0.432	0.027	0.203	0.000	0.203	0.027	0.095
	2016	0.545	0.018	0.255	0.000	0.182	0.000	0.000
	2018	0.452	0.000	0.387	0.000	0.161	0.000	0.000
Cognitive multimorbidity	2012	0.429	0.000	0.000	0.143	0.000	0.048	0.095
	2014	0.235	0.000	0.000	0.000	0.176	0.235	0.294
	2016	0.000	0.000	0.000	0.458	0.000	0.125	0.250
	2018	0.000	0.000	0.000	0.706	0.000	0.118	0.176
Physical–psychological multimorbidity	2012	0.064	0.181	0.001	0.000	0.540	0.045	0.000
	2014	0.057	0.176	0.002	0.000	0.552	0.053	0.000
	2016	0.043	0.189	0.003	0.000	0.766	0.000	0.000
	2018	0.050	0.219	0.001	0.000	0.730	0.000	0.000
Physical–cognitive multimorbidity	2012	0.106	0.211	0.000	0.000	0.131	0.336	0.003
	2014	0.075	0.256	0.000	0.001	0.118	0.373	0.000
	2016	0.003	0.000	0.000	0.000	0.000	0.664	0.001

	2018	0.003	0.000	0.000	0.000	0.000	0.682	0.001
Psychological–cognitive multimorbidity	2012	0.301	0.000	0.048	0.048	0.038	0.027	0.376
	2014	0.276	0.009	0.052	0.069	0.112	0.034	0.328
	2016	0.130	0.000	0.000	0.065	0.000	0.076	0.522
	2018	0.208	0.000	0.000	0.056	0.000	0.083	0.444
Physical–psychological–cognitive multimorbidity	2012	0.020	0.043	0.000	0.000	0.231	0.119	0.002
	2014	0.018	0.046	0.000	0.001	0.265	0.111	0.009
	2016	0.000	0.000	0.000	0.000	0.000	0.197	0.002
	2018	0.001	0.000	0.000	0.001	0.000	0.192	0.001

Table S14. Sex-stratified analysis of socioeconomic and health factors associated with multimorbidity outcomes

**Domains**	**Risk factors**	**Sex**	**China**		**The US**	
			**OR**	***P* value**	**OR**	***P* value**
Physical multimorbidity	Education	Males	1.22 (1.00-1.48)	0.047	0.96 (0.81-1.15)	0.674
		Females	1.18 (0.91-1.54)	0.220	1.07 (0.90-1.26)	0.463
	Household income	Males	1.03 (0.92-1.16)	0.605	1.06 (0.94-1.19)	0.374
		Females	1.04 (0.90-1.20)	0.639	0.83 (0.74-0.93)	<0.001
	Household wealth	Males	0.98 (0.87-1.11)	0.771	0.84 (0.74-0.95)	0.005
		Females	1.06 (0.91-1.23)	0.452	0.76 (0.68-0.85)	<0.001
	Government health insurance	Males	1.65 (0.94-2.87)	0.078	2.41 (2.03-2.86)	<0.001
		Females	1.48 (0.85-2.59)	0.167	2.37 (2.00-2.80)	<0.001
	Other health insurance	Males	0.95 (0.59-1.51)	0.817	1.20 (0.99-1.47)	0.069
		Females	1.55 (1.00-2.41)	0.050	1.44 (1.20-1.72)	<0.001
	Medical expenditure	Males	1.21 (1.06-1.39)	0.006	1.30 (1.18-1.43)	<0.001
		Females	1.37 (1.16-1.61)	<0.001	1.13 (1.03-1.25)	0.011
Psychological multimorbidity	Education	Males	0.91 (0.70-1.19)	0.499	1.24 (0.88-1.77)	0.223
		Females	0.97 (0.71-1.33)	0.854	1.24 (0.89-1.73)	0.203

	Household income	Males	0.95 (0.81-1.10)	0.475	0.78 (0.59-1.04)	0.094
		Females	0.85 (0.73-0.99)	0.036	0.90 (0.70-1.17)	0.447
	Household wealth	Males	0.87 (0.75-1.02)	0.082	0.72 (0.54-0.95)	0.023
		Females	0.85 (0.73-0.99)	0.037	0.76 (0.59-0.98)	0.036
	Government health insurance	Males	0.87 (0.52-1.47)	0.610	0.69 (0.44-1.08)	0.101
		Females	0.98 (0.59-1.62)	0.925	0.70 (0.46-1.05)	0.083
	Other health insurance	Males	0.48 (0.22-1.03)	0.060	0.53 (0.34-0.83)	0.006
		Females	0.80 (0.44-1.44)	0.449	0.74 (0.50-1.09)	0.128
	Medical expenditure	Males	1.28 (1.07-1.54)	0.008	0.95 (0.74-1.24)	0.725
		Females	1.30 (1.07-1.58)	0.007	0.75 (0.59-0.95)	0.016
Cognitive multimorbidity	Education	Males	0.37 (0.25-0.54)	<0.001	0.23 (0.13-0.38)	<0.001
		Females	0.13 (0.07-0.26)	<0.001	0.23 (0.12-0.43)	<0.001
	Household income	Males	0.75 (0.65-0.87)	<0.001	0.70 (0.44-1.10)	0.126
		Females	0.68 (0.59-0.77)	<0.001	0.75 (0.44-1.26)	0.277
	Household wealth	Males	0.79 (0.68-0.92)	0.002	0.65 (0.42-1.00)	0.048
		Females	0.73 (0.64-0.84)	<0.001	0.63 (0.38-1.04)	0.073
	Government health insurance	Males	0.51 (0.33-0.79)	0.003	1.21 (0.67-2.18)	0.528
		Females	0.61 (0.40-0.92)	0.018	1.65 (0.82-3.35)	0.163
	Other health insurance	Males	0.25 (0.09-0.72)	0.009	0.82 (0.43-1.54)	0.534
		Females	0.59 (0.32-1.09)	0.093	0.83 (0.41-1.69)	0.606
	Medical expenditure	Males	0.80 (0.65-0.99)	0.044	0.59 (0.40-0.87)	0.008
		Females	1.02 (0.85-1.23)	0.827	0.69 (0.44-1.07)	0.097
Physical–psychological multimorbidity	Education	Males	0.83 (0.68-0.99)	0.043	0.79 (0.67-0.94)	0.009
		Females	0.96 (0.76-1.20)	0.715	0.84 (0.71-1.00)	0.049
	Household income	Males	0.99 (0.91-1.08)	0.865	0.86 (0.77-0.97)	0.012
		Females	0.83 (0.76-0.91)	<0.001	0.74 (0.66-0.82)	<0.001
	Household wealth	Males	0.85 (0.77-0.93)	<0.001	0.64 (0.56-0.72)	<0.001
		Females	0.89 (0.81-0.97)	0.012	0.59 (0.53-0.66)	<0.001

	Government health insurance	Males	1.28 (0.91-1.79)	0.163	1.87 (1.57-2.21)	<0.001
		Females	1.01 (0.74-1.39)	0.933	1.87 (1.59-2.20)	<0.001
	Other health insurance	Males	0.81 (0.56-1.17)	0.259	0.94 (0.77-1.13)	0.500
		Females	1.02 (0.73-1.43)	0.897	1.04 (0.88-1.23)	0.658
	Medical expenditure	Males	1.30 (1.18-1.44)	<0.001	1.26 (1.15-1.39)	<0.001
		Females	1.42 (1.28-1.59)	<0.001	1.12 (1.02-1.23)	0.019
Physical–cognitive multimorbidity	Education	Males	0.53 (0.43-0.64)	<0.001	0.36 (0.30-0.43)	<0.001
		Females	0.32 (0.24-0.42)	<0.001	0.35 (0.30-0.42)	<0.001
	Household income	Males	0.91 (0.83-0.99)	0.037	0.82 (0.72-0.94)	0.003
		Females	0.78 (0.71-0.85)	<0.001	0.69 (0.61-0.77)	<0.001
	Household wealth	Males	0.86 (0.79-0.95)	0.002	0.73 (0.64-0.83)	<0.001
		Females	0.90 (0.82-0.99)	0.024	0.64 (0.57-0.73)	<0.001
	Government health insurance	Males	0.99 (0.72-1.36)	0.941	2.69 (2.22-3.26)	<0.001
		Females	0.71 (0.53-0.96)	0.025	2.43 (2.03-2.92)	<0.001
	Other health insurance	Males	0.75 (0.51-1.09)	0.134	1.11 (0.90-1.37)	0.330
		Females	0.61 (0.41-0.89)	0.011	1.11 (0.92-1.35)	0.261
	Medical expenditure	Males	1.15 (1.04-1.27)	0.009	1.13 (1.02-1.26)	0.023
		Females	1.28 (1.14-1.44)	<0.001	1.09 (0.98-1.21)	0.125
Psychological–cognitive multimorbidity	Education	Males	0.40 (0.32-0.50)	<0.001	0.41 (0.31-0.54)	<0.001
		Females	0.25 (0.18-0.34)	<0.001	0.39 (0.29-0.53)	<0.001
	Household income	Males	0.79 (0.73-0.87)	<0.001	0.59 (0.47-0.75)	<0.001
		Females	0.68 (0.62-0.74)	<0.001	0.77 (0.61-0.97)	0.026
	Household wealth	Males	0.80 (0.73-0.88)	<0.001	0.49 (0.39-0.62)	<0.001
		Females	0.68 (0.62-0.75)	<0.001	0.62 (0.49-0.78)	<0.001
	Government health insurance	Males	0.85 (0.63-1.15)	0.301	0.62 (0.44-0.88)	0.008
		Females	0.67 (0.51-0.89)	0.006	0.81 (0.57-1.15)	0.238
	Other health insurance	Males	0.55 (0.35-0.84)	0.006	0.68 (0.48-0.96)	0.026
		Females	0.59 (0.40-0.87)	0.008	0.87 (0.62-1.22)	0.403

Physical–psychological–cognitive multimorbidity	Medical expenditure	Males	1.17 (1.04-1.30)	0.007	0.69 (0.57-0.84)	<0.001
		Females	1.26 (1.12-1.42)	<0.001	0.66 (0.54-0.81)	<0.001
	Education	Males	0.32 (0.26-0.40)	<0.001	0.27 (0.23-0.33)	<0.001
		Females	0.19 (0.14-0.25)	<0.001	0.25 (0.21-0.30)	<0.001
	Household income	Males	0.80 (0.74-0.87)	<0.001	0.61 (0.54-0.69)	<0.001
		Females	0.70 (0.65-0.76)	<0.001	0.57 (0.51-0.64)	<0.001
	Household wealth	Males	0.72 (0.66-0.78)	<0.001	0.51 (0.45-0.59)	<0.001
		Females	0.68 (0.62-0.74)	<0.001	0.42 (0.37-0.47)	<0.001
	Government health insurance	Males	0.88 (0.66-1.17)	0.379	2.57 (2.13-3.11)	<0.001
		Females	0.68 (0.52-0.88)	0.003	2.65 (2.22-3.15)	<0.001
	Other health insurance	Males	0.71 (0.50-1.02)	0.061	0.93 (0.76-1.14)	0.469
		Females	0.55 (0.39-0.76)	<0.001	0.89 (0.75-1.07)	0.206
	Medical expenditure	Males	1.37 (1.25-1.51)	<0.001	1.21 (1.09-1.35)	<0.001
		Females	1.50 (1.36-1.66)	<0.001	1.02 (0.92-1.12)	0.733

Table S15. Age-stratified analysis of socioeconomic and health factors associated with multimorbidity outcomes

Domains	Risk factors	Age	China		The US	
			OR	*P* value	OR	*P* value
Physical multimorbidity	Education	< 60	1.12 (0.91-1.38)	0.291	0.78 (0.66-0.93)	0.005
		≥ 60	1.35 (1.13-1.62)	0.001	1.21 (1.01-1.44)	0.039
	Household income	< 60	1.06 (0.94-1.20)	0.352	0.98 (0.87-1.10)	0.743
		≥ 60	1.08 (0.95-1.23)	0.220	0.92 (0.82-1.04)	0.171
	Household wealth	< 60	0.95 (0.84-1.09)	0.485	0.71 (0.63-0.80)	<0.001
		≥ 60	1.11 (0.97-1.26)	0.123	0.74 (0.65-0.84)	<0.001
	Government health insurance	< 60	1.29 (0.78-2.12)	0.325	1.77 (1.45-2.17)	<0.001
		≥ 60	2.14 (1.14-4.00)	0.018	1.89 (1.55-2.29)	<0.001
	Other health insurance	< 60	1.36 (0.93-1.99)	0.116	1.49 (1.22-1.80)	<0.001
		≥ 60	1.21 (0.70-2.09)	0.494	1.17 (0.97-1.42)	0.097

	Medical expenditure	< 60	1.22 (1.06-1.41)	0.005	1.22 (1.11-1.34)	<0.001
		≥ 60	1.37 (1.18-1.59)	<0.001	1.08 (0.98-1.20)	0.129
Psychological multimorbidity	Education	< 60	0.91 (0.72-1.15)	0.426	1.04 (0.81-1.34)	0.744
		≥ 60	0.76 (0.51-1.11)	0.157	1.95 (1.23-3.10)	0.005
	Household income	< 60	0.91 (0.80-1.03)	0.141	0.85 (0.70-1.04)	0.110
		≥ 60	0.92 (0.76-1.12)	0.421	0.83 (0.58-1.17)	0.277
	Household wealth	< 60	0.83 (0.73-0.95)	0.006	0.82 (0.68-1.00)	0.047
		≥ 60	0.88 (0.73-1.07)	0.203	0.59 (0.42-0.84)	0.004
	Government health insurance	< 60	0.91 (0.60-1.38)	0.657	1.18 (0.81-1.71)	0.387
		≥ 60	1.09 (0.51-2.32)	0.830	0.97 (0.57-1.66)	0.925
	Other health insurance	< 60	0.64 (0.38-1.06)	0.082	0.64 (0.47-0.88)	0.005
		≥ 60	0.70 (0.24-2.00)	0.502	0.74 (0.44-1.27)	0.279
	Medical expenditure	< 60	1.23 (1.06-1.44)	0.008	0.98 (0.82-1.16)	0.776
		≥ 60	1.39 (1.10-1.76)	0.007	0.75 (0.54-1.05)	0.096
Cognitive multimorbidity	Education	< 60	0.28 (0.19-0.40)	<0.001	0.24 (0.14-0.39)	<0.001
		≥ 60	0.16 (0.08-0.34)	<0.001	0.21 (0.11-0.39)	<0.001
	Household income	< 60	0.77 (0.68-0.88)	<0.001	0.64 (0.41-0.98)	0.039
		≥ 60	0.71 (0.62-0.82)	<0.001	0.75 (0.44-1.26)	0.272
	Household wealth	< 60	0.77 (0.67-0.88)	<0.001	0.61 (0.40-0.94)	0.025
		≥ 60	0.76 (0.67-0.88)	<0.001	0.80 (0.49-1.32)	0.388
	Government health insurance	< 60	0.68 (0.45-1.03)	0.068	1.26 (0.65-2.43)	0.492
		≥ 60	0.43 (0.29-0.65)	<0.001	1.12 (0.47-2.67)	0.804
	Other health insurance	< 60	0.45 (0.24-0.85)	0.014	0.87 (0.48-1.59)	0.660
		≥ 60	0.44 (0.18-1.04)	0.062	0.77 (0.38-1.58)	0.473
	Medical expenditure	< 60	0.95 (0.80-1.14)	0.602	0.68 (0.47-0.97)	0.034
		≥ 60	0.83 (0.67-1.02)	0.075	0.54 (0.35-0.84)	0.007
Physical–psychological multimorbidity	Education	< 60	0.75 (0.62-0.90)	0.003	0.71 (0.60-0.84)	<0.001
		≥ 60	0.95 (0.80-1.12)	0.534	0.88 (0.73-1.05)	0.147

	Household income	< 60	0.91 (0.83-0.99)	0.021	0.80 (0.72-0.89)	<0.001
		≥ 60	0.97 (0.89-1.06)	0.568	0.78 (0.69-0.87)	<0.001
	Household wealth	< 60	0.85 (0.78-0.93)	<0.001	0.59 (0.53-0.66)	<0.001
		≥ 60	0.92 (0.84-1.00)	0.052	0.63 (0.55-0.72)	<0.001
	Government health insurance	< 60	1.11 (0.83-1.50)	0.476	2.13 (1.77-2.57)	<0.001
		≥ 60	1.18 (0.84-1.66)	0.343	1.38 (1.13-1.67)	0.001
	Other health insurance	< 60	0.99 (0.74-1.34)	0.962	1.07 (0.90-1.27)	0.475
		≥ 60	0.90 (0.60-1.37)	0.626	0.98 (0.82-1.18)	0.863
	Medical expenditure	< 60	1.29 (1.17-1.42)	<0.001	1.23 (1.13-1.34)	<0.001
		≥ 60	1.46 (1.32-1.63)	<0.001	1.09 (0.98-1.21)	0.099
Physical–cognitive multimorbidity	Education	< 60	0.41 (0.33-0.50)	<0.001	0.35 (0.29-0.42)	<0.001
		≥ 60	0.54 (0.44-0.66)	<0.001	0.36 (0.30-0.43)	<0.001
	Household income	< 60	0.93 (0.85-1.01)	0.098	0.78 (0.69-0.89)	<0.001
		≥ 60	0.84 (0.77-0.91)	<0.001	0.74 (0.65-0.84)	<0.001
	Household wealth	< 60	0.88 (0.80-0.97)	0.007	0.63 (0.55-0.71)	<0.001
		≥ 60	0.94 (0.86-1.02)	0.130	0.66 (0.57-0.75)	<0.001
	Government health insurance	< 60	0.92 (0.68-1.24)	0.580	2.02 (1.63-2.50)	<0.001
		≥ 60	0.78 (0.57-1.06)	0.108	1.98 (1.58-2.48)	<0.001
	Other health insurance	< 60	0.75 (0.53-1.06)	0.100	1.16 (0.95-1.42)	0.143
		≥ 60	0.70 (0.46-1.07)	0.100	1.03 (0.84-1.25)	0.782
	Medical expenditure	< 60	1.18 (1.06-1.31)	0.002	1.14 (1.03-1.27)	0.012
		≥ 60	1.23 (1.11-1.37)	<0.001	0.96 (0.86-1.08)	0.524
Psychological–cognitive multimorbidity	Education	< 60	0.33 (0.26-0.41)	<0.001	0.37 (0.29-0.47)	<0.001
		≥ 60	0.29 (0.22-0.39)	<0.001	0.52 (0.35-0.75)	<0.001
	Household income	< 60	0.80 (0.73-0.87)	<0.001	0.66 (0.55-0.80)	<0.001
		≥ 60	0.70 (0.64-0.77)	<0.001	0.72 (0.53-0.98)	0.036
	Household wealth	< 60	0.75 (0.68-0.81)	<0.001	0.63 (0.52-0.77)	<0.001
		≥ 60	0.76 (0.70-0.84)	<0.001	0.48 (0.36-0.65)	<0.001

Physical–psychological–cognitive multimorbidity	Government health insurance	< 60	0.71 (0.55-0.93)	0.013	0.92 (0.66-1.29)	0.644
		≥ 60	0.68 (0.50-0.92)	0.012	0.79 (0.50-1.24)	0.305
	Other health insurance	< 60	0.54 (0.38-0.78)	<0.001	0.74 (0.56-0.97)	0.029
		≥ 60	0.67 (0.42-1.06)	0.089	0.62 (0.40-0.96)	0.031
	Medical expenditure	< 60	1.18 (1.06-1.31)	0.002	0.75 (0.65-0.88)	<0.001
		≥ 60	1.20 (1.07-1.35)	0.002	0.60 (0.46-0.79)	<0.001
	Education	< 60	0.21 (0.17-0.26)	<0.001	0.27 (0.23-0.33)	<0.001
		≥ 60	0.29 (0.23-0.36)	<0.001	0.23 (0.19-0.27)	<0.001
	Household income	< 60	0.79 (0.74-0.86)	<0.001	0.59 (0.53-0.67)	<0.001
		≥ 60	0.77 (0.71-0.83)	<0.001	0.59 (0.52-0.68)	<0.001
	Household wealth	< 60	0.68 (0.62-0.73)	<0.001	0.45 (0.40-0.51)	<0.001
		≥ 60	0.75 (0.69-0.81)	<0.001	0.45 (0.40-0.52)	<0.001
	Government health insurance	< 60	0.91 (0.70-1.17)	0.452	2.98 (2.44-3.63)	<0.001
		≥ 60	0.66 (0.50-0.86)	0.002	1.67 (1.34-2.09)	<0.001
	Other health insurance	< 60	0.62 (0.45-0.84)	0.002	0.95 (0.79-1.14)	0.557
		≥ 60	0.73 (0.50-1.07)	0.111	0.92 (0.76-1.12)	0.412
	Medical expenditure	< 60	1.38 (1.26-1.51)	<0.001	1.15 (1.05-1.27)	0.003
		≥ 60	1.45 (1.32-1.60)	<0.001	1.00 (0.89-1.11)	0.946

Table S16. Whether lived in rural—stratified analysis of socioeconomic and health factors associated with multimorbidity outcomes

**Domains**	**Risk factors**	**Whether lived in rural**	**China**		**The US**	
			**OR**	***P* value**	**OR**	***P* value**
Physical multimorbidity	Education	No	1.06 (0.88-1.29)	0.529	1.02 (0.89-1.17)	0.808
		Yes	1.07 (0.77-1.49)	0.690	0.99 (0.76-1.28)	0.926
	Household income	No	1.09 (0.95-1.24)	0.215	0.94 (0.85-1.03)	0.171

		Yes	0.92 (0.80-1.05)	0.231	0.94 (0.80-1.11)	0.454
	Household wealth	No	0.98 (0.85-1.13)	0.794	0.80 (0.73-0.88)	<0.001
		Yes	0.92 (0.80-1.06)	0.257	0.74 (0.62-0.89)	0.001
	Government health insurance	No	1.53 (0.93-2.52)	0.097	2.35 (2.04-2.69)	<0.001
		Yes	1.52 (0.79-2.89)	0.207	2.56 (2.00-3.29)	<0.001
	Other health insurance	No	1.20 (0.81-1.77)	0.372	1.34 (1.15-1.56)	<0.001
		Yes	1.13 (0.63-2.04)	0.678	1.27 (0.96-1.68)	0.092
	Medical expenditure	No	1.23 (1.08-1.41)	0.002	1.22 (1.12-1.31)	<0.001
		Yes	1.31 (1.11-1.56)	0.002	1.21 (1.05-1.40)	0.010
Psychological multimorbidity	Education	No	0.84 (0.65-1.09)	0.196	1.26 (0.97-1.64)	0.077
		Yes	1.06 (0.76-1.49)	0.722	1.14 (0.60-2.15)	0.693
	Household income	No	0.89 (0.75-1.07)	0.211	0.80 (0.65-0.99)	0.036
		Yes	0.97 (0.84-1.11)	0.627	1.08 (0.65-1.80)	0.754
	Household wealth	No	0.99 (0.83-1.20)	0.957	0.76 (0.62-0.93)	0.009
		Yes	0.84 (0.73-0.97)	0.021	0.64 (0.39-1.06)	0.084
	Government health insurance	No	0.90 (0.54-1.51)	0.697	0.71 (0.51-0.99)	0.045
		Yes	0.90 (0.54-1.50)	0.693	0.58 (0.27-1.27)	0.175
	Other health insurance	No	0.75 (0.42-1.35)	0.345	0.64 (0.47-0.89)	0.007
		Yes	0.58 (0.27-1.25)	0.162	0.64 (0.30-1.37)	0.248
	Medical expenditure	No	1.19 (0.97-1.44)	0.088	0.84 (0.70-1.02)	0.077
		Yes	1.36 (1.13-1.63)	<0.001	0.89 (0.56-1.41)	0.607
Cognitive multimorbidity	Education	No	0.27 (0.17-0.42)	<0.001	0.24 (0.16-0.37)	<0.001
		Yes	0.23 (0.14-0.38)	<0.001	0.15 (0.05-0.49)	0.002
	Household income	No	0.83 (0.69-0.99)	0.038	0.77 (0.54-1.11)	0.165
		Yes	0.76 (0.67-0.86)	<0.001	0.36 (0.11-1.13)	0.080
	Household wealth	No	0.86 (0.72-1.04)	0.119	0.65 (0.46-0.92)	0.015
		Yes	0.79 (0.70-0.90)	<0.001	0.53 (0.19-1.51)	0.236
	Government health	No	0.57 (0.34-0.94)	0.028	1.49 (0.92-2.42)	0.104

	insurance	Yes	0.52 (0.36-0.76)	<0.001	0.81 (0.22-2.93)	0.744
	Other health insurance	No	0.50 (0.24-1.05)	0.067	0.73 (0.44-1.22)	0.229
		Yes	0.47 (0.23-0.98)	0.043	2.06 (0.50-8.47)	0.315
	Medical expenditure	No	0.90 (0.72-1.13)	0.361	0.64 (0.47-0.87)	0.004
		Yes	0.92 (0.76-1.10)	0.341	0.56 (0.25-1.26)	0.157
Physical–psychological multimorbidity	Education	No	0.78 (0.65-0.94)	0.008	0.86 (0.75-0.98)	0.024
		Yes	0.79 (0.61-1.03)	0.081	0.71 (0.55-0.93)	0.011
	Household income	No	0.94 (0.85-1.04)	0.243	0.77 (0.70-0.84)	<0.001
		Yes	0.90 (0.83-0.98)	0.015	0.84 (0.71-0.99)	0.036
	Household wealth	No	0.91 (0.82-1.02)	0.097	0.62 (0.57-0.68)	<0.001
		Yes	0.84 (0.77-0.92)	<0.001	0.57 (0.47-0.68)	<0.001
	Government health insurance	No	1.22 (0.88-1.70)	0.236	1.85 (1.62-2.12)	<0.001
		Yes	1.00 (0.72-1.39)	0.989	1.88 (1.48-2.41)	<0.001
	Other health insurance	No	1.01 (0.73-1.39)	0.970	0.99 (0.85-1.14)	0.855
		Yes	0.86 (0.58-1.27)	0.443	0.99 (0.75-1.29)	0.921
	Medical expenditure	No	1.35 (1.21-1.49)	<0.001	1.19 (1.10-1.28)	<0.001
		Yes	1.34 (1.20-1.49)	<0.001	1.20 (1.05-1.39)	0.010
Physical–cognitive multimorbidity	Education	No	0.50 (0.41-0.60)	<0.001	0.36 (0.31-0.41)	<0.001
		Yes	0.26 (0.19-0.35)	<0.001	0.36 (0.28-0.48)	<0.001
	Household income	No	0.84 (0.76-0.93)	<0.001	0.75 (0.68-0.83)	<0.001
		Yes	0.90 (0.83-0.98)	0.011	0.74 (0.62-0.89)	0.001
	Household wealth	No	0.96 (0.86-1.07)	0.498	0.66 (0.59-0.73)	<0.001
		Yes	0.85 (0.78-0.93)	<0.001	0.78 (0.64-0.95)	0.012
	Government health insurance	No	0.73 (0.53-0.99)	0.045	2.50 (2.15-2.90)	<0.001
		Yes	0.88 (0.65-1.20)	0.425	2.76 (2.10-3.64)	<0.001
	Other health insurance	No	0.71 (0.50-1.01)	0.057	1.04 (0.88-1.22)	0.661
		Yes	0.62 (0.40-0.96)	0.031	1.44 (1.06-1.95)	0.019
	Medical expenditure	No	1.22 (1.10-1.37)	<0.001	1.13 (1.04-1.23)	0.004

		Yes	1.17 (1.05-1.30)	0.004	1.05 (0.89-1.23)	0.548
Psychological–cognitive multimorbidity	Education	No	0.34 (0.27-0.43)	<0.001	0.44 (0.35-0.55)	<0.001
		Yes	0.34 (0.25-0.45)	<0.001	0.21 (0.12-0.36)	<0.001
	Household income	No	0.73 (0.65-0.81)	<0.001	0.62 (0.52-0.74)	<0.001
		Yes	0.82 (0.75-0.88)	<0.001	1.11 (0.74-1.68)	0.609
	Household wealth	No	0.79 (0.71-0.89)	<0.001	0.57 (0.48-0.68)	<0.001
		Yes	0.79 (0.72-0.85)	<0.001	0.46 (0.30-0.71)	<0.001
	Government health insurance	No	0.76 (0.55-1.05)	0.092	0.73 (0.55-0.95)	0.020
		Yes	0.68 (0.52-0.90)	0.006	0.66 (0.36-1.20)	0.176
	Other health insurance	No	0.67 (0.44-1.00)	0.052	0.78 (0.60-1.01)	0.059
		Yes	0.57 (0.38-0.85)	0.007	0.72 (0.40-1.31)	0.289
	Medical expenditure	No	1.24 (1.09-1.40)	<0.001	0.69 (0.59-0.80)	<0.001
		Yes	1.18 (1.07-1.32)	0.002	0.59 (0.42-0.85)	0.004
Physical–psychological–cognitive multimorbidity	Education	No	0.29 (0.23-0.36)	<0.001	0.28 (0.24-0.32)	<0.001
		Yes	0.15 (0.11-0.20)	<0.001	0.21 (0.16-0.27)	<0.001
	Household income	No	0.76 (0.70-0.84)	<0.001	0.58 (0.53-0.64)	<0.001
		Yes	0.81 (0.75-0.87)	<0.001	0.59 (0.49-0.71)	<0.001
	Household wealth	No	0.76 (0.69-0.84)	<0.001	0.44 (0.40-0.49)	<0.001
		Yes	0.71 (0.66-0.76)	<0.001	0.52 (0.43-0.63)	<0.001
	Government health insurance	No	0.77 (0.58-1.02)	0.072	2.68 (2.32-3.11)	<0.001
		Yes	0.71 (0.55-0.91)	0.008	2.38 (1.82-3.11)	<0.001
	Other health insurance	No	0.65 (0.46-0.91)	0.013	0.91 (0.78-1.05)	0.203
		Yes	0.63 (0.44-0.89)	0.010	0.89 (0.67-1.18)	0.422
	Medical expenditure	No	1.39 (1.26-1.53)	<0.001	1.12 (1.03-1.22)	0.006
		Yes	1.44 (1.32-1.58)	<0.001	1.09 (0.93-1.27)	0.284

Table S17. Household members level-stratified analysis of socioeconomic and health factors associated with multimorbidity outcomes

Domains	Risk factors	HH members level	China		The US	
			**OR**	***P* value**	**OR**	***P* value**
Physical multimorbidity	Education	Low	1.06 (0.82-1.36)	0.670	1.18 (0.85-1.65)	0.329
		High	1.27 (1.04-1.55)	0.020	0.92 (0.81-1.05)	0.235
	Household income	Low	1.03 (0.89-1.18)	0.715	0.95 (0.77-1.18)	0.653
		High	1.13 (1.00-1.28)	0.055	0.94 (0.86-1.03)	0.170
	Household wealth	Low	1.11 (0.96-1.29)	0.161	0.67 (0.54-0.83)	<0.001
		High	0.96 (0.84-1.08)	0.484	0.81 (0.73-0.88)	<0.001
	Government health insurance	Low	1.44 (0.81-2.56)	0.210	3.12 (2.24-4.33)	<0.001
		High	1.75 (1.00-3.06)	0.050	2.41 (2.11-2.74)	<0.001
	Other health insurance	Low	1.32 (0.80-2.20)	0.280	1.39 (0.97-1.99)	0.071
		High	1.20 (0.79-1.81)	0.387	1.36 (1.18-1.57)	<0.001
	Medical expenditure	Low	1.27 (1.08-1.49)	0.004	1.08 (0.90-1.31)	0.394
		High	1.24 (1.08-1.42)	0.002	1.21 (1.12-1.30)	<0.001
Psychological multimorbidity	Education	Low	0.80 (0.57-1.14)	0.219	1.05 (0.60-1.86)	0.859
		High	0.99 (0.78-1.26)	0.920	1.23 (0.98-1.56)	0.077
	Household income	Low	1.00 (0.84-1.20)	0.999	1.09 (0.71-1.66)	0.689
		High	0.90 (0.78-1.02)	0.109	0.87 (0.73-1.05)	0.161
	Household wealth	Low	0.80 (0.67-0.96)	0.017	0.74 (0.50-1.10)	0.140
		High	0.90 (0.78-1.03)	0.114	0.72 (0.60-0.86)	<0.001
	Government health insurance	Low	1.13 (0.60-2.13)	0.714	0.98 (0.53-1.82)	0.957
		High	0.86 (0.55-1.34)	0.510	0.67 (0.50-0.89)	0.006
	Other health insurance	Low	0.60 (0.27-1.36)	0.224	0.64 (0.33-1.24)	0.185
		High	0.72 (0.41-1.25)	0.242	0.62 (0.47-0.82)	<0.001
	Medical expenditure	Low	1.27 (1.02-1.58)	0.036	0.94 (0.64-1.36)	0.734
		High	1.27 (1.07-1.49)	0.005	0.86 (0.73-1.02)	0.085
Cognitive	Education	Low	0.21 (0.12-0.38)	<0.001	0.29 (0.09-0.92)	0.035

multimorbidity		High	0.27 (0.18-0.40)	<0.001	0.21 (0.14-0.33)	<0.001
	Household income	Low	0.75 (0.64-0.88)	<0.001	0.83 (0.32-2.17)	0.706
		High	0.75 (0.66-0.85)	<0.001	0.66 (0.46-0.95)	0.023
	Household wealth	Low	0.76 (0.65-0.89)	<0.001	0.63 (0.26-1.58)	0.327
		High	0.78 (0.68-0.88)	<0.001	0.72 (0.51-1.01)	0.056
	Government health insurance	Low	0.46 (0.30-0.71)	<0.001	1.50 (0.42-5.34)	0.528
		High	0.62 (0.41-0.92)	0.018	1.31 (0.82-2.09)	0.264
	Other health insurance	Low	0.56 (0.27-1.16)	0.118	1.80 (0.49-6.53)	0.374
		High	0.37 (0.17-0.77)	0.008	0.71 (0.44-1.16)	0.177
	Medical expenditure	Low	0.94 (0.75-1.17)	0.574	0.31 (0.13-0.74)	0.008
		High	0.89 (0.75-1.06)	0.193	0.68 (0.51-0.92)	0.011
Physical–psychological multimorbidity	Education	Low	0.76 (0.60-0.96)	0.024	0.90 (0.65-1.24)	0.504
		High	0.84 (0.70-1.00)	0.056	0.75 (0.66-0.86)	<0.001
	Household income	Low	1.03 (0.93-1.14)	0.600	0.81 (0.66-1.00)	0.055
		High	0.89 (0.82-0.96)	0.004	0.81 (0.74-0.88)	<0.001
	Household wealth	Low	0.86 (0.78-0.96)	0.007	0.54 (0.43-0.66)	<0.001
		High	0.90 (0.82-0.97)	0.010	0.63 (0.58-0.69)	<0.001
	Government health insurance	Low	0.97 (0.68-1.38)	0.854	2.64 (1.93-3.61)	<0.001
		High	1.25 (0.92-1.68)	0.151	1.78 (1.57-2.02)	<0.001
	Other health insurance	Low	0.94 (0.62-1.43)	0.789	1.09 (0.78-1.52)	0.612
		High	0.94 (0.69-1.27)	0.678	1.01 (0.88-1.15)	0.935
	Medical expenditure	Low	1.30 (1.15-1.47)	<0.001	1.04 (0.87-1.24)	0.685
		High	1.35 (1.23-1.49)	<0.001	1.20 (1.12-1.29)	<0.001
Physical–cognitive multimorbidity	Education	Low	0.44 (0.34-0.57)	<0.001	0.43 (0.30-0.61)	<0.001
		High	0.41 (0.33-0.50)	<0.001	0.33 (0.29-0.38)	<0.001
	Household income	Low	0.83 (0.74-0.92)	<0.001	0.86 (0.68-1.09)	0.211
		High	0.90 (0.83-0.97)	0.009	0.76 (0.69-0.83)	<0.001
	Household wealth	Low	0.92 (0.82-1.02)	0.114	0.58 (0.46-0.73)	<0.001

		High	0.88 (0.81-0.96)	0.004	0.71 (0.64-0.78)	<0.001
	Government health insurance	Low	0.80 (0.57-1.13)	0.212	3.24 (2.27-4.62)	<0.001
		High	0.87 (0.66-1.16)	0.346	2.51 (2.18-2.89)	<0.001
	Other health insurance	Low	0.75 (0.48-1.17)	0.210	1.29 (0.90-1.85)	0.171
		High	0.67 (0.48-0.95)	0.024	1.11 (0.95-1.29)	0.182
	Medical expenditure	Low	1.20 (1.06-1.36)	0.004	0.89 (0.73-1.08)	0.231
		High	1.18 (1.07-1.30)	0.001	1.13 (1.04-1.22)	0.004
Psychological–cognitive multimorbidity	Education	Low	0.22 (0.15-0.31)	<0.001	0.58 (0.32-1.06)	0.075
		High	0.36 (0.29-0.45)	<0.001	0.40 (0.32-0.49)	<0.001
	Household income	Low	0.71 (0.63-0.79)	<0.001	0.85 (0.52-1.38)	0.505
		High	0.76 (0.71-0.83)	<0.001	0.67 (0.56-0.79)	<0.001
	Household wealth	Low	0.69 (0.62-0.77)	<0.001	0.41 (0.26-0.65)	<0.001
		High	0.77 (0.71-0.84)	<0.001	0.59 (0.50-0.70)	<0.001
	Government health insurance	Low	0.63 (0.46-0.87)	0.005	0.75 (0.40-1.40)	0.364
		High	0.80 (0.62-1.04)	0.091	0.75 (0.58-0.96)	0.023
	Other health insurance	Low	0.70 (0.44-1.10)	0.121	0.74 (0.39-1.41)	0.362
		High	0.51 (0.36-0.75)	<0.001	0.67 (0.53-0.86)	0.002
	Medical expenditure	Low	1.20 (1.05-1.37)	0.006	0.67 (0.47-0.97)	0.034
		High	1.19 (1.08-1.32)	<0.001	0.69 (0.60-0.79)	<0.001
Physical–psychological–cognitive multimorbidity	Education	Low	0.22 (0.16-0.29)	<0.001	0.27 (0.19-0.38)	<0.001
		High	0.22 (0.18-0.27)	<0.001	0.25 (0.22-0.28)	<0.001
	Household income	Low	0.76 (0.69-0.83)	<0.001	0.72 (0.56-0.91)	0.007
		High	0.79 (0.73-0.85)	<0.001	0.58 (0.53-0.64)	<0.001
	Household wealth	Low	0.72 (0.65-0.79)	<0.001	0.38 (0.30-0.48)	<0.001
		High	0.69 (0.64-0.75)	<0.001	0.47 (0.43-0.52)	<0.001
	Government health insurance	Low	0.67 (0.50-0.90)	0.008	3.36 (2.39-4.74)	<0.001
		High	0.82 (0.65-1.05)	0.113	2.54 (2.21-2.91)	<0.001
	Other health insurance	Low	0.68 (0.46-1.01)	0.059	1.07 (0.76-1.52)	0.694

		High	0.61 (0.45-0.82)	0.001	0.91 (0.79-1.05)	0.189
	Medical expenditure	Low	1.40 (1.25-1.56)	<0.001	0.97 (0.81-1.17)	0.751
		High	1.41 (1.29-1.53)	<0.001	1.11 (1.03-1.20)	0.007

Table S18. Marital status—stratified analysis of socioeconomic and health factors associated with multimorbidity outcomes

**Domains**	**Risk factors**	**Marital status**	**China**		**The US**	
			**OR**	***P* value**	**OR**	***P* value**
Physical multimorbidity	Education	Other	0.79 (0.44-1.43)	0.431	1.12 (0.90-1.39)	0.297
		Married	1.20 (1.02-1.41)	0.028	0.97 (0.83-1.13)	0.677
	Household income	Other	1.16 (0.89-1.53)	0.274	0.94 (0.81-1.09)	0.415
		Married	1.03 (0.93-1.14)	0.557	0.94 (0.85-1.03)	0.189
	Household wealth	Other	1.25 (0.96-1.64)	0.103	0.72 (0.62-0.83)	<0.001
		Married	0.99 (0.90-1.10)	0.910	0.83 (0.75-0.93)	<0.001
	Government health insurance	Other	2.67 (0.79-9.01)	0.114	2.74 (2.21-3.40)	<0.001
		Married	1.43 (0.94-2.16)	0.092	2.28 (1.98-2.64)	<0.001
	Other health insurance	Other	2.09 (0.97-4.47)	0.059	1.70 (1.35-2.15)	<0.001
		Married	1.14 (0.81-1.62)	0.448	1.19 (1.01-1.40)	0.037
	Medical expenditure	Other	0.92 (0.65-1.31)	0.653	1.25 (1.10-1.41)	<0.001
		Married	1.30 (1.16-1.45)	<0.001	1.20 (1.10-1.30)	<0.001
Psychological multimorbidity	Education	Other	0.86 (0.46-1.63)	0.645	0.98 (0.64-1.48)	0.906
		Married	0.91 (0.73-1.12)	0.363	1.39 (1.04-1.88)	0.028
	Household income	Other	1.05 (0.78-1.40)	0.743	1.07 (0.77-1.49)	0.686
		Married	0.91 (0.81-1.02)	0.106	0.79 (0.62-1.01)	0.060
	Household wealth	Other	0.97 (0.73-1.28)	0.807	0.78 (0.57-1.08)	0.133
		Married	0.85 (0.76-0.96)	0.008	0.76 (0.60-0.96)	0.021
	Government health insurance	Other	1.14 (0.45-2.86)	0.784	0.90 (0.55-1.47)	0.678
		Married	0.87 (0.59-1.29)	0.487	0.57 (0.38-0.84)	0.005

	Other health insurance	Other	1.51 (0.63-3.65)	0.356	0.59 (0.36-0.96)	0.033
		Married	0.56 (0.33-0.95)	0.033	0.66 (0.45-0.96)	0.030
	Medical expenditure	Other	1.08 (0.73-1.61)	0.692	0.90 (0.67-1.20)	0.455
		Married	1.31 (1.13-1.50)	<0.001	0.81 (0.65-1.01)	0.058
Cognitive multimorbidity	Education	Other	0.21 (0.08-0.52)	<0.001	0.28 (0.15-0.54)	<0.001
		Married	0.25 (0.17-0.36)	<0.001	0.19 (0.11-0.33)	<0.001
	Household income	Other	0.83 (0.67-1.02)	0.076	0.59 (0.31-1.12)	0.106
		Married	0.72 (0.64-0.80)	<0.001	0.83 (0.55-1.28)	0.404
	Household wealth	Other	0.78 (0.63-0.95)	0.016	0.74 (0.43-1.29)	0.294
		Married	0.77 (0.69-0.87)	<0.001	0.62 (0.41-0.93)	0.020
	Government health insurance	Other	0.62 (0.33-1.16)	0.134	2.06 (0.98-4.30)	0.056
		Married	0.55 (0.39-0.77)	<0.001	1.09 (0.61-1.95)	0.772
	Other health insurance	Other	0.09 (0.01-0.66)	0.018	1.22 (0.58-2.54)	0.604
		Married	0.56 (0.33-0.96)	0.035	0.67 (0.36-1.22)	0.191
	Medical expenditure	Other	0.92 (0.69-1.25)	0.607	0.58 (0.36-0.94)	0.025
		Married	0.89 (0.76-1.04)	0.149	0.62 (0.43-0.90)	0.011
Physical–psychological multimorbidity	Education	Other	1.06 (0.73-1.54)	0.776	0.94 (0.76-1.16)	0.588
		Married	0.80 (0.69-0.93)	0.004	0.75 (0.65-0.87)	<0.001
	Household income	Other	0.84 (0.72-0.98)	0.029	0.77 (0.67-0.89)	<0.001
		Married	0.94 (0.88-1.01)	0.101	0.84 (0.76-0.93)	<0.001
	Household wealth	Other	0.88 (0.76-1.03)	0.102	0.59 (0.51-0.68)	<0.001
		Married	0.88 (0.82-0.95)	<0.001	0.66 (0.59-0.73)	<0.001
	Government health insurance	Other	1.24 (0.73-2.11)	0.419	2.23 (1.82-2.75)	<0.001
		Married	1.11 (0.86-1.43)	0.418	1.71 (1.48-1.97)	<0.001
	Other health insurance	Other	0.57 (0.31-1.06)	0.076	1.19 (0.96-1.48)	0.117
		Married	1.02 (0.78-1.33)	0.873	0.92 (0.79-1.08)	0.328
	Medical expenditure	Other	1.37 (1.14-1.65)	<0.001	1.17 (1.04-1.32)	0.007
		Married	1.34 (1.24-1.45)	<0.001	1.21 (1.12-1.31)	<0.001

Physical–cognitive multimorbidity	Education	Other	0.49 (0.32-0.75)	<0.001	0.41 (0.33-0.52)	<0.001
		Married	0.43 (0.36-0.51)	<0.001	0.33 (0.28-0.39)	<0.001
	Household income	Other	0.84 (0.72-0.97)	0.021	0.78 (0.66-0.91)	0.002
		Married	0.87 (0.81-0.93)	<0.001	0.76 (0.68-0.84)	<0.001
	Household wealth	Other	0.99 (0.85-1.14)	0.848	0.65 (0.56-0.77)	<0.001
		Married	0.87 (0.81-0.94)	<0.001	0.71 (0.64-0.80)	<0.001
	Government health insurance	Other	0.80 (0.50-1.28)	0.356	2.75 (2.18-3.47)	<0.001
		Married	0.84 (0.66-1.07)	0.164	2.51 (2.14-2.95)	<0.001
	Other health insurance	Other	0.67 (0.37-1.22)	0.192	1.33 (1.05-1.69)	0.017
		Married	0.66 (0.49-0.90)	0.008	1.03 (0.87-1.23)	0.715
	Medical expenditure	Other	1.19 (0.98-1.43)	0.073	1.04 (0.91-1.18)	0.579
		Married	1.20 (1.11-1.31)	<0.001	1.16 (1.06-1.28)	0.002
Psychological–cognitive multimorbidity	Education	Other	0.26 (0.15-0.45)	<0.001	0.32 (0.23-0.46)	<0.001
		Married	0.33 (0.27-0.40)	<0.001	0.46 (0.35-0.59)	<0.001
	Household income	Other	0.75 (0.65-0.87)	<0.001	0.85 (0.63-1.14)	0.272
		Married	0.75 (0.70-0.80)	<0.001	0.61 (0.50-0.75)	<0.001
	Household wealth	Other	0.75 (0.64-0.86)	<0.001	0.50 (0.37-0.67)	<0.001
		Married	0.75 (0.70-0.81)	<0.001	0.60 (0.49-0.73)	<0.001
	Government health insurance	Other	0.70 (0.45-1.08)	0.109	0.79 (0.54-1.18)	0.249
		Married	0.76 (0.61-0.96)	0.021	0.66 (0.48-0.91)	0.010
	Other health insurance	Other	0.37 (0.19-0.72)	0.004	1.03 (0.69-1.52)	0.888
		Married	0.64 (0.46-0.87)	0.005	0.64 (0.47-0.86)	0.003
	Medical expenditure	Other	1.22 (1.01-1.47)	0.041	0.58 (0.46-0.73)	<0.001
		Married	1.18 (1.08-1.29)	<0.001	0.73 (0.61-0.87)	<0.001
Physical–psychological–cognitive multimorbidity	Education	Other	0.23 (0.14-0.37)	<0.001	0.29 (0.23-0.36)	<0.001
		Married	0.23 (0.19-0.27)	<0.001	0.24 (0.21-0.28)	<0.001
	Household income	Other	0.70 (0.61-0.81)	<0.001	0.59 (0.51-0.69)	<0.001
		Married	0.79 (0.74-0.84)	<0.001	0.64 (0.57-0.71)	<0.001

	Household wealth	Other	0.73 (0.64-0.84)	<0.001	0.46 (0.39-0.53)	<0.001
		Married	0.70 (0.66-0.75)	<0.001	0.49 (0.44-0.55)	<0.001
	Government health insurance	Other	0.77 (0.51-1.16)	0.210	2.95 (2.37-3.68)	<0.001
		Married	0.76 (0.61-0.93)	0.010	2.42 (2.07-2.85)	<0.001
	Other health insurance	Other	0.39 (0.22-0.69)	0.001	1.09 (0.87-1.36)	0.462
		Married	0.68 (0.52-0.89)	0.004	0.83 (0.70-0.98)	0.032
	Medical expenditure	Other	1.54 (1.30-1.81)	<0.001	1.07 (0.95-1.21)	0.259
		Married	1.39 (1.29-1.49)	<0.001	1.15 (1.05-1.26)	0.003

Table S19. Whether Drinking—stratified analysis of socioeconomic and health factors associated with multimorbidity outcomes

**Domain**	**Risk factors**	**Whether Drinking**	**China**		**The US**	
			**OR**	***P* value**	**OR**	***P* value**
Physical multimorbidity	Education	No	1.37 (1.12-1.68)	0.002	1.06 (0.93-1.21)	0.403
		Yes	1.01 (0.79-1.30)	0.927	0.85 (0.64-1.13)	0.268
	Household income	No	1.08 (0.96-1.21)	0.203	0.92 (0.84-1.00)	0.051
		Yes	0.97 (0.84-1.13)	0.688	1.00 (0.82-1.21)	0.970
	Household wealth	No	1.02 (0.90-1.15)	0.794	0.79 (0.72-0.87)	<0.001
		Yes	1.01 (0.87-1.18)	0.880	0.79 (0.65-0.97)	0.023
	Government health insurance	No	1.43 (0.89-2.29)	0.139	2.42 (2.12-2.77)	<0.001
		Yes	1.94 (0.94-4.00)	0.072	2.26 (1.70-3.00)	<0.001
	Other health insurance	No	1.52 (1.01-2.27)	0.042	1.31 (1.13-1.52)	<0.001
		Yes	0.91 (0.53-1.55)	0.728	1.42 (1.03-1.96)	0.031
	Medical expenditure	No	1.21 (1.06-1.39)	0.005	1.17 (1.09-1.27)	<0.001
		Yes	1.37 (1.16-1.61)	<0.001	1.42 (1.21-1.66)	<0.001
Psychological multimorbidity	Education	No	0.93 (0.72-1.22)	0.606	1.35 (1.03-1.78)	0.031
		Yes	0.87 (0.64-1.18)	0.373	0.99 (0.59-1.64)	0.954
	Household income	No	0.90 (0.79-1.03)	0.131	0.78 (0.62-0.97)	0.025

		Yes	0.94 (0.79-1.12)	0.503	1.04 (0.70-1.55)	0.855
	Household wealth	No	0.86 (0.75-0.99)	0.037	0.72 (0.58-0.90)	0.003
		Yes	0.87 (0.73-1.05)	0.140	0.81 (0.55-1.19)	0.285
	Government health insurance	No	0.94 (0.59-1.47)	0.773	0.76 (0.54-1.07)	0.114
		Yes	0.91 (0.50-1.66)	0.761	0.45 (0.21-0.97)	0.042
	Other health insurance	No	0.67 (0.36-1.24)	0.204	0.62 (0.44-0.87)	0.005
		Yes	0.67 (0.33-1.37)	0.273	0.68 (0.37-1.27)	0.228
	Medical expenditure	No	1.22 (1.03-1.45)	0.021	0.82 (0.67-1.00)	0.054
		Yes	1.38 (1.12-1.69)	0.003	0.92 (0.64-1.32)	0.651
Cognitive multimorbidity	Education	No	0.24 (0.15-0.37)	<0.001	0.23 (0.14-0.35)	<0.001
		Yes	0.26 (0.15-0.44)	<0.001	0.24 (0.09-0.62)	0.003
	Household income	No	0.71 (0.63-0.81)	<0.001	0.65 (0.45-0.96)	0.031
		Yes	0.76 (0.64-0.90)	0.001	1.04 (0.48-2.22)	0.928
	Household wealth	No	0.77 (0.68-0.87)	<0.001	0.69 (0.48-0.99)	0.044
		Yes	0.75 (0.63-0.89)	0.001	0.48 (0.22-1.05)	0.067
	Government health insurance	No	0.61 (0.42-0.89)	0.009	1.45 (0.88-2.39)	0.143
		Yes	0.49 (0.30-0.82)	0.006	1.06 (0.35-3.16)	0.920
	Other health insurance	No	0.58 (0.32-1.06)	0.075	0.80 (0.48-1.35)	0.406
		Yes	0.29 (0.10-0.84)	0.022	0.91 (0.29-2.79)	0.864
	Medical expenditure	No	0.94 (0.80-1.12)	0.512	0.63 (0.46-0.86)	0.004
		Yes	0.83 (0.65-1.07)	0.146	0.52 (0.25-1.08)	0.081
Physical–psychological multimorbidity	Education	No	0.86 (0.72-1.03)	0.111	0.82 (0.72-0.93)	0.003
		Yes	0.83 (0.66-1.05)	0.116	0.86 (0.65-1.13)	0.281
	Household income	No	0.89 (0.82-0.96)	0.002	0.77 (0.71-0.84)	<0.001
		Yes	1.01 (0.90-1.12)	0.895	0.83 (0.69-0.99)	0.043
	Household wealth	No	0.89 (0.82-0.96)	0.003	0.61 (0.56-0.67)	<0.001
		Yes	0.85 (0.76-0.95)	0.004	0.63 (0.52-0.76)	<0.001
	Government health	No	1.07 (0.81-1.41)	0.638	1.95 (1.71-2.22)	<0.001

	insurance	Yes	1.30 (0.85-1.97)	0.226	1.54 (1.17-2.02)	0.002
	Other health insurance	No	1.01 (0.74-1.38)	0.944	0.99 (0.86-1.14)	0.854
		Yes	0.88 (0.59-1.32)	0.542	1.02 (0.76-1.38)	0.876
	Medical expenditure	No	1.42 (1.29-1.56)	<0.001	1.18 (1.10-1.27)	<0.001
		Yes	1.22 (1.08-1.38)	0.002	1.26 (1.08-1.47)	0.003
Physical–cognitive multimorbidity	Education	No	0.39 (0.31-0.48)	<0.001	0.37 (0.32-0.42)	<0.001
		Yes	0.52 (0.41-0.66)	<0.001	0.31 (0.22-0.42)	<0.001
	Household income	No	0.85 (0.79-0.92)	<0.001	0.74 (0.67-0.82)	<0.001
		Yes	0.87 (0.78-0.97)	0.009	0.78 (0.63-0.97)	0.027
	Household wealth	No	0.88 (0.81-0.95)	0.002	0.68 (0.62-0.75)	<0.001
		Yes	0.89 (0.80-1.00)	0.051	0.69 (0.56-0.86)	0.001
	Government health insurance	No	0.80 (0.61-1.04)	0.095	2.63 (2.28-3.04)	<0.001
		Yes	0.91 (0.62-1.33)	0.625	2.13 (1.54-2.93)	<0.001
	Other health insurance	No	0.75 (0.54-1.06)	0.103	1.07 (0.92-1.25)	0.366
		Yes	0.58 (0.37-0.93)	0.022	1.39 (0.98-1.99)	0.067
	Medical expenditure	No	1.24 (1.13-1.37)	<0.001	1.09 (1.01-1.19)	0.037
		Yes	1.13 (1.00-1.29)	0.055	1.22 (1.01-1.47)	0.036
Psychological–cognitive multimorbidity	Education	No	0.31 (0.25-0.40)	<0.001	0.42 (0.33-0.53)	<0.001
		Yes	0.32 (0.24-0.42)	<0.001	0.34 (0.22-0.55)	<0.001
	Household income	No	0.70 (0.65-0.76)	<0.001	0.67 (0.56-0.81)	<0.001
		Yes	0.84 (0.75-0.93)	0.001	0.68 (0.49-0.96)	0.028
	Household wealth	No	0.72 (0.66-0.78)	<0.001	0.59 (0.49-0.72)	<0.001
		Yes	0.79 (0.71-0.88)	<0.001	0.44 (0.31-0.63)	<0.001
	Government health insurance	No	0.78 (0.61-1.01)	0.057	0.78 (0.59-1.02)	0.074
		Yes	0.68 (0.48-0.96)	0.027	0.49 (0.27-0.89)	0.020
	Other health insurance	No	0.60 (0.42-0.86)	0.006	0.70 (0.53-0.92)	0.010
		Yes	0.58 (0.36-0.93)	0.025	0.94 (0.57-1.57)	0.820
	Medical	No	1.26 (1.14-1.39)	<0.001	0.67 (0.57-0.78)	<0.001

	expenditure	Yes	1.09 (0.95-1.24)	0.216	0.72 (0.53-0.97)	0.031
Physical–psychological–cognitive multimorbidity	Education	No	0.21 (0.17-0.26)	<0.001	0.27 (0.23-0.31)	<0.001
		Yes	0.29 (0.22-0.37)	<0.001	0.24 (0.18-0.33)	<0.001
	Household income	No	0.74 (0.69-0.80)	<0.001	0.58 (0.53-0.64)	<0.001
		Yes	0.81 (0.73-0.89)	<0.001	0.61 (0.49-0.74)	<0.001
	Household wealth	No	0.69 (0.64-0.74)	<0.001	0.45 (0.40-0.49)	<0.001
		Yes	0.73 (0.65-0.81)	<0.001	0.51 (0.41-0.63)	<0.001
	Government health insurance	No	0.70 (0.55-0.87)	0.002	2.77 (2.40-3.19)	<0.001
		Yes	0.97 (0.69-1.36)	0.854	1.98 (1.46-2.68)	<0.001
	Other health insurance	No	0.65 (0.48-0.88)	0.005	0.92 (0.80-1.07)	0.277
		Yes	0.62 (0.41-0.95)	0.028	0.83 (0.60-1.14)	0.239
	Medical expenditure	No	1.48 (1.36-1.61)	<0.001	1.10 (1.01-1.19)	0.022
		Yes	1.29 (1.15-1.45)	<0.001	1.20 (1.01-1.42)	0.039

Table S20. Whether smoking—stratified analysis of socioeconomic and health factors associated with multimorbidity outcomes

**Domains**	**Risk factors**	**Whether smoking**	**China**		**The US**	
			**OR**	***P* value**	**OR**	***P* value**
Physical multimorbidity	Education	No	1.16 (0.97-1.39)	0.108	0.97 (0.85-1.11)	0.637
		Yes	1.26 (0.93-1.70)	0.130	1.37 (0.98-1.90)	0.062
	Household income	No	1.02 (0.92-1.14)	0.661	0.92 (0.84-1.00)	0.046
		Yes	1.05 (0.89-1.25)	0.538	1.01 (0.82-1.26)	0.910
	Household wealth	No	0.96 (0.86-1.07)	0.468	0.80 (0.73-0.88)	<0.001
		Yes	1.11 (0.93-1.32)	0.248	0.70 (0.55-0.87)	0.002
	Government health insurance	No	1.52 (0.97-2.40)	0.069	2.35 (2.07-2.68)	<0.001
		Yes	1.61 (0.74-3.49)	0.233	2.77 (2.01-3.82)	<0.001
	Other health insurance	No	1.15 (0.79-1.67)	0.479	1.29 (1.11-1.49)	<0.001
		Yes	1.38 (0.75-2.51)	0.299	1.59 (1.14-2.21)	0.006

	Medical expenditure	No	1.27 (1.12-1.43)	<0.001	1.17 (1.09-1.26)	<0.001
		Yes	1.30 (1.07-1.58)	0.009	1.47 (1.23-1.76)	<0.001
Psychological multimorbidity	Education	No	0.81 (0.64-1.04)	0.097	1.22 (0.94-1.59)	0.136
		Yes	1.10 (0.76-1.59)	0.602	1.77 (0.94-3.33)	0.075
	Household income	No	0.93 (0.82-1.06)	0.292	0.79 (0.64-0.98)	0.031
		Yes	0.86 (0.71-1.04)	0.130	1.10 (0.70-1.72)	0.679
	Household wealth	No	0.89 (0.78-1.02)	0.100	0.82 (0.67-1.01)	0.059
		Yes	0.78 (0.64-0.95)	0.013	0.53 (0.33-0.86)	0.010
	Government health insurance	No	1.00 (0.65-1.54)	0.995	0.65 (0.46-0.90)	0.010
		Yes	0.77 (0.39-1.50)	0.437	0.93 (0.45-1.93)	0.849
	Other health insurance	No	0.68 (0.40-1.17)	0.161	0.62 (0.45-0.87)	0.005
		Yes	0.59 (0.23-1.46)	0.253	0.72 (0.37-1.38)	0.324
	Medical expenditure	No	1.33 (1.14-1.56)	<0.001	0.82 (0.67-0.99)	0.040
		Yes	1.15 (0.89-1.48)	0.282	1.01 (0.69-1.49)	0.948
Cognitive multimorbidity	Education	No	0.21 (0.13-0.31)	<0.001	0.23 (0.15-0.35)	<0.001
		Yes	0.34 (0.20-0.59)	<0.001	0.18 (0.06-0.56)	0.003
	Household income	No	0.72 (0.64-0.81)	<0.001	0.76 (0.53-1.10)	0.148
		Yes	0.75 (0.62-0.89)	0.001	0.32 (0.09-1.11)	0.073
	Household wealth	No	0.75 (0.67-0.85)	<0.001	0.62 (0.44-0.88)	0.008
		Yes	0.76 (0.63-0.91)	0.003	0.63 (0.25-1.62)	0.341
	Government health insurance	No	0.68 (0.47-0.98)	0.038	1.27 (0.78-2.08)	0.337
		Yes	0.39 (0.23-0.65)	<0.001	1.97 (0.61-6.32)	0.255
	Other health insurance	No	0.61 (0.35-1.05)	0.075	0.80 (0.48-1.34)	0.395
		Yes	0.10 (0.01-0.71)	0.022	0.95 (0.29-3.07)	0.932
	Medical expenditure	No	0.88 (0.75-1.05)	0.150	0.58 (0.42-0.79)	<0.001
		Yes	0.96 (0.74-1.23)	0.723	0.87 (0.42-1.81)	0.707
Physical–psychological	Education	No	0.80 (0.67-0.94)	0.007	0.79 (0.69-0.89)	<0.001
		Yes	0.88 (0.67-1.16)	0.379	1.35 (0.98-1.86)	0.069

multimorbidity	Household income	No	0.91 (0.84-0.98)	0.015	0.78 (0.72-0.85)	<0.001
		Yes	0.96 (0.85-1.07)	0.433	0.80 (0.65-0.98)	0.034
	Household wealth	No	0.88 (0.81-0.95)	0.001	0.65 (0.59-0.71)	<0.001
		Yes	0.84 (0.75-0.95)	0.004	0.50 (0.40-0.62)	<0.001
	Government health insurance	No	1.05 (0.80-1.37)	0.730	1.81 (1.59-2.05)	<0.001
		Yes	1.38 (0.87-2.18)	0.169	2.39 (1.77-3.23)	<0.001
	Other health insurance	No	0.96 (0.72-1.28)	0.781	1.00 (0.87-1.15)	0.972
		Yes	0.87 (0.54-1.41)	0.580	1.01 (0.75-1.36)	0.970
	Medical expenditure	No	1.35 (1.24-1.48)	<0.001	1.16 (1.08-1.25)	<0.001
		Yes	1.32 (1.15-1.51)	<0.001	1.47 (1.25-1.73)	<0.001
Physical–cognitive multimorbidity	Education	No	0.37 (0.31-0.45)	<0.001	0.34 (0.29-0.39)	<0.001
		Yes	0.56 (0.42-0.74)	<0.001	0.57 (0.40-0.80)	0.001
	Household income	No	0.85 (0.79-0.92)	<0.001	0.72 (0.65-0.79)	<0.001
		Yes	0.87 (0.78-0.97)	0.016	0.92 (0.72-1.16)	0.456
	Household wealth	No	0.88 (0.81-0.95)	0.001	0.70 (0.63-0.77)	<0.001
		Yes	0.88 (0.78-0.99)	0.031	0.54 (0.42-0.70)	<0.001
	Government health insurance	No	0.93 (0.72-1.21)	0.598	2.68 (2.33-3.10)	<0.001
		Yes	0.63 (0.43-0.93)	0.020	2.06 (1.46-2.91)	<0.001
	Other health insurance	No	0.67 (0.49-0.92)	0.014	1.08 (0.93-1.26)	0.307
		Yes	0.71 (0.42-1.19)	0.196	1.33 (0.94-1.88)	0.110
	Medical expenditure	No	1.28 (1.17-1.40)	<0.001	1.07 (0.99-1.16)	0.099
		Yes	1.01 (0.88-1.16)	0.900	1.34 (1.10-1.61)	0.003
Psychological–cognitive multimorbidity	Education	No	0.29 (0.23-0.36)	<0.001	0.37 (0.29-0.47)	<0.001
		Yes	0.35 (0.25-0.49)	<0.001	0.67 (0.42-1.07)	0.094
	Household income	No	0.75 (0.69-0.80)	<0.001	0.70 (0.58-0.85)	<0.001
		Yes	0.75 (0.67-0.83)	<0.001	0.67 (0.47-0.95)	0.024
	Household wealth	No	0.70 (0.65-0.76)	<0.001	0.55 (0.46-0.66)	<0.001
		Yes	0.81 (0.73-0.91)	<0.001	0.63 (0.44-0.92)	0.015

	Government health insurance	No	0.74 (0.58-0.95)	0.017	0.75 (0.57-1.00)	0.048
		Yes	0.74 (0.51-1.08)	0.120	0.65 (0.38-1.10)	0.108
	Other health insurance	No	0.55 (0.39-0.78)	<0.001	0.67 (0.51-0.88)	0.004
		Yes	0.63 (0.38-1.06)	0.081	1.17 (0.72-1.92)	0.521
	Medical expenditure	No	1.20 (1.09-1.33)	<0.001	0.67 (0.57-0.78)	<0.001
		Yes	1.18 (1.02-1.35)	0.024	0.73 (0.55-0.98)	0.036
Physical–psychological–cognitive multimorbidity	Education	No	0.19 (0.16-0.24)	<0.001	0.25 (0.22-0.29)	<0.001
		Yes	0.30 (0.22-0.41)	<0.001	0.39 (0.28-0.55)	<0.001
	Household income	No	0.75 (0.70-0.80)	<0.001	0.58 (0.53-0.64)	<0.001
		Yes	0.79 (0.72-0.88)	<0.001	0.62 (0.50-0.78)	<0.001
	Household wealth	No	0.69 (0.64-0.74)	<0.001	0.47 (0.43-0.52)	<0.001
		Yes	0.70 (0.63-0.78)	<0.001	0.41 (0.32-0.52)	<0.001
	Government health insurance	No	0.75 (0.60-0.93)	0.010	2.66 (2.31-3.06)	<0.001
		Yes	0.78 (0.55-1.12)	0.174	2.74 (2.01-3.75)	<0.001
	Other health insurance	No	0.60 (0.45-0.80)	<0.001	0.97 (0.83-1.12)	0.647
		Yes	0.67 (0.42-1.08)	0.100	0.77 (0.56-1.04)	0.087
	Medical expenditure	No	1.46 (1.34-1.58)	<0.001	1.10 (1.01-1.19)	0.021
		Yes	1.31 (1.16-1.48)	<0.001	1.27 (1.08-1.51)	0.005

Table S21. Frequency level of activity -stratified analysis of socioeconomic and health factors associated with multimorbidity outcomes

Domains	Risk factors	Frequency level of activity	China		The US	
			OR	*P* value	OR	*P* value
Physical multimorbidity	Education	Low	1.31 (1.06-1.63)	0.014	1.01 (0.80-1.27)	0.964
		High	1.13 (0.94-1.35)	0.209	1.01 (0.88-1.16)	0.934
	Household income	Low	1.12 (0.96-1.31)	0.153	0.92 (0.78-1.09)	0.319
		High	1.02 (0.92-1.14)	0.662	0.94 (0.86-1.02)	0.153
	Household wealth	Low	1.13 (0.96-1.32)	0.138	0.84 (0.71-0.99)	0.042

		High	0.97 (0.87-1.09)	0.600	0.81 (0.74-0.90)	<0.001
	Government health insurance	Low	1.89 (1.02-3.48)	0.043	2.25 (1.76-2.87)	<0.001
		High	1.56 (0.95-2.55)	0.078	2.39 (2.09-2.74)	<0.001
	Other health insurance	Low	1.50 (0.90-2.51)	0.120	1.47 (1.12-1.94)	0.005
		High	1.08 (0.74-1.58)	0.699	1.29 (1.11-1.50)	0.001
	Medical expenditure	Low	1.37 (1.15-1.63)	<0.001	1.27 (1.09-1.47)	0.002
		High	1.26 (1.11-1.43)	<0.001	1.21 (1.12-1.30)	<0.001
Psychological multimorbidity	Education	Low	0.74 (0.50-1.08)	0.116	1.42 (0.85-2.40)	0.184
		High	0.93 (0.75-1.17)	0.554	1.24 (0.95-1.61)	0.120
	Household income	Low	1.20 (0.96-1.50)	0.107	0.88 (0.58-1.34)	0.556
		High	0.86 (0.76-0.97)	0.016	0.82 (0.66-1.01)	0.063
	Household wealth	Low	0.88 (0.71-1.10)	0.269	0.55 (0.36-0.83)	0.005
		High	0.86 (0.76-0.98)	0.019	0.82 (0.66-1.01)	0.059
	Government health insurance	Low	0.70 (0.38-1.31)	0.267	1.14 (0.62-2.09)	0.673
		High	0.96 (0.62-1.47)	0.851	0.56 (0.39-0.79)	<0.001
	Other health insurance	Low	0.36 (0.11-1.21)	0.098	0.86 (0.45-1.62)	0.632
		High	0.75 (0.46-1.23)	0.259	0.57 (0.41-0.79)	<0.001
	Medical expenditure	Low	1.29 (0.99-1.69)	0.062	0.65 (0.44-0.96)	0.029
		High	1.27 (1.09-1.48)	0.002	0.88 (0.73-1.07)	0.193
Cognitive multimorbidity	Education	Low	0.39 (0.24-0.63)	<0.001	0.28 (0.10-0.76)	0.012
		High	0.19 (0.12-0.29)	<0.001	0.22 (0.14-0.34)	<0.001
	Household income	Low	0.59 (0.50-0.71)	<0.001	0.34 (0.13-0.93)	0.036
		High	0.78 (0.70-0.88)	<0.001	0.79 (0.55-1.13)	0.200
	Household wealth	Low	0.76 (0.64-0.91)	0.002	0.62 (0.25-1.56)	0.310
		High	0.76 (0.68-0.86)	<0.001	0.65 (0.45-0.92)	0.015
	Government health insurance	Low	0.55 (0.34-0.90)	0.016	1.19 (0.39-3.66)	0.761
		High	0.60 (0.42-0.86)	0.006	1.42 (0.87-2.32)	0.161
	Other health	Low	0.21 (0.05-0.90)	0.035	2.45 (0.72-8.34)	0.151

	insurance	High	0.52 (0.30-0.91)	0.023	0.70 (0.42-1.16)	0.164
	Medical expenditure	Low	0.98 (0.76-1.25)	0.847	0.44 (0.20-0.97)	0.042
		High	0.87 (0.74-1.02)	0.095	0.64 (0.47-0.87)	0.005
Physical–psychological multimorbidity	Education	Low	0.88 (0.72-1.07)	0.189	0.94 (0.74-1.19)	0.588
		High	0.79 (0.67-0.94)	0.008	0.83 (0.73-0.95)	0.008
	Household income	Low	1.01 (0.91-1.13)	0.795	0.77 (0.65-0.91)	0.002
		High	0.89 (0.83-0.96)	0.002	0.79 (0.72-0.86)	<0.001
	Household wealth	Low	0.96 (0.86-1.07)	0.429	0.64 (0.54-0.76)	<0.001
		High	0.84 (0.78-0.91)	<0.001	0.65 (0.59-0.71)	<0.001
	Government health insurance	Low	1.37 (0.94-1.99)	0.098	2.16 (1.70-2.75)	<0.001
		High	1.06 (0.80-1.40)	0.683	1.72 (1.50-1.96)	<0.001
	Other health insurance	Low	1.13 (0.74-1.71)	0.572	1.01 (0.78-1.30)	0.955
		High	0.86 (0.65-1.15)	0.305	0.99 (0.86-1.15)	0.934
	Medical expenditure	Low	1.49 (1.31-1.70)	<0.001	1.21 (1.05-1.39)	0.010
		High	1.31 (1.20-1.43)	<0.001	1.19 (1.11-1.29)	<0.001
Physical–cognitive multimorbidity	Education	Low	0.53 (0.42-0.66)	<0.001	0.46 (0.37-0.59)	<0.001
		High	0.39 (0.32-0.47)	<0.001	0.36 (0.31-0.41)	<0.001
	Household income	Low	0.86 (0.77-0.96)	0.006	0.69 (0.58-0.82)	<0.001
		High	0.85 (0.79-0.92)	<0.001	0.75 (0.68-0.83)	<0.001
	Household wealth	Low	0.92 (0.83-1.03)	0.142	0.78 (0.65-0.93)	0.006
		High	0.87 (0.80-0.94)	<0.001	0.69 (0.62-0.76)	<0.001
	Government health insurance	Low	0.95 (0.67-1.34)	0.758	2.97 (2.28-3.86)	<0.001
		High	0.79 (0.61-1.03)	0.086	2.44 (2.10-2.83)	<0.001
	Other health insurance	Low	0.72 (0.45-1.14)	0.162	1.18 (0.89-1.55)	0.252
		High	0.66 (0.48-0.91)	0.011	1.11 (0.95-1.31)	0.188
	Medical expenditure	Low	1.38 (1.21-1.57)	<0.001	1.07 (0.91-1.25)	0.408
		High	1.13 (1.03-1.24)	0.011	1.13 (1.04-1.23)	0.004

Psychological–cognitive multimorbidity	Education	Low	0.34 (0.26-0.46)	<0.001	0.39 (0.26-0.59)	<0.001
		High	0.30 (0.24-0.37)	<0.001	0.40 (0.31-0.50)	<0.001
	Household income	Low	0.70 (0.62-0.78)	<0.001	0.60 (0.42-0.85)	0.004
		High	0.77 (0.72-0.83)	<0.001	0.69 (0.58-0.83)	<0.001
	Household wealth	Low	0.76 (0.68-0.85)	<0.001	0.74 (0.52-1.04)	0.087
		High	0.73 (0.68-0.79)	<0.001	0.53 (0.44-0.63)	<0.001
	Government health insurance	Low	0.64 (0.46-0.90)	0.009	0.57 (0.35-0.93)	0.026
		High	0.79 (0.62-1.01)	0.060	0.71 (0.54-0.94)	0.015
	Other health insurance	Low	0.65 (0.38-1.10)	0.109	0.92 (0.57-1.50)	0.746
		High	0.54 (0.39-0.75)	<0.001	0.73 (0.56-0.96)	0.022
	Medical expenditure	Low	1.20 (1.04-1.39)	0.012	0.55 (0.40-0.74)	<0.001
		High	1.18 (1.08-1.30)	<0.001	0.68 (0.58-0.79)	<0.001
Physical–psychological–cognitive multimorbidity	Education	Low	0.31 (0.24-0.39)	<0.001	0.29 (0.22-0.37)	<0.001
		High	0.18 (0.15-0.22)	<0.001	0.27 (0.24-0.32)	<0.001
	Household income	Low	0.79 (0.71-0.87)	<0.001	0.58 (0.49-0.69)	<0.001
		High	0.75 (0.70-0.80)	<0.001	0.58 (0.53-0.64)	<0.001
	Household wealth	Low	0.73 (0.66-0.81)	<0.001	0.49 (0.41-0.59)	<0.001
		High	0.68 (0.64-0.73)	<0.001	0.48 (0.44-0.54)	<0.001
	Government health insurance	Low	0.86 (0.64-1.17)	0.345	2.46 (1.91-3.16)	<0.001
		High	0.72 (0.57-0.91)	0.005	2.57 (2.22-2.97)	<0.001
	Other health insurance	Low	0.99 (0.66-1.48)	0.955	0.85 (0.66-1.11)	0.231
		High	0.47 (0.35-0.63)	<0.001	0.93 (0.80-1.09)	0.378
	Medical expenditure	Low	1.54 (1.37-1.74)	<0.001	1.09 (0.94-1.26)	0.256
		High	1.36 (1.26-1.48)	<0.001	1.13 (1.04-1.23)	0.004

Table S22. Adjusted associations of multimorbidity patterns with ADL limitations in China and the US

Risk factors	China HR and 95% CI	China *P* value	The US HR and 95% CI	The US *P* value
**Multimorbidity**				
None	Ref		Ref	
Physical multimorbidity	1.795 (1.342-2.401)	<0.001	2.123 (1.626-2.772)	<0.001
Psychological multimorbidity	1.640 (1.175-2.288)	0.004	1.368 (0.639-2.929)	0.419
Cognitive multimorbidity	1.134 (0.823-1.564)	0.441	1.179 (0.287-4.846)	0.820
Physical–psychological multimorbidity	2.605 (2.182-3.110)	<0.001	3.569 (2.781-4.580)	<0.001
Physical–cognitive multimorbidity	1.958 (1.629-2.354)	<0.001	2.202 (1.655-2.932)	<0.001
Psychological–cognitive multimorbidity	1.647 (1.369-1.982)	<0.001	2.245 (1.377-3.658)	0.001
Physical–psychological–cognitive multimorbidity	2.971 (2.539-3.476)	<0.001	4.151 (3.207-5.373)	<0.001
**Sex**				
Male	Ref		Ref	
Female	1.420 (1.273-1.586)	<0.001	1.036 (0.919-1.168)	0.561
**Older age**	1.031 (1.026-1.037)	<0.001	1.023 (1.013-1.033)	<0.001
Area				
Urban	Ref		Ref	
Rural	1.124 (1.019-1.241)	0.019	1.064 (0.934-1.212)	0.353
**More household members**	1.041 (1.016-1.067)	0.001	1.015 (0.969-1.063)	0.535
**Marital status**				
Unmarried	Ref		Ref	
Married	0.930 (0.839-1.032)	0.171	1.066 (0.931-1.222)	0.353
**Higher BMI**	1.022 (1.010-1.034)	<0.001	1.037 (1.027-1.047)	<0.001
**Drinking**				
No	Ref		Ref	
Yes	0.918 (0.825-1.022)	0.116	0.969 (0.822-1.142)	0.705
**Smoking**				
No	Ref		Ref	
Yes	1.002 (0.893-1.123)	0.978	1.423 (1.211-1.671)	<0.001

**Activity**				
No	Ref		Ref	
Yes	0.789 (0.723-0.860)	<0.001	0.723 (0.639-0.817)	<0.001
**Education**				
Less than lower secondary	Ref		Ref	
Upper secondary	0.800 (0.668-0.959)	0.016	0.978 (0.835-1.146)	0.786
Tertiary	0.588 (0.338-1.025)	0.061	0.947 (0.766-1.170)	0.612
**Household income**				
Low	Ref		Ref	
Middle	0.899 (0.812-0.995)	0.039	0.762 (0.659-0.881)	<0.001
High	0.793 (0.704-0.894)	<0.001	0.698 (0.573-0.849)	<0.001
**Household wealth**				
Low	Ref		Ref	
Middle	0.894 (0.807-0.991)	0.033	0.801 (0.694-0.924)	0.002
High	0.742 (0.660-0.834)	<0.001	0.642 (0.533-0.772)	<0.001
**Government health insurance**				
No	Ref		Ref	
Yes	0.994 (0.832-1.188)	0.946	1.382 (1.190-1.604)	<0.001
**Other health insurance**				
No	Ref		Ref	
Yes	1.000 (0.765-1.307)	0.999	0.848 (0.745-0.964)	0.012
**Medical expenditure**				
None	Ref		Ref	
Low	1.004 (0.857-1.177)	0.957	0.974 (0.824-1.150)	0.755
High	1.134 (0.971-1.325)	0.111	0.909 (0.769-1.074)	0.264

Table S23. Adjusted associations of multimorbidity patterns with IADL limitations in China and the US

	China		The US	
**Risk factors**	**HR and 95% CI**	***P* value**	**HR and 95% CI**	***P* value**
**Multimorbidity**				
None	Ref		Ref	
Physical multimorbidity	1.396 (1.033-1.886)	0.030	1.597 (1.189-2.146)	0.002
Psychological multimorbidity	1.400 (0.981-1.998)	0.063	2.686 (1.450-4.976)	0.002
Cognitive multimorbidity	1.670 (1.256-2.221)	<0.001	4.064 (1.737-9.510)	0.001
Physical–psychological multimorbidity	1.770 (1.475-2.123)	<0.001	3.345 (2.557-4.378)	<0.001
Physical–cognitive multimorbidity	1.730 (1.436-2.086)	<0.001	2.138 (1.572-2.907)	<0.001
Psychological–cognitive multimorbidity	1.869 (1.565-2.234)	<0.001	2.356 (1.415-3.925)	<0.001
Physical–psychological–cognitive multimorbidity	2.597 (2.226-3.030)	<0.001	3.886 (2.933-5.149)	<0.001
**Sex**				
Male	Ref		Ref	
Female	1.654 (1.484-1.844)	<0.001	0.964 (0.849-1.094)	0.569
**Older age**	1.041 (1.035-1.048)	<0.001	1.025 (1.014-1.035)	<0.001
Area				
Urban	Ref		Ref	
Rural	1.212 (1.097-1.339)	<0.001	1.005 (0.873-1.156)	0.949
**More household members**	1.019 (0.994-1.045)	0.132	1.018 (0.978-1.061)	0.382
**Marital status**				
Unmarried	Ref		Ref	
Married	1.095 (0.981-1.222)	0.106	1.056 (0.916-1.217)	0.453
**Higher BMI**	0.998 (0.985-1.011)	0.737	1.010 (0.999-1.021)	0.083
**Drinking**				
No	Ref		Ref	
Yes	0.877 (0.789-0.975)	0.015	0.947 (0.790-1.135)	0.558
**Smoking**				
No	Ref		Ref	
Yes	1.040 (0.931-1.161)	0.490	1.434 (1.214-1.695)	<0.001

**Activity**				
No	Ref		Ref	
Yes	0.834 (0.763-0.912)	<0.001	0.697 (0.613-0.792)	<0.001
**Education**				
Less than lower secondary	Ref		Ref	
Upper secondary	0.660 (0.544-0.802)	<0.001	0.928 (0.790-1.090)	0.361
Tertiary	0.557 (0.32-0.967)	0.038	0.838 (0.667-1.052)	0.127
**Household income**				
Low	Ref		Ref	
Middle	0.873 (0.788-0.967)	0.009	0.809 (0.693-0.945)	0.007
High	0.656 (0.581-0.741)	<0.001	0.544 (0.434-0.682)	<0.001
**Household wealth**				
Low	Ref		Ref	
Middle	0.975 (0.880-1.081)	0.632	0.671 (0.577-0.782)	<0.001
High	0.741 (0.658-0.835)	<0.001	0.569 (0.465-0.695)	<0.001
**Government health insurance**				
No	Ref		Ref	
Yes	0.866 (0.728-1.030)	0.104	1.391 (1.186-1.630)	<0.001
**Other health insurance**				
No	Ref		Ref	
Yes	0.785 (0.587-1.050)	0.102	0.846 (0.739-0.968)	0.015
**Medical expenditure**				
None	Ref		Ref	
Low	1.085 (0.943-1.249)	0.252	1.055 (0.881-1.263)	0.561
High	Ref		Ref	

Table S24. Associations of socioeconomic factors and health factors with multimorbidity patterns in China based on natural cohorts

Risk factors	OR (95% CI)	P

**Physical multimorbidity**			
Education	Less than lower secondary	Ref	
	Upper secondary	1.16 (0.98-1.38)	0.089
	Tertiary	2.52 (1.78-3.57)	<0.001
Household income	Low	Ref	
	Middle	0.89 (0.74-1.08)	0.233
	High	0.98 (0.82-1.17)	0.813
Household wealth	Low	Ref	
	Middle	0.96 (0.79-1.17)	0.716
	High	1.16 (0.96-1.41)	0.123
Government health insurance	No	Ref	
	Yes	1.57 (1.02-2.42)	0.041
Other health insurance	No	Ref	
	Yes	1.13 (0.84-1.53)	0.417
Medical expenditure	None	Ref	
	Low	1.45 (1.16-1.81)	0.001
	High	2.56 (2.03-3.23)	<0.001
**Psychological multimorbidity**			
Education	Less than lower secondary	Ref	
	Upper secondary	1.00 (0.78-1.29)	0.991
	Tertiary	0.55 (0.22-1.38)	0.203
Household income	Low	Ref	
	Middle	1.15 (0.90-1.46)	0.259
	High	1.00 (0.79-1.28)	0.972
Household wealth	Low	Ref	

	Middle	0.65 (0.52-0.82)	<0.001
	High	0.49 (0.38-0.62)	<0.001
Government health insurance	No	Ref	
	Yes	0.88 (0.57-1.36)	0.567
Other health insurance	No	Ref	
	Yes	1.29 (0.85-1.95)	0.237
Medical expenditure	None	Ref	
	Low	1.27 (0.93-1.74)	0.133
	High	1.02 (0.68-1.54)	0.906
**Cognitive multimorbidity**			
Education	Less than lower secondary	Ref	
	Upper secondary	0.22 (0.13-0.36)	<0.001
	Tertiary	0.00 (0.00-Inf)	0.898
Household income	Low	Ref	
	Middle	0.72 (0.58-0.91)	0.005
	High	0.43 (0.33-0.56)	<0.001
Household wealth	Low	Ref	
	Middle	0.77 (0.61-0.97)	0.026
	High	0.55 (0.42-0.71)	<0.001
Government health insurance	No	Ref	
	Yes	0.53 (0.36-0.79)	0.001
Other health insurance	No	Ref	
	Yes	0.44 (0.21-0.90)	0.024
Medical expenditure	None	Ref	
	Low	0.82 (0.57-1.18)	0.287

	High	0.89 (0.55-1.42)	0.614
**Physical–psychological multimorbidity**			
Education	Less than lower secondary	Ref	
	Upper secondary	0.83 (0.73-0.94)	0.002
	Tertiary	1.11 (0.82-1.51)	0.497
Household income	Low	Ref	
	Middle	1.01 (0.90-1.14)	0.811
	High	0.83 (0.74-0.93)	0.001
Household wealth	Low	Ref	
	Middle	0.68 (0.60-0.76)	<0.001
	High	0.67 (0.60-0.75)	<0.001
Government health insurance	No	Ref	
	Yes	1.04 (0.82-1.31)	0.750
Other health insurance	No	Ref	
	Yes	0.91 (0.73-1.12)	0.369
Medical expenditure	None	Ref	
	Low	1.72 (1.48-1.99)	<0.001
	High	3.11 (2.64-3.67)	<0.001
**Physical–cognitive multimorbidity**			
Education	Less than lower secondary	Ref	
	Upper secondary	0.37 (0.31-0.44)	<0.001
	Tertiary	0.60 (0.40-0.90)	0.014
Household income	Low	Ref	
	Middle	0.92 (0.81-1.04)	0.170
	High	0.70 (0.62-0.79)	<0.001

Household wealth	Low	Ref	
	Middle	0.71 (0.63-0.81)	<0.001
	High	0.68 (0.60-0.77)	<0.001
Government health insurance	No	Ref	
	Yes	0.77 (0.61-0.97)	0.025
Other health insurance	No	Ref	
	Yes	0.68 (0.53-0.89)	0.005
Medical expenditure	None	Ref	
	Low	1.35 (1.15-1.60)	<0.001
	High	1.98 (1.64-2.39)	<0.001
**Psychological–cognitive multimorbidity**			
Education	Less than lower secondary	Ref	
	Upper secondary	0.24 (0.19-0.29)	<0.001
	Tertiary	0.20 (0.10-0.40)	<0.001
Household income	Low	Ref	
	Middle	0.85 (0.75-0.95)	0.006
	High	0.55 (0.49-0.63)	<0.001
Household wealth	Low	Ref	
	Middle	0.65 (0.57-0.73)	<0.001
	High	0.46 (0.40-0.52)	<0.001
Government health insurance	No	Ref	
	Yes	0.68 (0.54-0.85)	<0.001
Other health insurance	No	Ref	
	Yes	0.64 (0.48-0.85)	0.002
Medical expenditure	None	Ref	

	Low	1.01 (0.85-1.21)	0.883
	High	1.42 (1.16-1.75)	<0.001
**Physical–psychological–cognitive multimorbidity**			
Education	Less than lower secondary	Ref	
	Upper secondary	0.22 (0.20-0.26)	<0.001
	Tertiary	0.26 (0.18-0.39)	<0.001
Household income	Low	Ref	
	Middle	0.82 (0.74-0.90)	<0.001
	High	0.51 (0.46-0.56)	<0.001
Household wealth	Low	Ref	
	Middle	0.55 (0.50-0.60)	<0.001
	High	0.35 (0.32-0.39)	<0.001
Government health insurance	No	Ref	
	Yes	0.72 (0.60-0.87)	<0.001
Other health insurance	No	Ref	
	Yes	0.61 (0.50-0.74)	<0.001
Medical expenditure	None	Ref	
	Low	1.91 (1.68-2.17)	<0.001
	High	3.40 (2.93-3.96)	<0.001

Reference domain: non-multimorbidity condition

Table S25. Associations of socioeconomic factors and health factors with multimorbidity patterns in the US based on natural cohorts

Risk factors	OR (95% CI)	P

**Physical multimorbidity**			
Education	Less than lower secondary	Ref	
	Upper secondary	1.16 (0.98-1.38)	0.089
	Tertiary	2.52 (1.78-3.57)	<0.001
Household income	Low	Ref	
	Middle	0.89 (0.74-1.08)	0.233
	High	0.98 (0.82-1.17)	0.813
Household wealth	Low	Ref	
	Middle	0.96 (0.79-1.17)	0.716
	High	1.16 (0.96-1.41)	0.123
Government health insurance	No	Ref	
	Yes	1.57 (1.02-2.42)	0.041
Other health insurance	No	Ref	
	Yes	1.13 (0.84-1.53)	0.417
Medical expenditure	None	Ref	
	Low	1.45 (1.16-1.81)	0.001
	High	2.56 (2.03-3.23)	<0.001
**Psychological multimorbidity**			
Education	Less than lower secondary	Ref	
	Upper secondary	1.00 (0.78-1.29)	0.991
	Tertiary	0.55 (0.22-1.38)	0.203
Household income	Low	Ref	
	Middle	1.15 (0.90-1.46)	0.259
	High	1.00 (0.79-1.28)	0.972
Household wealth	Low	Ref	

	Middle	0.65 (0.52-0.82)	<0.001
	High	0.49 (0.38-0.62)	<0.001
Government health insurance	No	Ref	
	Yes	0.88 (0.57-1.36)	0.567
Other health insurance	No	Ref	
	Yes	1.29 (0.85-1.95)	0.237
Medical expenditure	None	Ref	
	Low	1.27 (0.93-1.74)	0.133
	High	1.02 (0.68-1.54)	0.906
**Cognitive multimorbidity**			
Education	Less than lower secondary	Ref	
	Upper secondary	0.22 (0.13-0.36)	<0.001
	Tertiary	0.00 (0.00-Inf)	0.898
Household income	Low	Ref	
	Middle	0.72 (0.58-0.91)	0.005
	High	0.43 (0.33-0.56)	<0.001
Household wealth	Low	Ref	
	Middle	0.77 (0.61-0.97)	0.026
	High	0.55 (0.42-0.71)	<0.001
Government health insurance	No	Ref	
	Yes	0.53 (0.36-0.79)	0.001
Other health insurance	No	Ref	
	Yes	0.44 (0.21-0.90)	0.024
Medical expenditure	None	Ref	
	Low	0.82 (0.57-1.18)	0.287

	High	0.89 (0.55-1.42)	0.614
**Physical–psychological multimorbidity**			
Education	Less than lower secondary	Ref	
	Upper secondary	0.83 (0.73-0.94)	0.002
	Tertiary	1.11 (0.82-1.51)	0.497
Household income	Low	Ref	
	Middle	1.01 (0.90-1.14)	0.811
	High	0.83 (0.74-0.93)	0.001
Household wealth	Low	Ref	
	Middle	0.68 (0.60-0.76)	<0.001
	High	0.67 (0.60-0.75)	<0.001
Government health insurance	No	Ref	
	Yes	1.04 (0.82-1.31)	0.750
Other health insurance	No	Ref	
	Yes	0.91 (0.73-1.12)	0.369
Medical expenditure	None	Ref	
	Low	1.72 (1.48-1.99)	<0.001
	High	3.11 (2.64-3.67)	<0.001
**Physical–cognitive multimorbidity**			
Education	Less than lower secondary	Ref	
	Upper secondary	0.37 (0.31-0.44)	<0.001
	Tertiary	0.60 (0.40-0.90)	0.014
Household income	Low	Ref	
	Middle	0.92 (0.81-1.04)	0.170
	High	0.70 (0.62-0.79)	<0.001

Household wealth	Low	Ref	
	Middle	0.71 (0.63-0.81)	<0.001
	High	0.68 (0.60-0.77)	<0.001
Government health insurance	No	Ref	
	Yes	0.77 (0.61-0.97)	0.025
Other health insurance	No	Ref	
	Yes	0.68 (0.53-0.89)	0.005
Medical expenditure	None	Ref	
	Low	1.35 (1.15-1.60)	<0.001
	High	1.98 (1.64-2.39)	<0.001
**Psychological–cognitive multimorbidity**			
Education	Less than lower secondary	Ref	
	Upper secondary	0.24 (0.19-0.29)	<0.001
	Tertiary	0.20 (0.10-0.40)	<0.001
Household income	Low	Ref	
	Middle	0.85 (0.75-0.95)	0.006
	High	0.55 (0.49-0.63)	<0.001
Household wealth	Low	Ref	
	Middle	0.65 (0.57-0.73)	<0.001
	High	0.46 (0.40-0.52)	<0.001
Government health insurance	No	Ref	
	Yes	0.68 (0.54-0.85)	<0.001
Other health insurance	No	Ref	
	Yes	0.64 (0.48-0.85)	0.002
Medical expenditure	None	Ref	

	Low	1.01 (0.85-1.21)	0.883
	High	1.42 (1.16-1.75)	<0.001
**Physical–psychological–cognitive multimorbidity**			
Education	Less than lower secondary	Ref	
	Upper secondary	0.22 (0.20-0.26)	<0.001
	Tertiary	0.26 (0.18-0.39)	<0.001
Household income	Low	Ref	
	Middle	0.82 (0.74-0.90)	<0.001
	High	0.51 (0.46-0.56)	<0.001
Household wealth	Low	Ref	
	Middle	0.55 (0.50-0.60)	<0.001
	High	0.35 (0.32-0.39)	<0.001
Government health insurance	No	Ref	
	Yes	0.72 (0.60-0.87)	<0.001
Other health insurance	No	Ref	
	Yes	0.61 (0.50-0.74)	<0.001
Medical expenditure	None	Ref	
	Low	1.91 (1.68-2.17)	<0.001
	High	3.40 (2.93-3.96)	<0.001

Reference domain: non-multimorbidity condition

Table S26. Adjusted associations of multimorbidity patterns with ADL limitations in China and the US based on natural cohorts

	China		The US	
**Risk factors**	**HR and 95% CI**	***P* value**	**HR and 95% CI**	***P* value**

**Multimorbidity**				
None	Ref		Ref	
Physical multimorbidity	1.720 (0.995-2.973)	0.052	2.380 (1.877-3.019)	<0.001
Psychological multimorbidity	1.933 (1.048-3.564)	0.035	3.022 (1.373-6.650)	0.006
Cognitive multimorbidity	0.953 (0.457-1.988)	0.898	3.474 (1.914-6.305)	<0.001
Physical–psychological multimorbidity	2.940 (2.117-4.084)	<0.001	4.174 (3.313-5.260)	<0.001
Physical–cognitive multimorbidity	2.290 (1.628-3.220)	<0.001	2.952 (2.337-3.730)	<0.001
Psychological–cognitive multimorbidity	1.770 (1.244-2.517)	0.001	2.789 (1.900-4.094)	<0.001
Physical–psychological–cognitive multimorbidity	3.290 (2.446-4.425)	<0.001	5.315 (4.234-6.672)	<0.001
**Sex**				
Male	Ref		Ref	
Female	1.380 (1.135-1.677)	0.001	1.044 (0.960-1.136)	0.310
**Older age**	1.036 (1.027-1.045)	<0.001	1.048 (1.042-1.054)	<0.001
**Area**				
Urban	Ref		Ref	
Rural	1.123 (0.942-1.339)	0.194	0.958 (0.879-1.044)	0.324
**More household members**	1.095 (1.044-1.149)	<0.001	1.027 (0.992-1.063)	0.135
**Marital status**				
Unmarried	Ref		Ref	
Married	0.965 (0.793-1.175)	0.725	0.903 (0.822-0.992)	0.033
**Higher BMI**	1.034 (1.012-1.055)	0.002	1.033 (1.025-1.040)	<0.001
**Drinking**				
No	Ref		Ref	
Yes	1.049 (0.867-1.270)	0.622	0.891 (0.768-1.034)	0.128
**Smoking**				
No	Ref		Ref	
Yes	1.051 (0.850-1.301)	0.645	1.396 (1.233-1.581)	<0.001
**Activity**				
No	Ref		Ref	

Yes	0.813 (0.698-0.946)	0.008	0.696 (0.642-0.754)	<0.001
**Education**				
Less than lower secondary	Ref		Ref	
Upper secondary	0.663 (0.464-0.949)	0.025	0.919 (0.831-1.017)	0.101
Tertiary	0.477 (0.149-1.526)	0.212	0.925 (0.804-1.063)	0.272
**Household income**				
Low	Ref		Ref	
Middle	0.910 (0.764-1.085)	0.293	0.897 (0.811-0.991)	0.032
High	0.745 (0.602-0.923)	0.007	0.944 (0.829-1.075)	0.386
**Household wealth**				
Low	Ref		Ref	
Middle	0.838 (0.702-1.000)	0.050	0.851 (0.771-0.940)	0.001
High	0.731 (0.595-0.897)	0.003	0.730 (0.648-0.824)	<0.001
**Government health insurance**				
No	Ref		Ref	
Yes	0.781 (0.580-1.051)	0.103	1.167 (1.010-1.349)	0.037
**Other health insurance**				
No	Ref		Ref	
Yes	0.734 (0.430-1.253)	0.258	0.892 (0.819-0.971)	0.008
**Medical expenditure**				
None	Ref		Ref	
Low	1.067 (0.798-1.427)	0.662	0.810 (0.718-0.912)	<0.001
High	1.400 (1.044-1.879)	0.025	1.060 (0.941-1.194)	0.339

Table S27. Adjusted associations of multimorbidity patterns with IADL limitations in China and the US based on natural cohorts

	China		The US	
**Risk factors**	**HR and 95% CI**	***P* value**	**HR and 95% CI**	***P* value**

**Multimorbidity**				
None	Ref		Ref	
Physical multimorbidity	0.714 (0.348-1.462)	0.357	1.752 (1.378-2.227)	<0.001
Psychological multimorbidity	1.394 (0.665-2.923)	0.379	1.475 (0.460-4.724)	0.513
Cognitive multimorbidity	0.638 (0.252-1.618)	0.344	5.309 (3.141-8.973)	<0.001
Physical–psychological multimorbidity	1.496 (1.044-2.143)	0.028	3.274 (2.599-4.124)	<0.001
Physical–cognitive multimorbidity	1.489 (1.021-2.172)	0.039	2.933 (2.325-3.701)	<0.001
Psychological–cognitive multimorbidity	1.971 (1.385-2.804)	<0.001	3.972 (2.810-5.613)	<0.001
Physical–psychological–cognitive multimorbidity	2.387 (1.767-3.225)	<0.001	4.839 (3.855-6.074)	<0.001
**Sex**				
Male	Ref		Ref	
Female	1.654 (1.301-2.101)	<0.001	0.890 (0.818-0.968)	0.007
**Older age**	1.046 (1.034-1.057)	<0.001	1.055 (1.048-1.061)	<0.001
**Area**				
Urban	Ref		Ref	
Rural	1.177 (0.949-1.459)	0.138	1.030 (0.946-1.121)	0.5
**More household members**	1.041 (0.980-1.106)	0.196	1.045 (1.013-1.078)	0.006
**Marital status**				
Unmarried	Ref		Ref	
Married	0.946 (0.744-1.204)	0.654	0.970 (0.882-1.066)	0.521
**Higher BMI**	0.992 (0.965-1.019)	0.552	1.013 (1.005-1.021)	0.002
**Drinking**				
No	Ref		Ref	
Yes	0.914 (0.728-1.147)	0.438	0.803 (0.685-0.940)	0.006
**Smoking**				
No	Ref		Ref	
Yes	1.288 (0.998-1.662)	0.051	1.381 (1.215-1.570)	<0.001
**Activity**				
No	Ref		Ref	

Yes	0.895 (0.743-1.079)	0.245	0.643 (0.593-0.697)	<0.001
**Education**				
Less than lower secondary	Ref		Ref	
Upper secondary	0.410 (0.247-0.681)	<0.001	0.997 (0.902-1.102)	0.949
Tertiary	1.118 (0.465-2.689)	0.803	0.985 (0.854-1.136)	0.836
**Household income**				
Low	Ref		Ref	
Middle	0.961 (0.780-1.184)	0.711	0.945 (0.855-1.045)	0.273
High	0.710 (0.553-0.912)	0.007	0.794 (0.691-0.912)	0.001
**Household wealth**				
Low	Ref		Ref	
Middle	1.016 (0.816-1.266)	0.884	0.816 (0.739-0.902)	<0.001
High	1.066 (0.836-1.360)	0.605	0.719 (0.638-0.811)	<0.001
**Government health insurance**				
No	Ref		Ref	
Yes	1.042 (0.637-1.702)	0.871	1.157 (0.993-1.348)	0.061
**Other health insurance**				
No	Ref		Ref	
Yes	0.736 (0.378-1.430)	0.365	0.840 (0.771-0.914)	<0.001
**Medical expenditure**				
None	Ref		Ref	
Low	1.052 (0.765-1.447)	0.756	0.865 (0.766-0.976)	0.019
High	1.023 (0.700-1.496)	0.907	1.047 (0.926-1.183)	0.465

Supplementary Figures

Figure S1. Flowchart showing the selection of participants

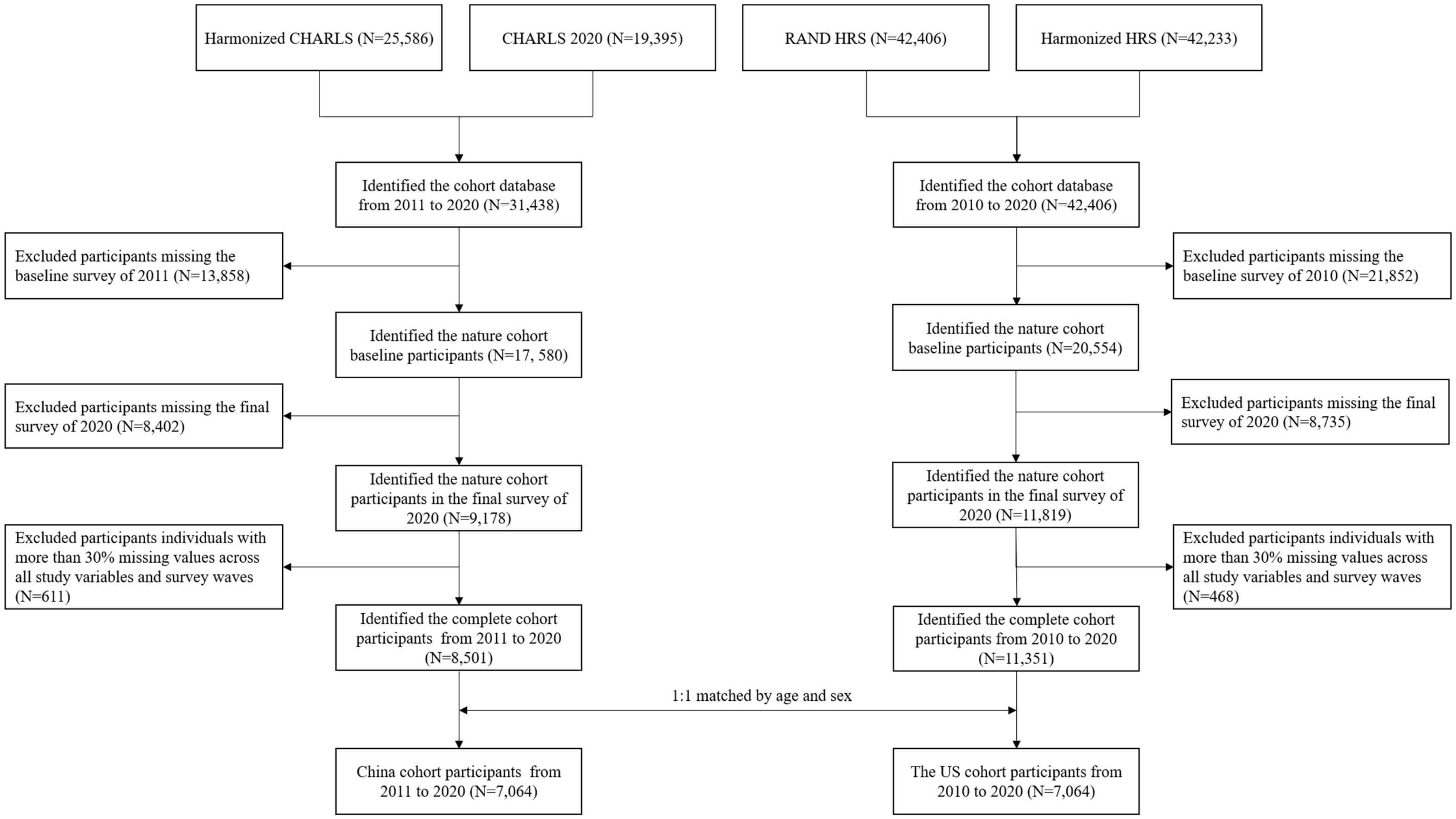

Figure S2. Multimorbidity network diagrams visualizing the various condition pairs that existed in wave 2

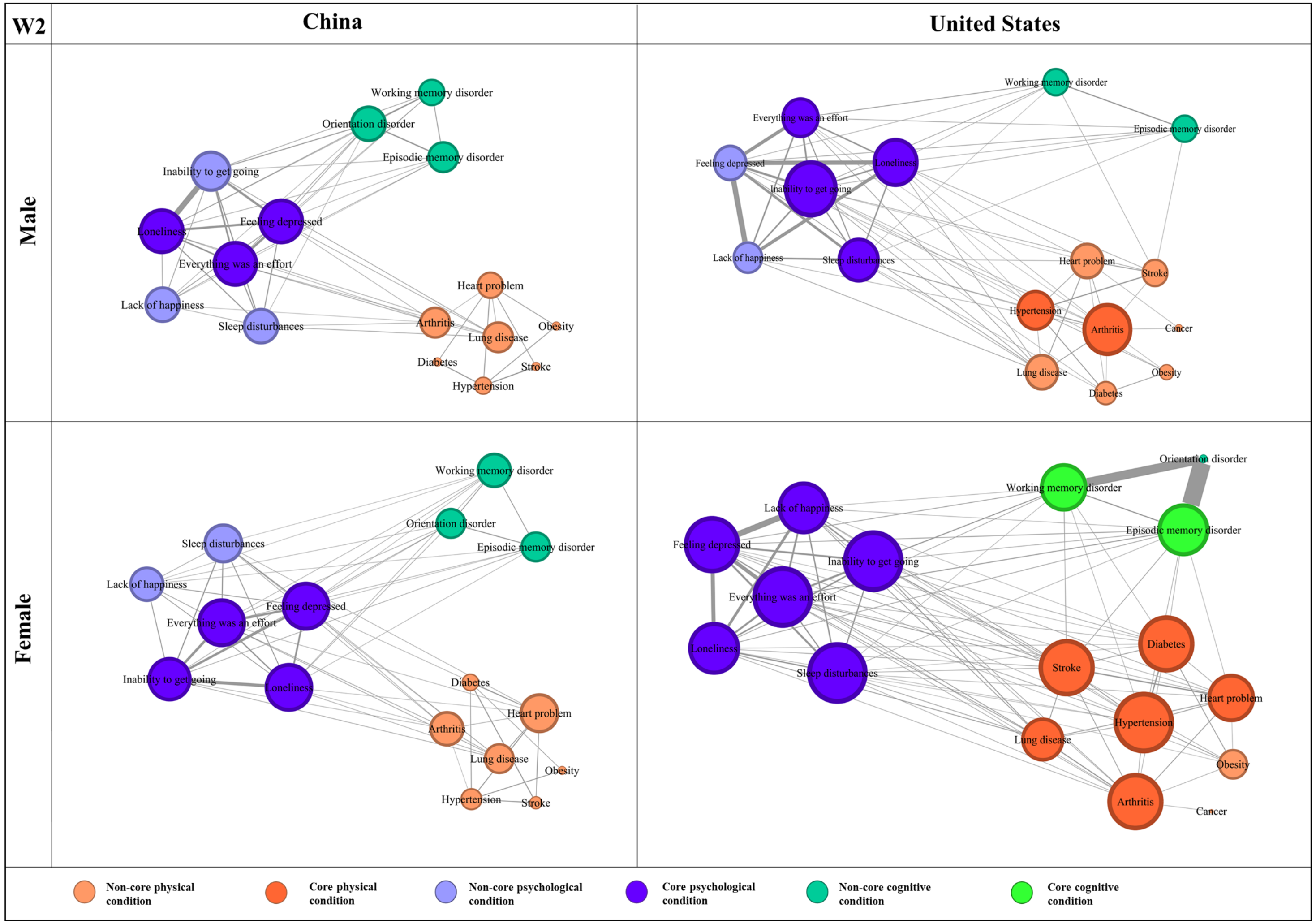

Figure S3. Multimorbidity network diagrams visualizing the various condition pairs that existed in wave 2

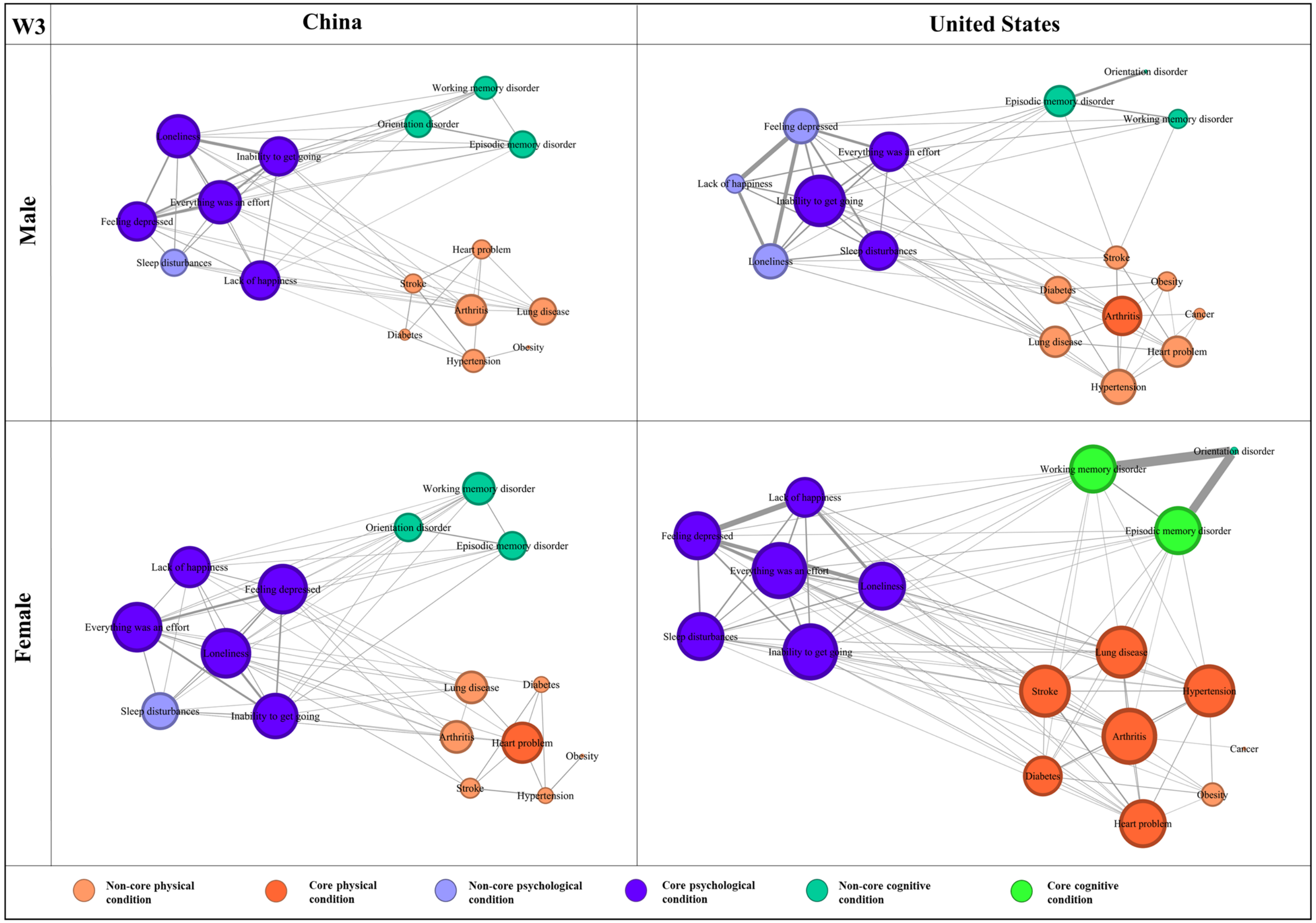

Figure S4. Multimorbidity network diagrams visualizing the various condition pairs that existed in wave 4

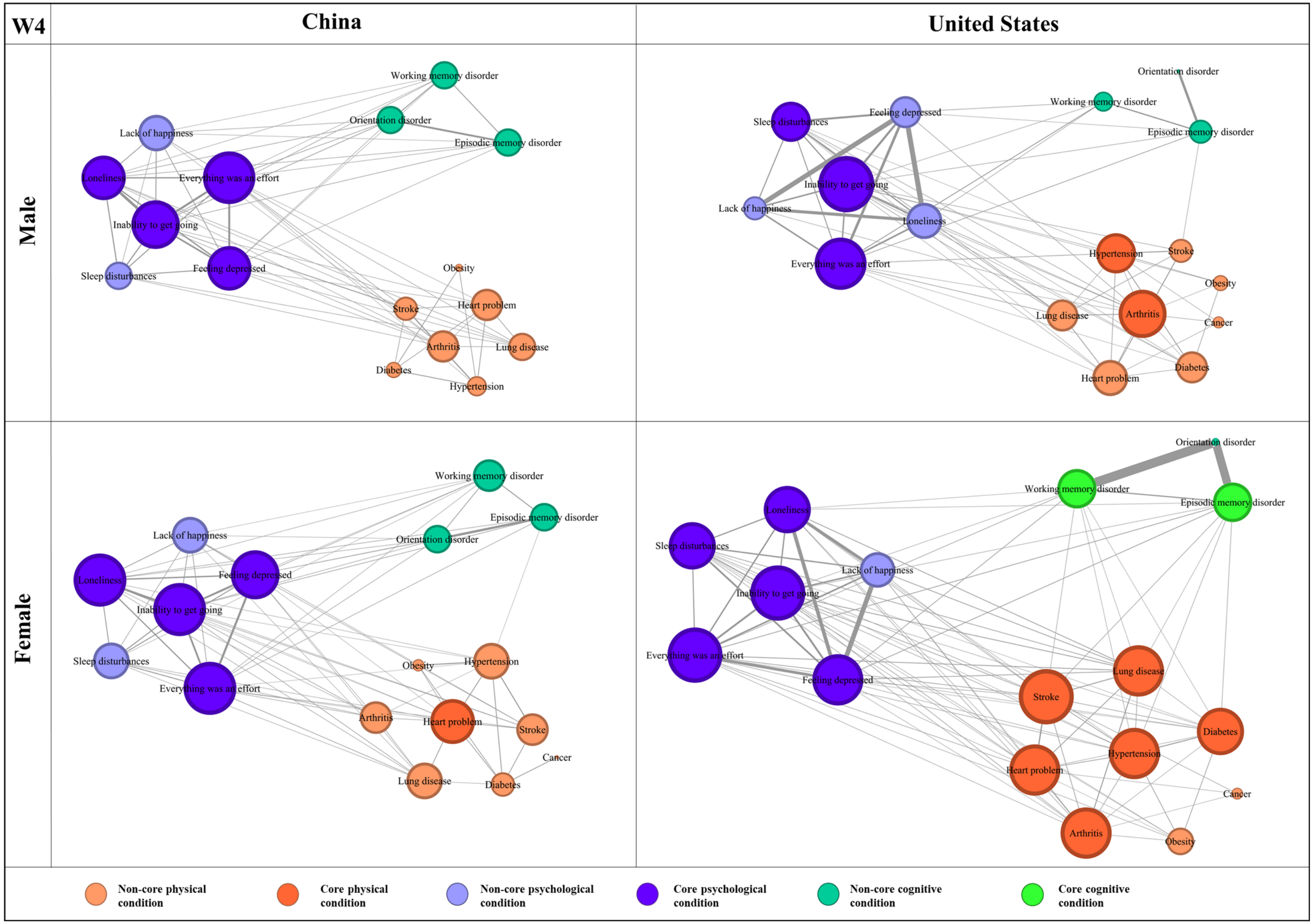

Figure S5. Multimorbidity network diagrams visualizing the various psychological and cognitive condition pairs that existed in each wave

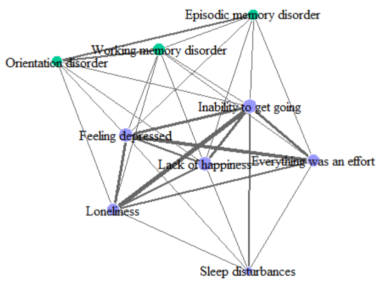

a) China male in 2011

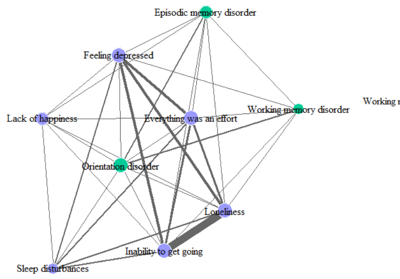

b) China male in 2013

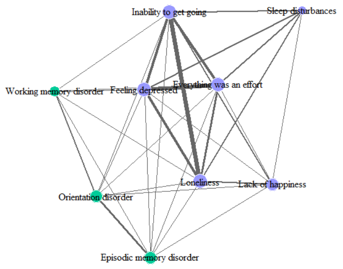

c) China male in 2015

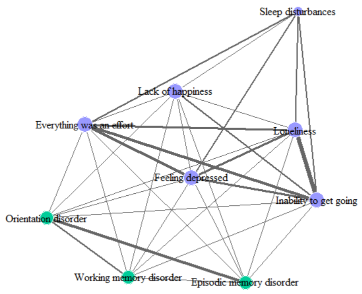

d) China male in 2018

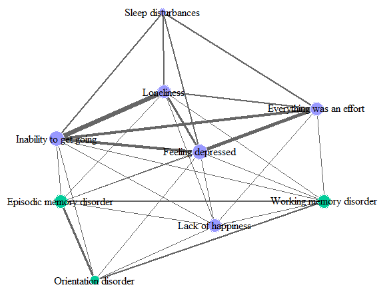

e) China male in 2020

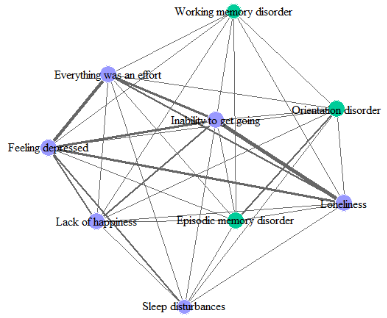

f) China female in 2011

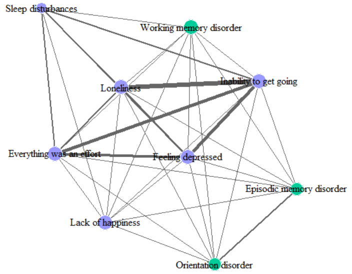

g) China female in 2013

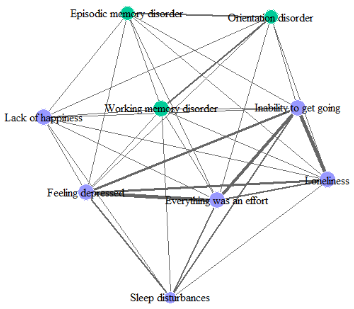

h) China female in 2015

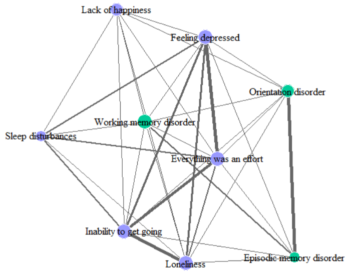

i) China female in 2018

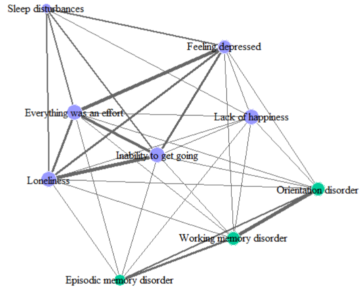

j) China female in 2020

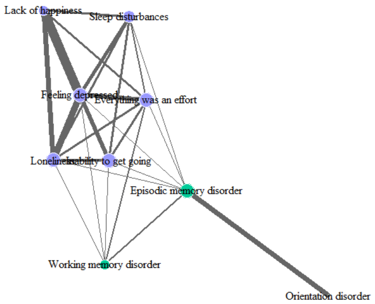

k) The US male in 2012

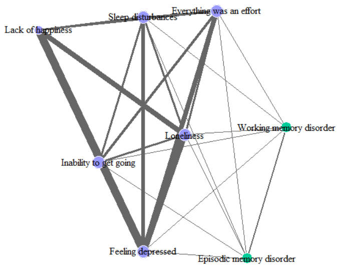

l) The US male in 2014

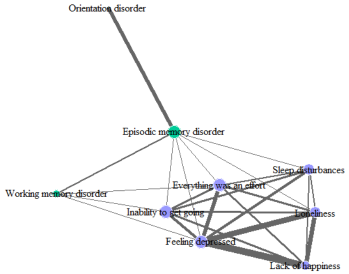

m) The US male in 2016

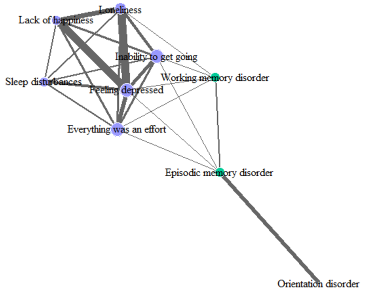

n) The US male in 2018

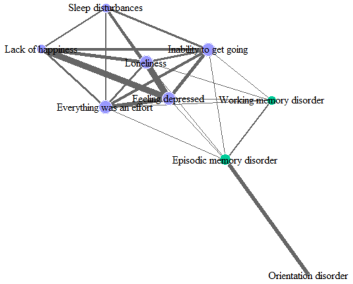

o) The US male in 2020

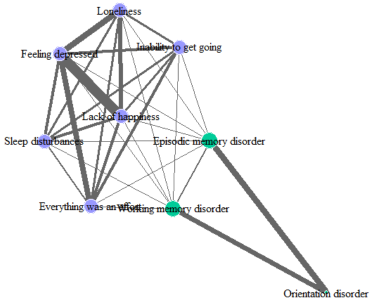

p) The US female in 2012

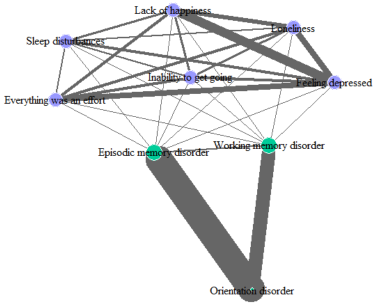

q) The US female in 2014

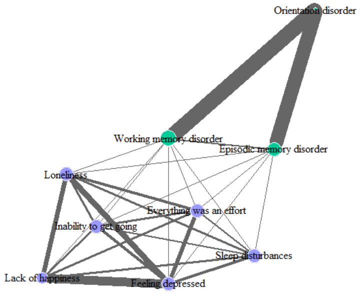

r) The US female in 2016

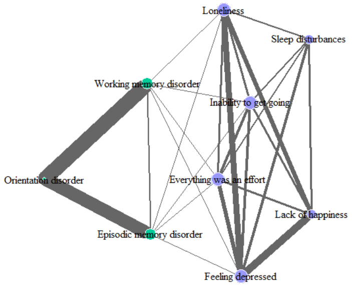

s) The US female in 2018

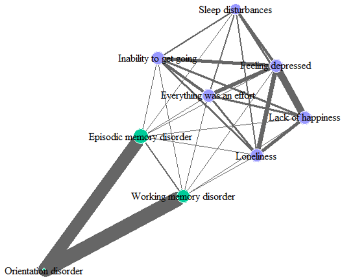

t) The US female in 2020

Figure S6. Multimorbidity network diagrams visualizing the various physical and cognitive condition pairs that existed in each wave

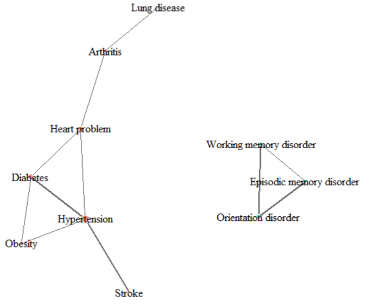

a) China male in 2011

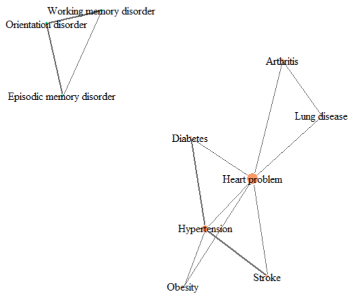

b) China male in 2013

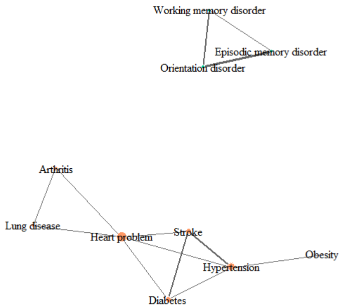

c) China male in 2015

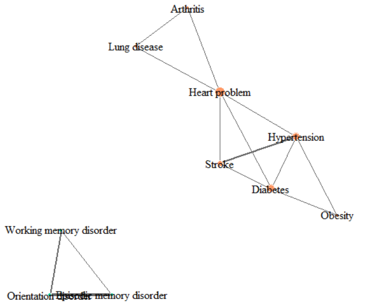

d) China male in 2018

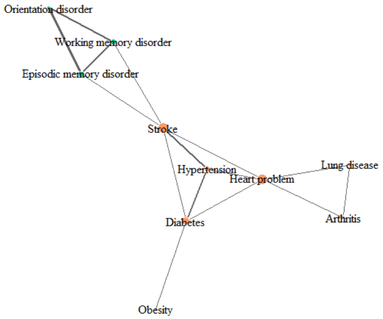

e) China male in 2020

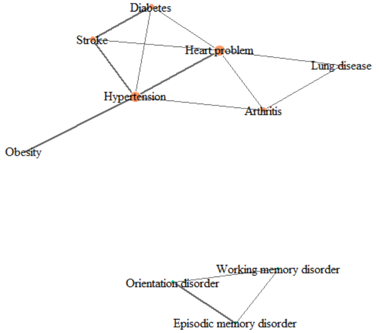

f) China female in 2011

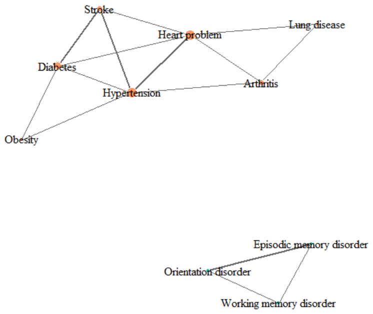

g) China female in 2013

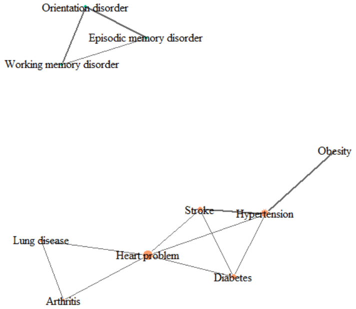

h) China female in 2015

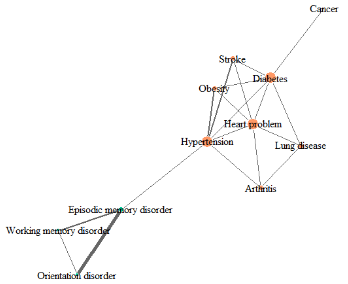

i) China female in 2018

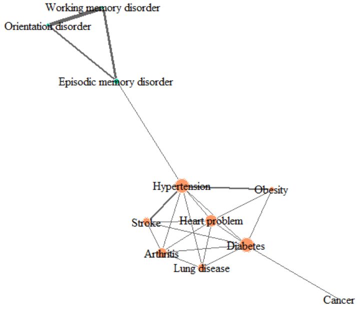

j) China female in 2020

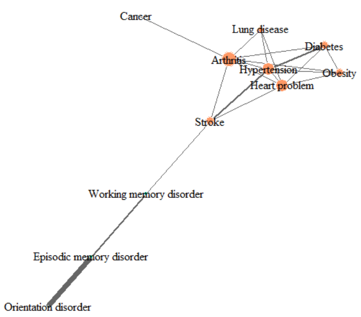

k) The US male in 2012

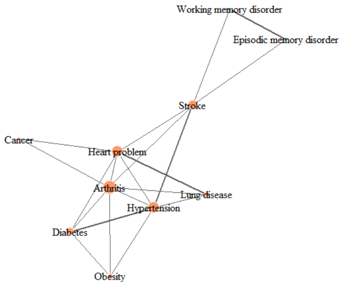

l) The US male in 2014

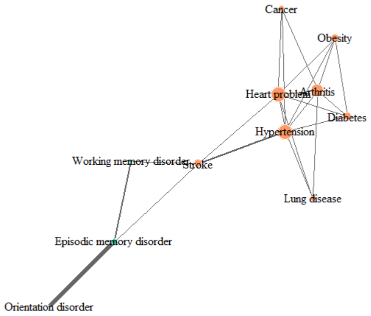

m) The US male in 2016

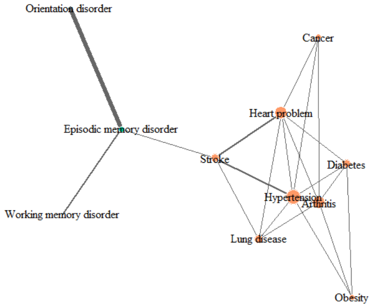

n) The US male in 2018

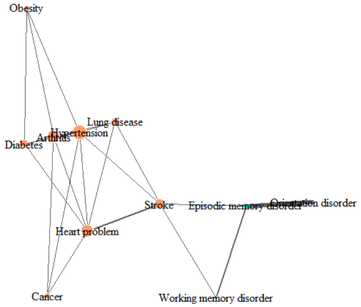

o) The US male in 2020

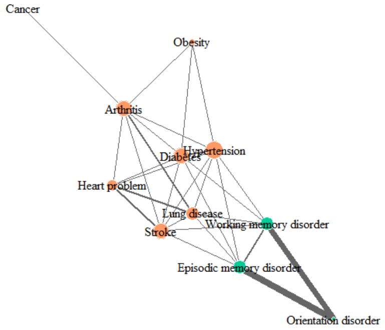

p) The US female in 2012

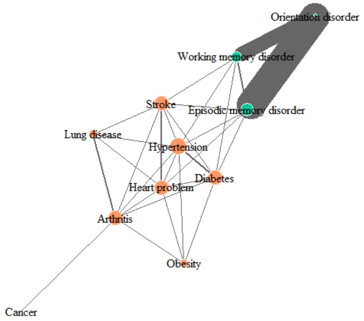

q) The US female in 2014

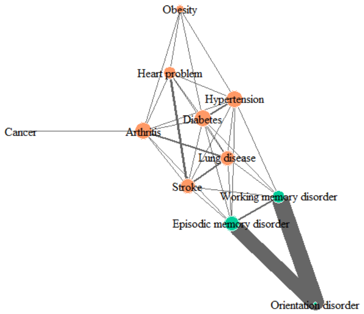

r) The US female in 2016

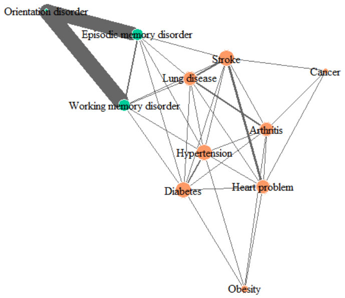

s) The US female in 2018

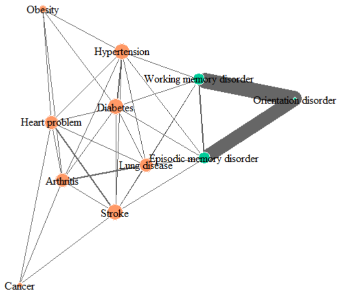

t) The US female in 2020

Figure S7. Multimorbidity network diagrams visualizing the various psychological and cognitive condition pairs that existed in each wave

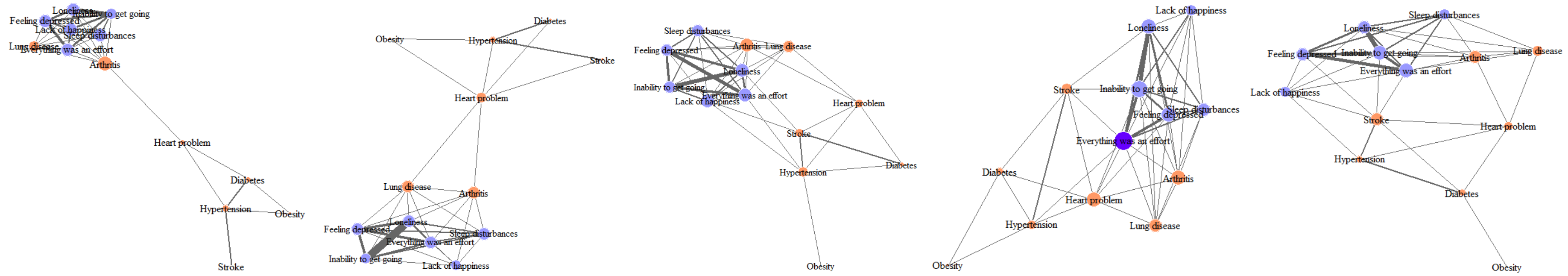

a) China male in 2011 b) China male in 2013 c) China male in 2015 d) China male in 2018 e) China male in 2020

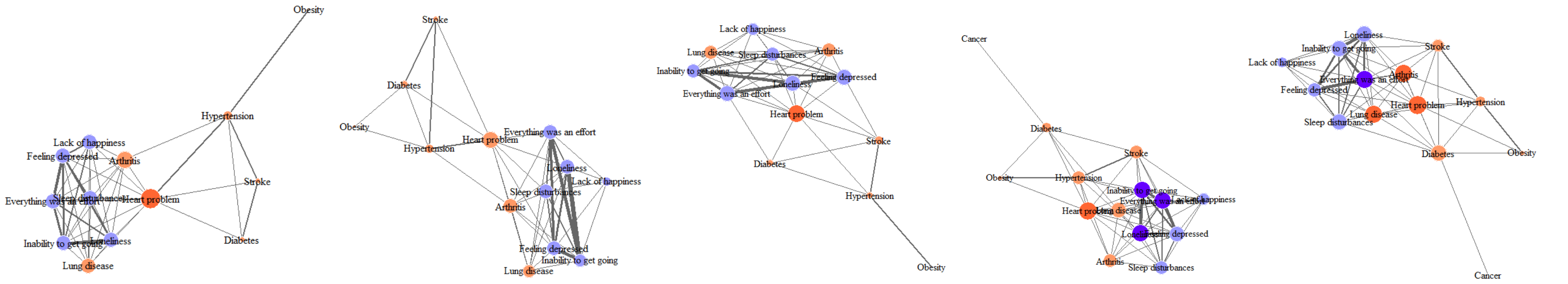

f) China female in 2011 g) China female in 2013 h) China female in 2015 i) China female in 2018 j) China female in 2020

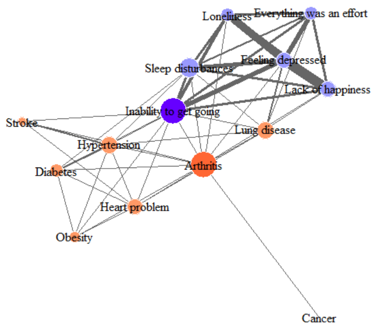

k) The US male in 2012

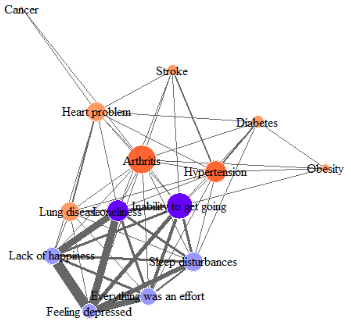

l) The US male in 2014

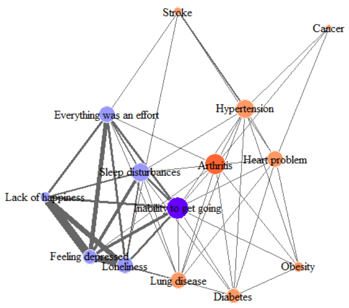

m) The US male in 2016

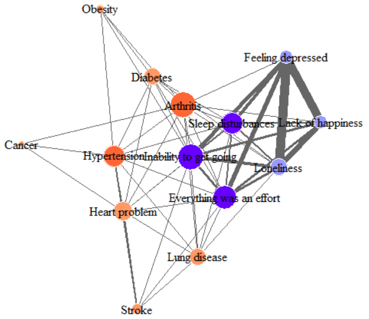

n) The US male in 2018

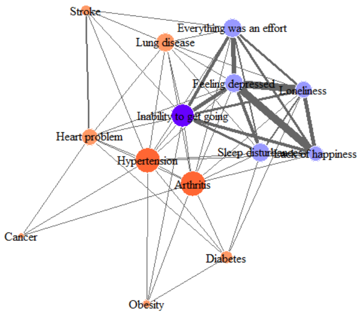

o) The US male in 2020

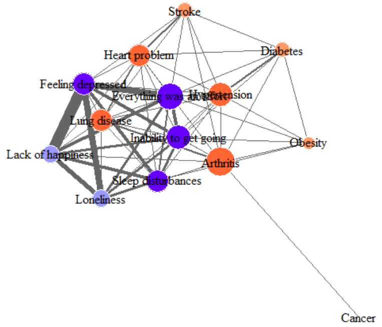

p) The US female in 2012

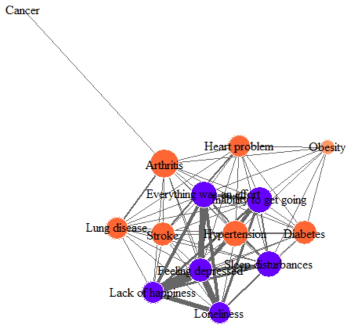

q) The US female in 2014

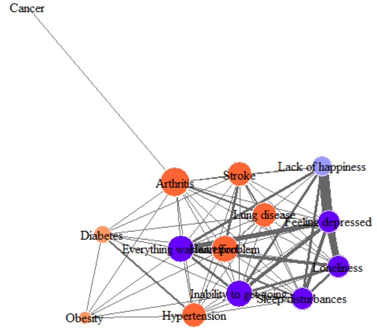

r) The US female in 2016

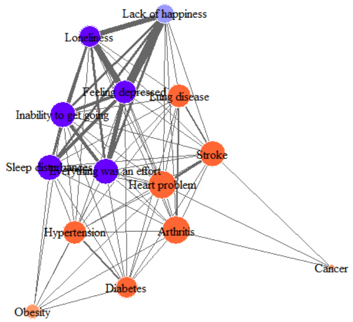

s) The US female in 2018

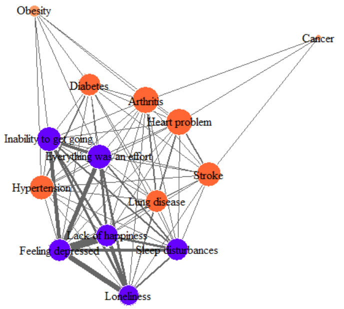

t) The US female in 2020

Figure S8. Sankey diagrams visualizing the evolution of the multimorbidity categories across five waves in A) China and B) the United States

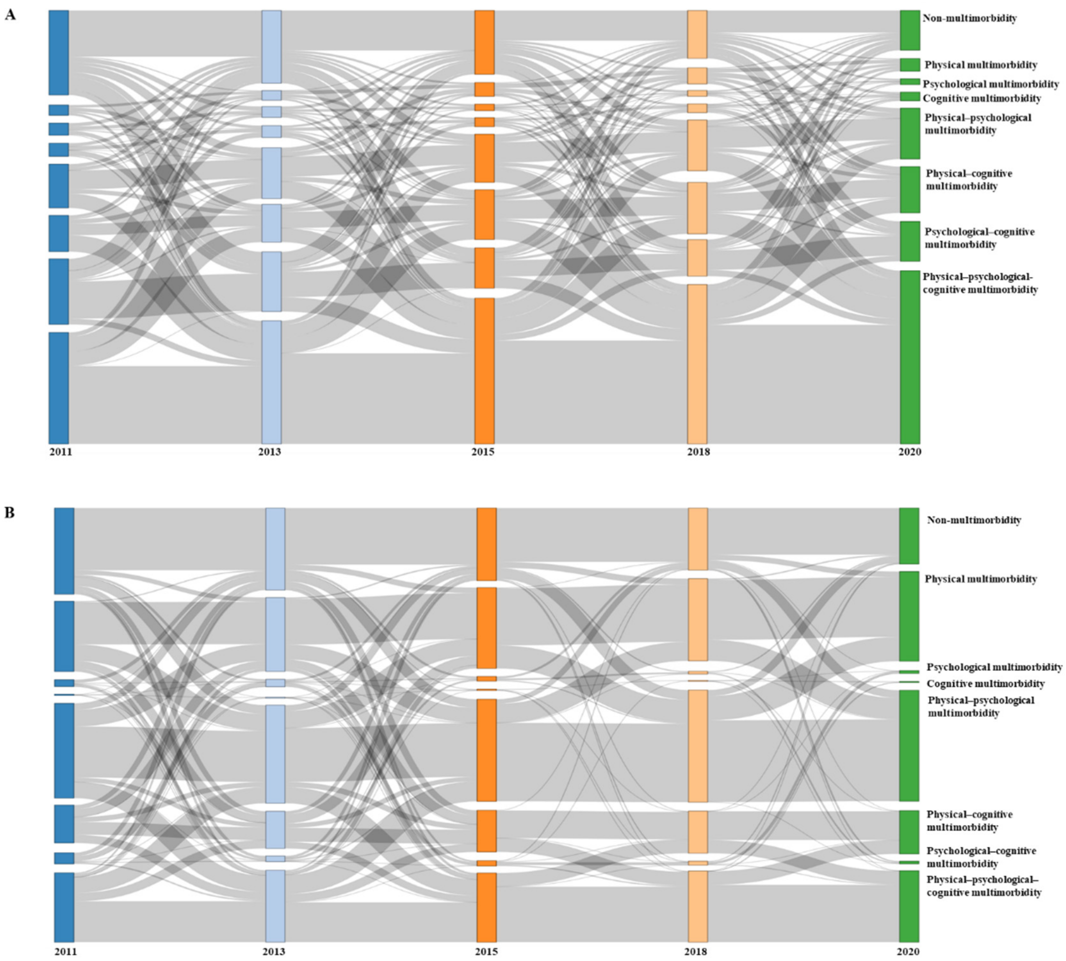

Reference

1. Zhao Y, Hu Y, Smith JP, Strauss J, Yang G: **Cohort profile: the China Health and Retirement Longitudinal Study (CHARLS)**. *Int J Epidemiol* 2014, **43**(1):61-68.
2. **Produced by the RAND Center for the Study of Aging, with funding from the National Institute on Aging and the Social Security Administration.** [https://hrsonline.isr.umich.edu/index.php?p=showdesc]
3. Plana-Ripoll O, Chen D, Laustsen LM, Momen NC: **The challenges at the core of multimorbidity research**. *Lancet Psychiatry* 2024.
4. Zhang L, Wei L, Fang Y: **Spatial-temporal distribution patterns and influencing factors analysis of comorbidity prevalence of chronic diseases among middle-aged and elderly people in China: focusing on exposure to ambient fine particulate matter (PM(2.5))**. *BMC Public Health* 2024, **24**(1):550.
5. Ou H, Yao Y, He Y: **Missing Data Imputation Method Combining Random Forest and Generative Adversarial Imputation Network**. *Sensors (Basel)* 2024, **24**(4).
6. Baars MA, van Boxtel MP, Dijkstra JB, Visser PJ, van den Akker M, Verhey FR, Jolles J: **Predictive value of mild cognitive impairment for dementia. The influence of case definition and age**. *Dement Geriatr Cogn Disord* 2009, **27**(2):173-181.
7. Karzmark P: **Validity of the serial seven procedure**. *Int J Geriatr Psychiatry* 2000, **15**(8):677-679.
8. Zhou S, Song S, Jin Y, Zheng ZJ: **Prospective association between social engagement and cognitive impairment among middle-aged and older adults: evidence from the China Health and Retirement Longitudinal Study**. *BMJ open* 2020, **10**(11):e040936.
9. Bao Y, Lu P, Wang M, Zhang X, Song A, Gu X, Ma T, Su S, Wang L, Shang X *et al*: **Exploring multimorbidity profiles in middle-aged inpatients: a network-based comparative study of China and the United Kingdom**. *BMC Med* 2023, **21**(1):495.
10. Aguado A, Moratalla-Navarro F, Lopez-Simarro F, Moreno V: **MorbiNet: multimorbidity networks in adult general population. Analysis of type 2 diabetes mellitus comorbidity**. *Sci Rep* 2020, **10**(1):2416.
11. Richardson RA, Keyes KM, Medina JT, Calvo E: **Sociodemographic inequalities in depression among older adults: cross-sectional evidence from 18 countries**. *Lancet Psychiatry* 2020, **7**(8):673-681.
12. Jafari-Koulaee A, Mohammadi E, Fox MT, Rasekhi A, Akha O: **Predictors of basic and instrumental activities of daily living among older adults with multiple chronic conditions**. *BMC Geriatr* 2024, **24**(1):383.
13. Storeng SH, Vinjerui KH, Sund ER, Krokstad S: **Associations between complex multimorbidity, activities of daily living and mortality among older Norwegians. A prospective cohort study: the HUNT Study, Norway**. *BMC Geriatr* 2020, **20**(1):21.
